# The *Pseudomonas aeruginosa N*-Acylhomoserine Lactone Quorum Sensing Molecules Target IQGAP1 and Modulate Epithelial Cell Migration

**DOI:** 10.1371/journal.ppat.1002953

**Published:** 2012-10-11

**Authors:** Thommie Karlsson, Maria V. Turkina, Olena Yakymenko, Karl-Eric Magnusson, Elena Vikström

**Affiliations:** 1 Division of Medical Microbiology, Department of Clinical and Experimental Medicine, Faculty of Health Sciences, Linköping University, Linköping, Sweden; 2 Division of Cell Biology, Department of Clinical and Experimental Medicine, Faculty of Health Sciences, Linköping University, Linköping, Sweden; Northwestern University, United States of America

## Abstract

Quorum sensing (QS) signaling allows bacteria to control gene expression once a critical population density is achieved. The Gram-negative human pathogen *Pseudomonas aeruginosa* uses *N*-acylhomoserine lactones (AHL) as QS signals, which coordinate the production of virulence factors and biofilms. These bacterial signals can also modulate human cell behavior. Little is known about the mechanisms of the action of AHL on their eukaryotic targets. Here, we found that *N*-3-oxo-dodecanoyl-*L*-homoserine lactone 3O-C_12_-HSL modulates human intestinal epithelial Caco-2 cell migration in a dose- and time-dependent manner. Using new 3O-C_12_-HSL biotin and fluorescently-tagged probes for LC-MS/MS and confocal imaging, respectively, we demonstrated for the first time that 3O-C_12_-HSL interacts and co-localizes with the IQ-motif-containing GTPase-activating protein IQGAP1 in Caco-2 cells. The interaction between IQGAP1 and 3O-C_12_-HSL was further confirmed by pull-down assay using a GST-tagged protein with subsequent Western blot of IQGAP1 and by identifying 3O-C_12_-HSL with a sensor bioassay. Moreover, 3O-C_12_-HSL induced changes in the phosphorylation status of Rac1 and Cdc42 and the localization of IQGAP1 as evidenced by confocal and STED microscopy and Western blots. Our findings suggest that the IQGAP1 is a novel partner for *P.aeruginosa* 3O-C_12_-HSL and likely the integrator of Rac1 and Cdc42- dependent altered cell migration. We propose that the targeting of IQGAP1 by 3O-C_12_-HSL can trigger essential changes in the cytoskeleton network and be an essential component in bacterial – human cell communication.

## Author Summary

The human pathogen *Pseudomonas aeruginosa* and other bacteria communicate with each other using quorum sensing (QS). This is important for their growth, virulence, motility and the formation of biofilms. Furthermore, eukaryotic cells “listen and respond” to QS signaling, but the exact mechanisms and receptors on mammalian cells have not been identified. We have previously shown that *N*-acylhomoserine lactones (AHL) alter epithelial barrier functions and increase chemotaxis in human neutrophils. We show here that 3O-C_12_-HSL modulates the migration of epithelial cells in a dose- and time-dependent manner. Using newly designed and validated biotin- and fluorescein-based 3O-C_12_-HSL probes we identified the IQ-motif-containing GTPase-activating protein IQGAP1 as a human target of 3O-C_12_-HSL. We propose that the interaction between IQGAP1 and 3O-C_12_-HSL provides a novel mechanism for its mode of action on eukaryotic cells, and by affecting the distribution of IQGAP1 and phosphorylation of Rac1 and Cdc42, upstream effectors of filamentous actin remodeling, also cell migration. We suggest that recognition of IQGAP1 by 3O-C_12_-HSL is a very early event in the communication between bacteria and human epithelial cells.

## Introduction

**Figure 1:**
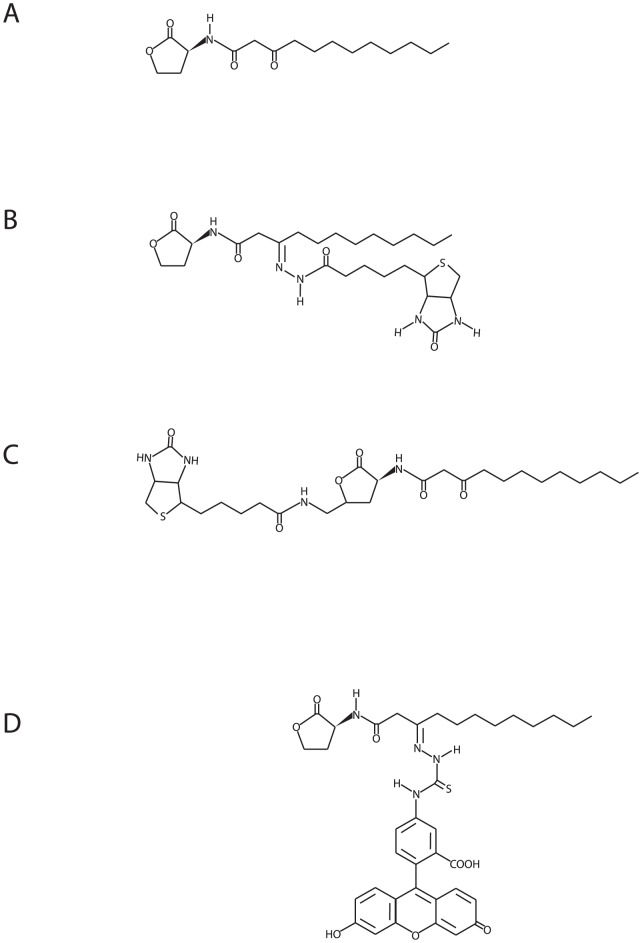
Synthetic AHL molecules used in this study. (A) *N*-3-oxo-dodecanoyl-L-homoserine lactone C_16_H_27_NO_4_, MW 297.4 (3O-C_12_-HSL) which is structurally and functionally identical to those obtained from *P. aeruginosa* cultures; (B) biotin-conjugated probe *N*-dodecanoyl-L-homoserine lactone-3-hydrazone-biotin C_26_H_43_N_5_O_5_S, MW 537.7 (3O-C_12_-HSL-3H-biotin) and (C) *N*-dodecanoyl-L-homoserine lactone-biotin C_38_H_41_N_3_O_9_S, MW 552.7 (3O-C_12_-HSL-biotin); (D) Fluorescently-tagged probe *N*-dodecanoyl-L-homoserine lactone-3-hydrazone-fluorescein, C_37_H_40_N_4_O_8_S, MW 700.8 (3O-C_12_-HSL-FITC).

Quorum sensing (QS) is a population-density-dependent signaling system that primarily enables bacteria to control the expression of certain genes. Bacteria constitutively produce, release and detect distinct low-molecular-weight QS signal molecules, which bind to intracellular receptors in the bacteria to coordinate transcription of QS-controlled genes [1]. In Gram-negative human pathogen *Pseudomonas aeruginosa*, there are two chemically distinct but subordinated QS systems that are *N*-acylhomoserine lactone- (AHL) and 2-alkyl-4-quinolone-dependent, respectively. Two AHL molecules are produced by *P.aeruginosa*, *N*-3-oxo-dodecanoyl-*L*-homoserine lactone (3O-C_12_-HSL) (Figure 1A) and *N*-butyryl-*L*-homoserine lactone (C_4_-HSL), which directly or indirectly control the expression of multiple virulence factors, secondary metabolites, swarming motility and biofilm development [2], [3].

Bacterial QS signals also influence the behavior of eukaryotic cells in a process called inter-kingdom signaling [4]. To date, rather little is known about the exact mechanisms of the action of AHL on eukaryotic cells and their direct target(s) or receptor(s), but it has been suggested that AHL acts through multiple signaling pathways [5]. There is evidence that lipophilic 3O-C_12_-HSL molecules with a long acyl chain and an intact homoserine lactone ring (Figure 1A) can interact directly with phospholipids in model membrane systems and in Jurkat T-cell membranes [6]. On entering mammalian cells [7], [8], 3O-C_12_-HSL may utilize intracellular nuclear peroxisome proliferator-activated receptors (PPAR) to affect transcriptional activity and NF-κB signaling [9], [10]. However, to interact with intracellular molecules in eukaryotic cells, it is likely that 3O-C_12_-HSL first targets membrane-associated proteins. Shiner et al. [11] have suggested the existence of a membrane-associated receptor, which likely after binding to 3O-C_12_-HSL phosphorylates phospholipase C and evokes an increase in intracellular calcium [12]. Recent work on plasma membrane interaction with 3O-C_12_-HSL [6] supports this hypothesis.

Apart from efforts to find a putative receptor for 3O-C_12_-HSL, recent studies have investigated its effects on mammalian host cells - for immune cells, fibroblasts, vascular endothelial cells, alveolar and intestinal epithelial cells [3], [13].

Intestinal epithelial cells form a semi-permeable barrier separating the luminal content from underlying tissues and contribute to the maturation of the immune system and the development of immune response or tolerance, a process that is critical to normal growth, development and disease prevention [14]. The epithelial lining consists of a monolayer of columnar cells, which are constantly moving and renewed. Its function can be perturbed by bacteria, viruses and their toxins, resulting, for instance, in oxidative stress and inflammation. After injury, the intestinal epithelium undergoes a wound- healing process which is dependent on the balance of migration, proliferation and differentiation of the cells within and around the wounded area [15]. Restitution of the epithelium requires extensive remodeling and reorganization of the actin cytoskeleton, regulated by the Rho family of small GTPases, such as Rho, Rac and Cdc42. Rho principally controls the formation of focal adhesion and stress fibers, Rac regulates the formation of lamellipodia protrusions and membrane ruffles, and Cdc42 triggers filopodial extensions [16], [17]. Rho GTPases cycle between an active and inactive status by binding GTP and by hydrolysis of GTP to GDP [18], acting as molecular “on-off” switches. Rho GTPase signaling can also be modulated by their phosphorylation state via Akt1 kinase [19]. Thus, Ser-71 phosphorylation of Rac1 and Cdc42 modulates their interaction with bacterial Rho-glucosylating toxins [20], positioning these GTPases as target structures for microbial virulence factors and for bacteria-host interactions in general.

In the present study, we assessed the effect of 3O-C_12_-HSL on migration and proliferation in human intestinal epithelial Caco-2 cells. We also analyzed 3O-C_12_-HSL affinity for Caco-2-derived proteins using 3O-C_12_-HSL biotin probes (Figure 1B and C) and LC-MS/MS and found that 3O-C_12_-HSL targets the IQ-motif-containing GTPase-activating protein IQGAP1. We also confirmed the interaction between IQGAP1 and 3O-C_12_-HSL in a pull-down assay. Moreover, using advanced confocal and superresolution microscopy, we assessed cellular co-localization of IQGAP1, phosphorylated Rac1/Cdc42 and 3O-C_12_-HSL fluorescently-tagged molecules (Figure 1D).

## Results

### 3O-C_12_-HSL modulates cell migration in a dose- and time-dependent manner

**Figure 2:**
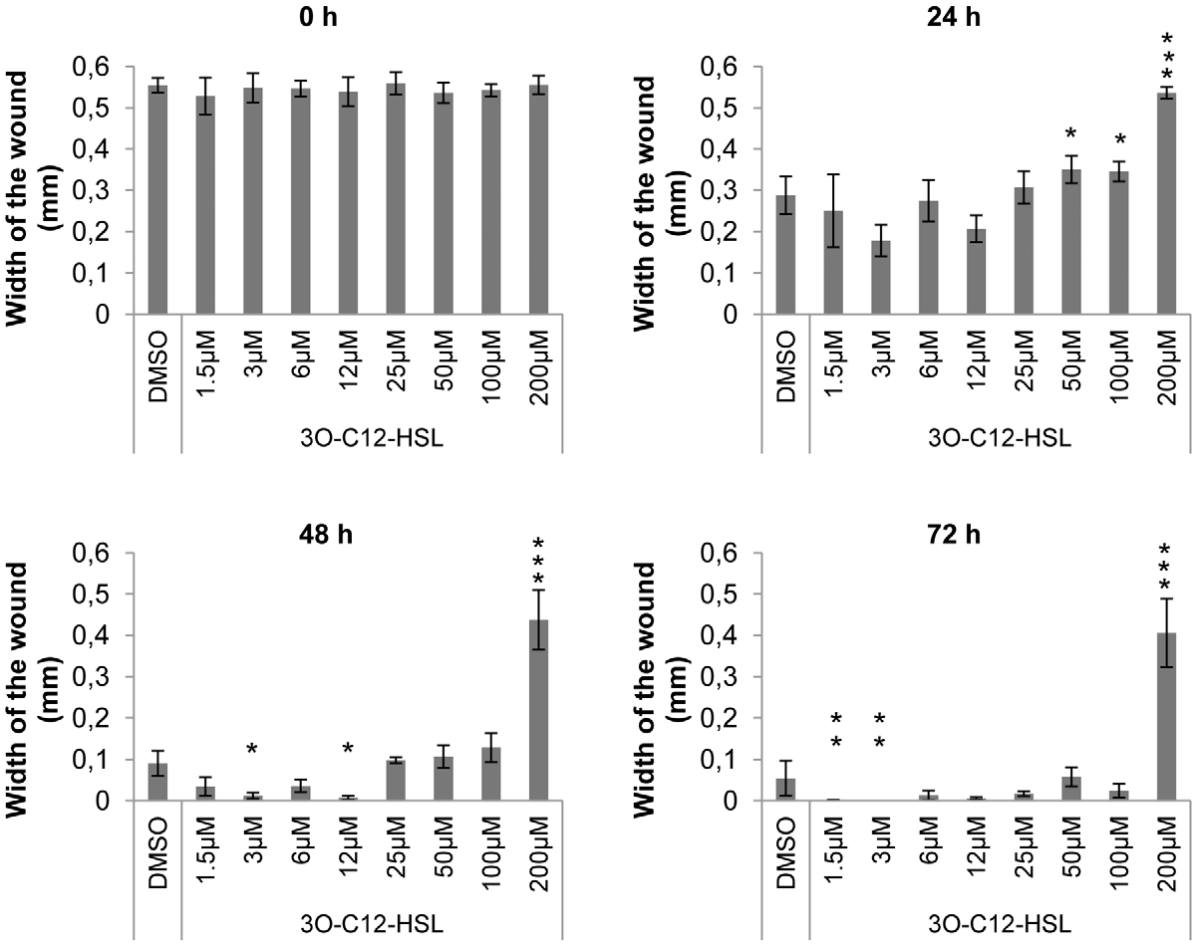
3O-C_12_-HSL modulates migration of Caco-2 cells in a dose- and time-dependent manner. Caco-2 cells were cultured in μ-dishes with Ibidi insert until monolayers were confluent, and the insert was removed to get two cell patches with a rectangular cell free gap (500±50 µm width). Cells were treated with 0.018% DMSO (diluent control), or 1.5, 6, 12, 25, 50, 100 and 200 µM 3O-C_12_-HSL and allowed to migrate. For each dish, 4 images of the migrated cells in the gap area were taken at 0, 24, 48 and 72 h. Migration rates were calculated by measuring the distance between cell monolayer patches (six measurements per image and four images per dish for the each time point) using Image J software. Shown is the mean ± standard errors of at least six independent experiments performed on separate days from different cell passages. Significant differences (* - *P*≤0.05; ** - *P*≤0.01;*** - *P*≤0.001) in mean for migration rate compared with values for cells in the control group as calculated by Student's *t* test.

To study the effect of 3O-C_12_-HSL on epithelial cell motility we used Ibidi migration-wound healing assays. We observed that 3O-C_12_-HSL at high concentrations inhibited Caco-2 cells migration (Figure 2). Compared with the control cells, which were treated with 0.018% DMSO as a diluent control, 200 µM 3O-C_12_-HSL significantly decreased the migration rate of Caco-2 cells after treatment for 24, 48 and 72 h. During this time, the width of the wound remained almost unchanged (between 0.5±0.02 mm and 0.4±0.08 mm), and cells failed to elicit migration and wound-healing. Quantitatively, 200 µM 3O-C_12_-HSL induced a 1.6-, 4-, and 8-fold greater suppression of migration after 24, 48 and 72 h, respectively. For 50 and 100 µM 3O-C_12_-HSL, significant suppressive effects were observed at 24 h only and reached a 1.2-fold effect; longer incubation times did not yield a further response. By contrast, the migration rates of Caco-2 cells treated with 1.5, 3 and 12 µM 3O-C_12_-HSL were significantly promoted after 48 and 72 h, whereas the effect of 25 µM 3O-C_12_-HSL was similar to the DMSO control. To investigate whether the 3O-C_12_-HSL-induced effects were dependent on the substrate coating, we set up a modified migration assay from Oris, where cell monolayers were cultured on tissue culture-treated, rat tail collagen- and human fibronectin-coated surfaces and circular-wounded. Here, we confirmed that 3O-C_12_-HSL at the high concentration inhibited Caco-2 cell migration (Figure S2). Thus, after 72-h treatment with 200 µM 3O-C_12_-HSL, the Caco-2 cell migration rate was significantly decreased in all types of coating; the diameter of the wound was 1.25-, 2.5- and 1.1-fold larger in monolayers growing on tissue culture-, collagen- and fibronectin-coated surfaces, respectively, compared with the DMSO control. The migration rate of Caco-2 cells treated with 12 µM C_12_-HSL was similar to that of the controls, i.e. untreated cells (not shown on Figures 2 and S2) and 0.018% DMSO-treated cells. Incidentally, wound healing was more rapid on a collagen-coated surface. The inhibitory effect of 100 and 200 µM 3O-C_12_-HSL on Caco-2 cell migration appeared not to be the result of decreased cell proliferation and viability (Figure S3 and Protocol S1). Taken together, this shows that 3O-C_12_-HSL affects epithelial Caco-2 cell migration in a dose- and time-dependent manner but does not promote changes in cell proliferation or viability. However, low concentrations of native, or biotin- or fluorescently-tagged 3O-C_12_-HSL probes were used in the further LC-MS/MS, pull-down and imaging experiments.

### 3O-C_12_-HSL affinity for IQGAP1 and 2

**Figure 3:**
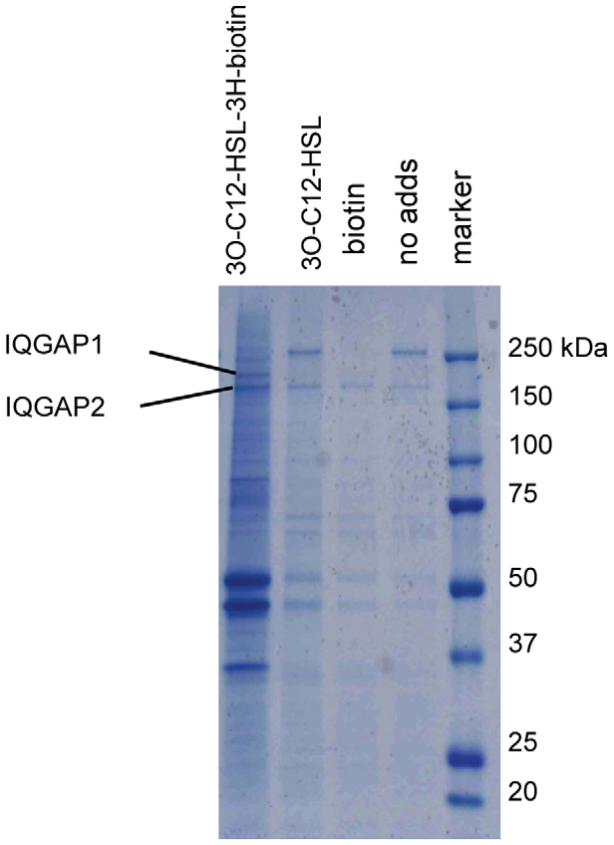
SDS-PAGE analysis of 3O-C_12_-HSL-3H-biotin complexes from cytoplasmic fraction of Caco-2 cells. The cytoplasmic fraction was incubated with 0.05 mg 3O-C_12_-HSL-3H-biotin, 0.05 mg 3O-C_12_-HSL, and 4 µg biotin or without any additions (as controls). Streptavidin agarose resin-captured complexes were analyzed by SDS-PAGE and subsequently stained with PageBlue protein staining solution. Displayed is a representative gel from one of three independent experiments performed on separate days from different reactions, fraction isolation and cell passages. Indicated bands represent proteins IQGAP1 and 2 respectively identified by in-gel digestion and LC-MS/MS analysis as shown in Table 1. Peptide identification views from MASCOT MS data analyses of IQGAP1 and 2 are shown in supporting information (Dataset S1 and S2).

Because cell migration is driven by a highly complex signaling network and 3O-C_12_-HSL has been shown to markedly affect cell motility in both epithelial cells and neutrophils [12], we wanted to identify a potential interaction partner for 3O-C_12_-HSL. To do so, we used the total-cell lysate as well as the cytoplasmic and membrane fractions obtained from Caco-2 cell monolayers. The fractions were incubated with 0.05 mg 3O-C_12_-HSL-3H-biotin (Figure 1B), and the complexes were captured with a streptavidin-agarose resin. As controls, the corresponding cellular fractions were incubated with 0.05 mg 3O-C_12_-HSL, 4 µg biotin, or no additions at all. The resulting resin-bound complexes were analyzed by SDS-PAGE, and protein bands of interest were cut from the gels, digested and subjected to peptide analysis by LC-MS/MS and protein identification. The experiments were repeated at least three times, and one specific protein band was reproducibly detected in the cytoplasmic sample which contained resin-bound 3O-C_12_-HSL-3H-biotin (Figure 3). After in-gel-digestion and mass spectrometry analyses of the obtained peptides, we were able to identify the Ras GTPase-activating-like protein, IQGAP1 (Figure 3, Table 1, Dataset S1 and S2). This band was absent in all corresponding controls. Moreover, IQGAP2 was identified from the band below of the same cytoplasmic fraction and was absent in the bands of corresponding controls (Figure S4, Table S1). The remaining protein profiles for total-cell lysate as well as cytoplasmic and membrane fractions were almost similar between 3O-C_12_-HSL-3H-biotin and three control incubations and were therefore regarded as unspecific binding of abundant cytoskeleton network proteins or false positive in the affinity procedure (Figure S4, Table S1) [21], [22]. Furthermore, we analyzed 3O-C_12_-HSL affinity for Caco-2-derived proteins using the other probe, 3O-C_12_-HSL-biotin (Figure 1C) and LC-MS/MS, but no IQGAP1 or 2 were detected (Figure S5, Table S2). This data could be explained by the fact that 3O-C_12_-HSL molecule with a modified homoserine lactone ring is less biologically active and probably incapable of interacting with its target molecules [6], [23]. For the further analyses, we selected IQGAP1 because of its strong band and the number of identified tryptic peptides (24 for IQGAP1and 8 for IQGAP2). Peptide identification views from MASCOT MS data analyses of IQGAP1 and 2 are shown in the supporting information (Dataset S1 and S2). A protein with MW 36 kDa indentified as glyceraldehyde-3-phosphate dehydrogenase (GAPDH) was selected as loading protein control in the subsequent immunoblot experiments.

**Table 1:** IQGAP tryptic peptides identified in 3O-C_12_-HSL-3H-biotin affinity complexes from cytoplasmic fraction of Caco-2 cells using LC-MS/MS.

Position	Peptide sequence	M/Z	Z	Score/E-value
**IQGAP1**				
1224–1230	NLGSIAK	702.5	1	27/0.37
1391–1397	LIVDVIR	414.3	2	36/0.023
1054–1060	mVVSFNR	434.7	2	27/0.15
81–88	LGNFFSPK	455.3	2	75/3e-06
1028–1035	TALQEEIK	466.3	2	51/0.0011
1383–1390	TILLNTKR	479.9	2	26/0.34
1466–1475	LTELGTVDPK	536.9	2	55/0.00041
989–997	LIFQmPQNK	567.9	2	32/0.069
192–201	YGIQmPAFSK	579.3	2	59/0.00014
1506–1516	LQQTYAALNSK	618.9	2	89/1.2e-07
389–401	LAAVALINAAIQK	648.5	2	69/1.2e-05
446–477	ALESGDVNTVWK	659.9	2	78/1.8e-06
1443–1455	SVKEDSNLTLQEK	497.6	3	29/0.14
1443–1455	SVKEDSNLTLQEK	746.0	2	33/0.052
739–791	EQLWLANEGLITR	772.0	2	101/8.5e-09
857–870	TLINAEDPPmVVVR	785.5	2	75/3.3e-06
755–787	QIPAITCIQSQWR	801.0	2	53/0.00057
1038–1053	VDQIQEIVTGNPTVIK	877.6	2	101/4.8e-09
539–556	ILAIGLINEALDEGDAQK	942.1	2	105/3e-09
539–556	ILAIGLINEALDEGDAQK	942.1	2	90/1.1e-07
623–641	FALGIFAINEAVESGDVGK	969.1	2	105/3e-09
1587–1604	NVIFEISPTEEVGDFEVK	1026.5	2	113/4.8e-10
568–585	LEGVLAEVAQHYQDTLIR	685.8	3	58/6.5e-05
175–191	VDFTEEEINNmKTELEK	695.7	3	57/0.00022
2–25	Ac-SAADEVDGLGVARPHYGSVLDNER	857.1	3	89/1.1e-07
1398–1422	FQPGETLTEILETPATSEQEAEHQR	947.8	3	59/0.00011
**IQGAP2**				
506–514	LGDSESVSK	461.4	2	53/0.00036
554–562	SSDILSVLK	961.6	1	24/0.35
807–814	LREEVVTK	487.4	2	41/0.0069
92–99	IYDVEQTR	512.4	2	62/4.3e-05
902–910	LIFQmPQNK.	567.9	2	41/0.0055
91–99	KIYDVEQTR	576.4	2	61/4.7e-05
951–966	VDQVQDIVTGNPTVIK	863.7	2	86/7.7e-08
857–872	GGEmEILNNTDNQGIK	875.0	2	28/0.1

The sequences of the peptides obtained after in-gel digestion of eluate after 3O-C_12_-HSL-3H-biotin affinity chromatography. M/Z - mass over charge ratio; Z – ion charge; lower-case m in the sequences specifies oxidized methionine residue and Ac- designates the N-terminal acetylation.

### 3O-C_12_-HSL binds to IQGAP1

**Figure 4:**
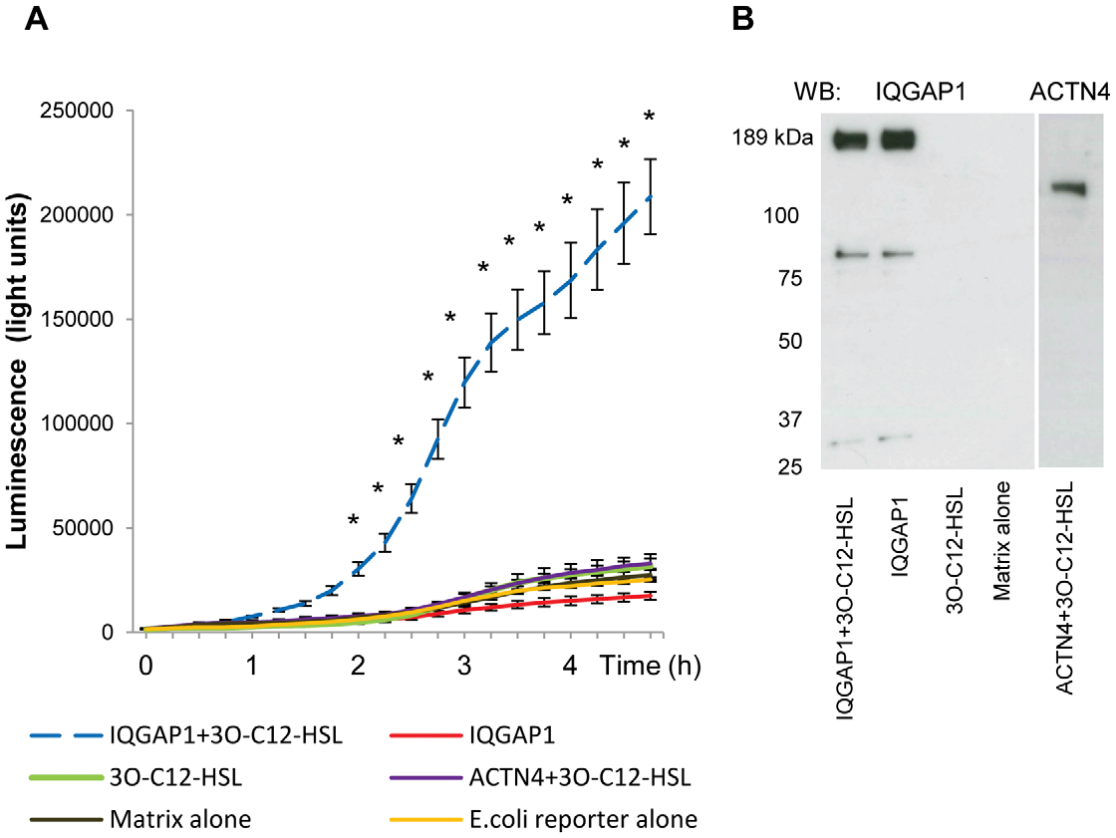
IQGAP1 and 3O-C_12_-HSL interaction analysis using a GST pull-down assay. (A) Detection of 3O-C_12_-HSL in the eluates using *E.coli* JM109 pSB1075 lux reporter bioassay. Eluates from matrix beads were from the following pull-down reaction components: GST-IQGAP1 full-length and 3O-C_12_-HSL (dashed blue line IQGAP1+3O-C_12_-HSL); GST-IQGAP1 full-length (red line IQGAP1); 3O-C_12_-HSL (green line 3O-C_12_-HSL; GST-actinin-4 full-length and 3O-C_12_-HSL (lilac line ACTN4+3O-C_12_-HSL); and without adding (brawn line Matrix alone). As an additional control for the bioassay bacteria reporter in LB medium (not shown) or bacteria reporter in LB medium containing elution buffer, TNGT was used (yellow line *E.coli* reporter alone). Luminescence was measured during 4.5-h growth. Shown is the mean ± standard errors of at least three independent experiments performed on separate days from different reactions. Significant differences (*) in mean for luminescence compared with values for control groups as calculated by Student's *t* test. (B) Detection of IQGAP1 and actinin 4 in the eluates, after SDS-PAGE and Western blot analysis. Eluates described in (A) were analyzed here. The data are from one representative of at least three independent experiments.

To confirm LS-MS/MS results on the 3O-C_12_-HSL affinity for IQGAP1, we performed pull-down assay with purified GST-tagged full-length IQGAP1 [24] and probed its ability to catch an interacting target, 3O-C_12_-HSL. The eluates from pull-down reactions were analyzed in *E.coli* JM109 pSB1075 reporter bioassay to detect 3O-C_12_-HSL (Figure 4A) and in Western blot to identify GST-fusion proteins (Figure 4B). We found that 3O-C_12_-HSL appeared to bind to IQGAP1, but the exact location of the binding site on IQGAP1 has not yet been determined. Based on luminometry, it was estimated that the binding affinity of IQGAP1 and 3O-C_12_-HSL was approximately up to 8-fold higher than for GST-actinin 4. This latter GST-tagged protein served as a control to check that 3O-C_12_-HSL did not bind to the GST itself or non-specifically to any protein. Consistent with this, 3O-C_12_-HSL did not bind to the matrix. The lower molecular weight bands seen in the IQGAP1 lanes likely represent degradation fragments of the fusion protein (Figure 4B).

### 3O-C_12_-HSL modulates the level and distribution of IQGAP1 and phosphorylation of Rac1/Cdc42

**Figure 5:**
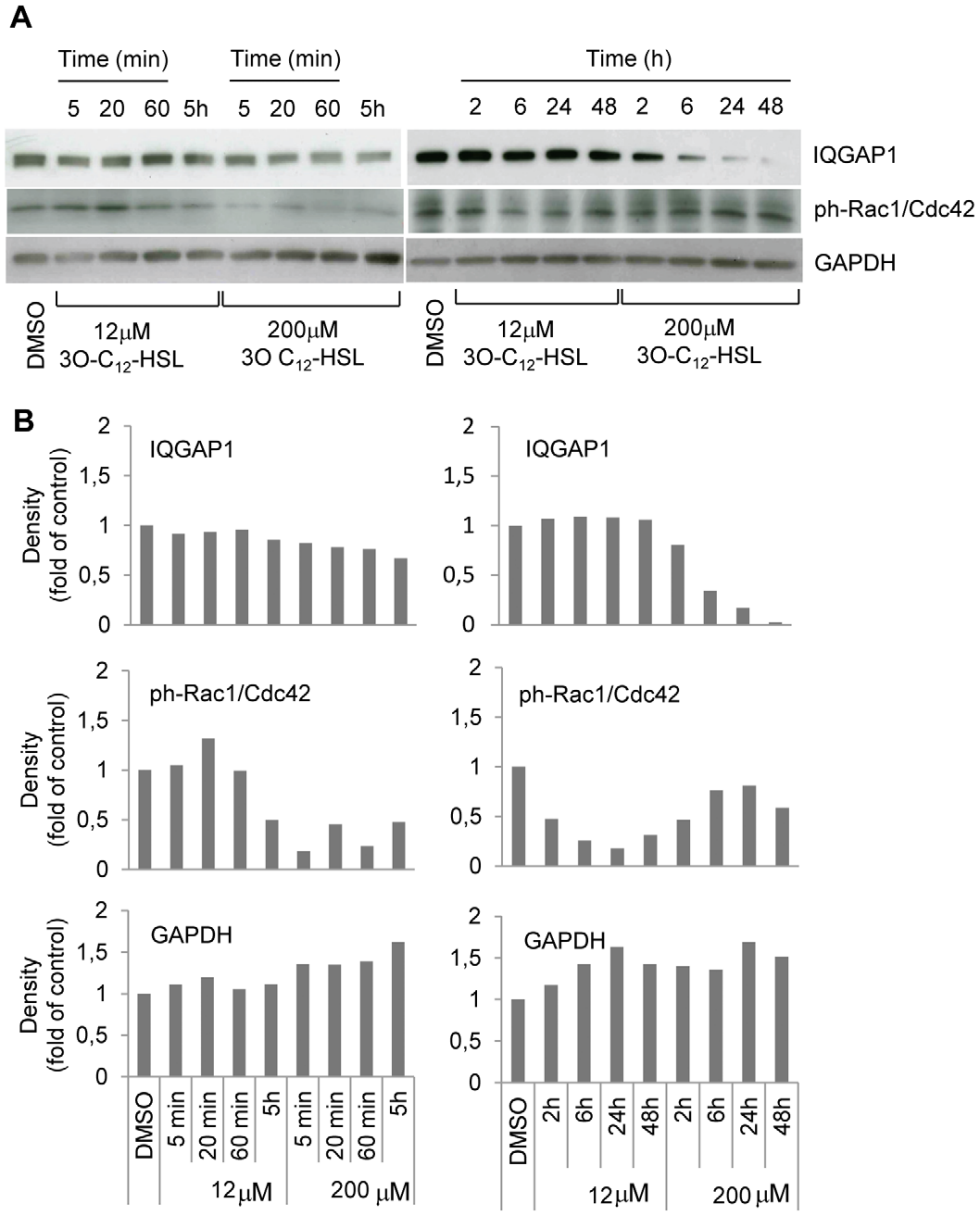
3O-C_12_-HSL modulates phosphorylation of Rac1/Cdc42 and level of IQGAP1. (A) Caco-2 cells were stimulated with 12, 200 µM 3O-C_12_-HSL or 0.018% DMSO as diluent control, for 5, 20, 60 min, or 2, 5, 6, 24, 48 h. Total cellular protein extracts were analyzed with Western blot using anti-IQGAP1, 189 kDa (upper panel), anti-phospho-Rac1/Cdc42, 28–25 kDa (middle panel) and anti-GAPDH, 36 kDa for loading control (lower panel). (B) Densitometric analysis. The data are from one representative of at least three independent experiments. Density of bands was normalized against DMSO treated cells; values are median.

The Rho-family GTPases, Rac1 and Cdc42 are upstream effectors of filamentous actin remodeling, and thereby regulators of cell shape and motility in mammalian cells. In neutrophils, 3O-C_12_-HSL was shown to modulate the phosphorylation status of Rac1 and Cdc42 on Ser71 [12]. Moreover, the RasGAP homology domain in IQGAP1 is known to directly interact with the Rho-family GTPases, Rac1 and Cdc42 in their phosphorylated and GTP-bound state [25]. Furthermore, it stabilizes Rac1 and Cdc42 in its activated form [26]. Based on this, together with the effect of 3O-C_12_-HSL on cell migration, we hypothesized that the phosphorylation status of Rac1 and Cdc42 in Caco-2 cells is altered on 3O-C_12_-HSL stimulation. This was tested by immunoblotting (Figure 5A) with subsequent quantification of the density ratios of the specific bands from different blots (Figure 5B). Here, the treatment of Caco-2 cells with 200 µM 3O-C_12_-HSL resulted in a strong and rapid drop in the phosphorylation of Rac1/Cdc42, which occurred within 5 min. It remained decreased over a long time and then again became similar to what we observed in DMSO- treated cells. In contrast, 12 µM 3O-C_12_-HSL within 5 min first led to a mildly enhanced phosphorylation of Rac1/Cdc42; longer incubation failed to phosphorylate the Rho GTPases. The level of IQGAP1 decreased gradually after 200 µM 3O-C_12_-HSL treatment from 5 min to 48 h (Figure 5A and B). This is in contrast to 12 µM 3O-C_12_-HSL which caused no changes in the expression level of IQGAP1 and was thus similar to the controls, DMSO-treated and untreated cells (not shown on Figure 5). Thus, it appears that 12 µM 3O-C_12_-HSL over shorter time spans initiates phosphorylation of Rac1/Cdc42, but leaves the levels of IQGAP1 unaffected, whereas 200 µM 3O-C_12_-HSL rapidly decreases the levels of both proteins.

**Figure 6:**
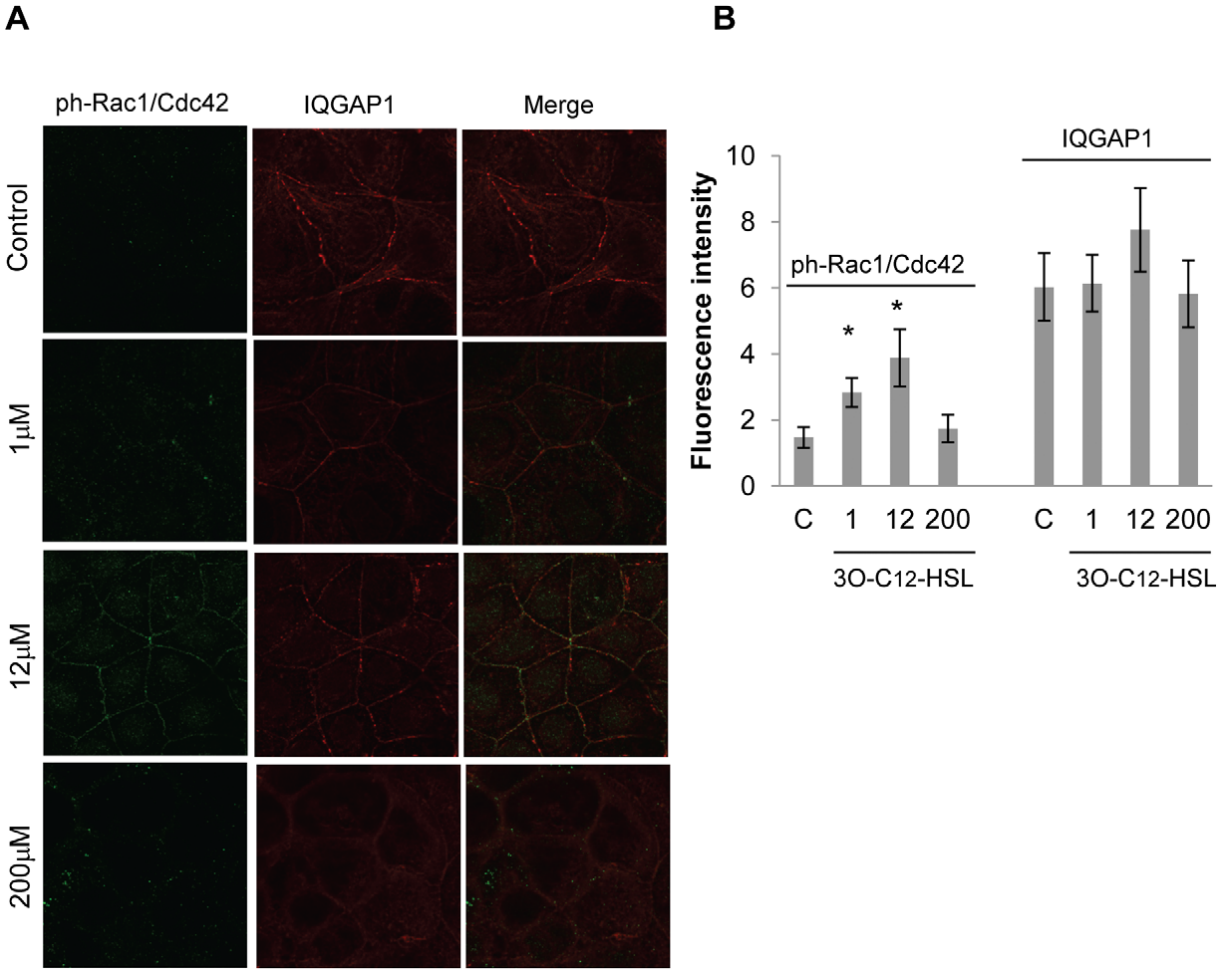
Confocal imaging of IQGAP1 and phosphorylated Rac1/Cdc42 in 3O-C_12_-HSL-treated Caco-2 cells. (A) Cell monolayers were stimulated with 1, 12, 200 µM 3O-C_12_-HSL or 0.018% DMSO as diluent control, for 20 min. Cells were fixed and stained with antibodies against IQGAP1 (red) and phospho-Rac1/Cdc42 (green) and analyzed by confocal laser scanning microscopy. The data are from one representative of at least three independent experiments. Image size is 67.6×67.6 µm and pixel size is 0.13 µm. (B) Quantification of immunofluorescence intensity of IQGAP1 and phospho-Rac1/Cdc42 staining. Columns represent means ± standard error (n = 10). The data are from at least three independent experiments. Significant differences (*) in mean for fluorescence intensity compared with values for control groups as calculated by Student's *t* test.

**Figure 7:**
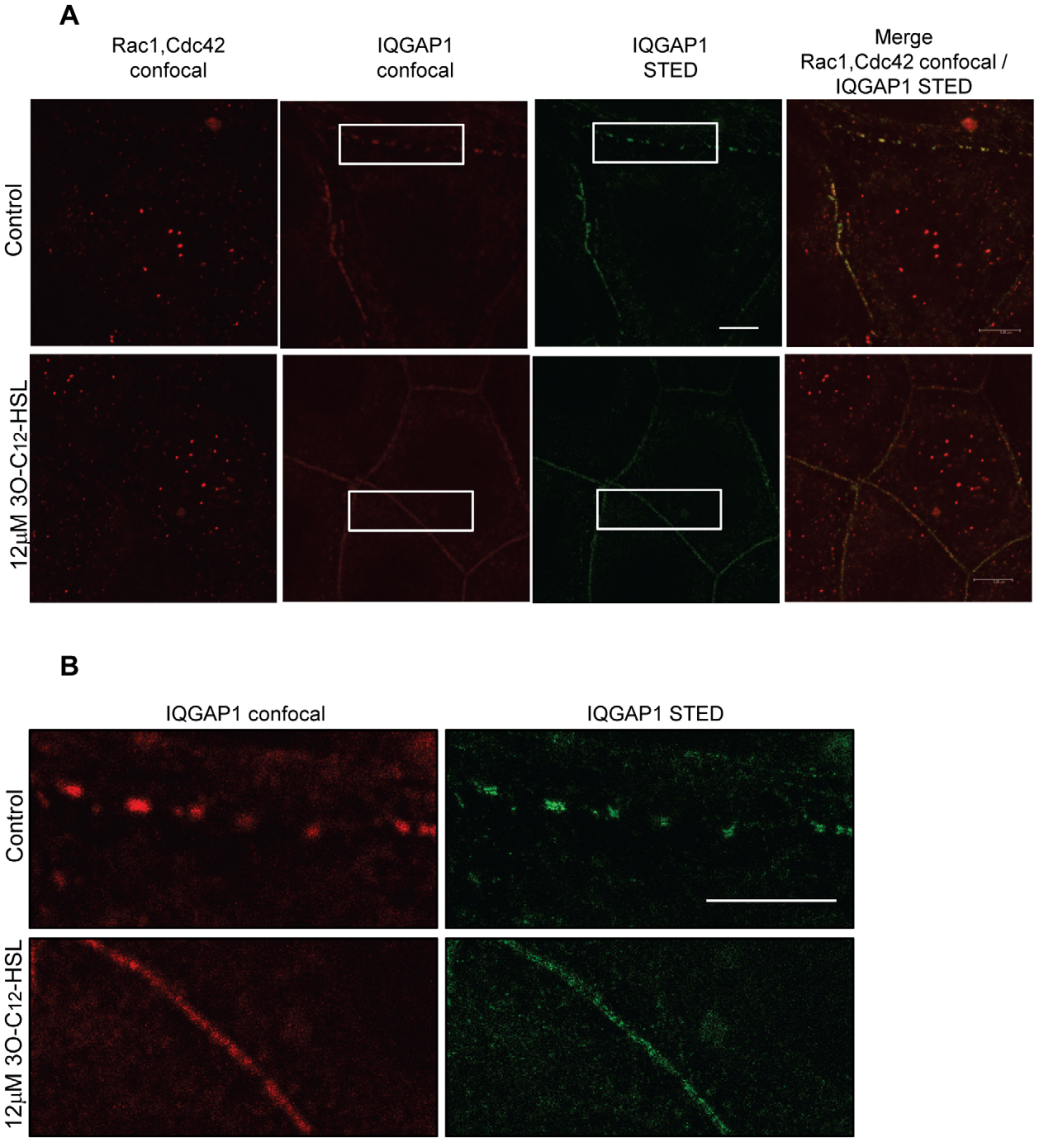
High resolution STED microscopy of IQGAP1 and phosphorylated Rac1/Cdc42 in 3O-C_12_-HSL-treated Caco-2 cells. Cell monolayers were stimulated with 12 µM 3O-C_12_-HSL or 0.018% DMSO as diluent control, for 20 min. Cells were fixed and stained with mouse anti-IQGAP1 and Atto 647N goat anti-mouse antibodies (green) and rabbit anti-phospho-Rac1/Cdc42 and Abberior Star 470SX goat anti-rabbit antibodies (red) and analyzed by confocal and STED microscopy. The data are from one representative of at least two independent experiments. (A) Main panels, bars: 5 µm. (B) Inserts, bar: 5 µm.

During locomotion, the cellular distribution and relocalization of motility-related proteins are of great importance. To assess the distribution of IQGAP1 and phosphorylated Rho GTPases Rac1 and Cdc42 and to further confirm a change in the expression levels, we used immunofluorescence confocal imaging (Figure 6A) and super-resolution STED microscopy (Figure 7). After stimulation with 1 and 12 µM 3O-C_12_-HSL for 20 min, we found that phosphorylated Rac1/Cdc42 proteins relocalized to the membrane region near IQGAP1 in comparison to control cells, which were treated with DMSO as a diluent control. Here, the cells displayed low levels of phosphorylated Rac1/Cdc42 in the cytoplasmic region, with minor staining in the nuclear area (Figure 6A and 7). Furthermore, the distribution of IQGAP1 went from membrane-related puncta to a more pronounced uniform membranous distribution after treatment with 12 µM 3O-C_12_-HSL. These changes were not apparent in cells treated with 200 µM 3O-C_12_-HSL (Figure 6A and 7). When measuring the changes in fluorescent intensity, we found that the expression of phosphorylated Rac1/Cdc42 increased 20 min after treatment with 1 and 12 µM 3O-C_12_-HSL whereas no alteration was detected after treatment with 200 µM 3O-C_12_-HSL (Figure 6B). The fluorescent intensity of IQGAP1 also remained fairly constant regardless of 3O-C_12_-HSL concentration (Figure 6B). Taken together, these findings indicate that 3O-C_12-_HSL can modulate the distribution of IQGAP1 and phosphorylation of Rac1 and Cdc42, the upstream effectors of filamentous actin remodeling.

### 3O-C_12_-HSL partly co-localizes with IQGAP1 but not with actin

**Figure 8:**
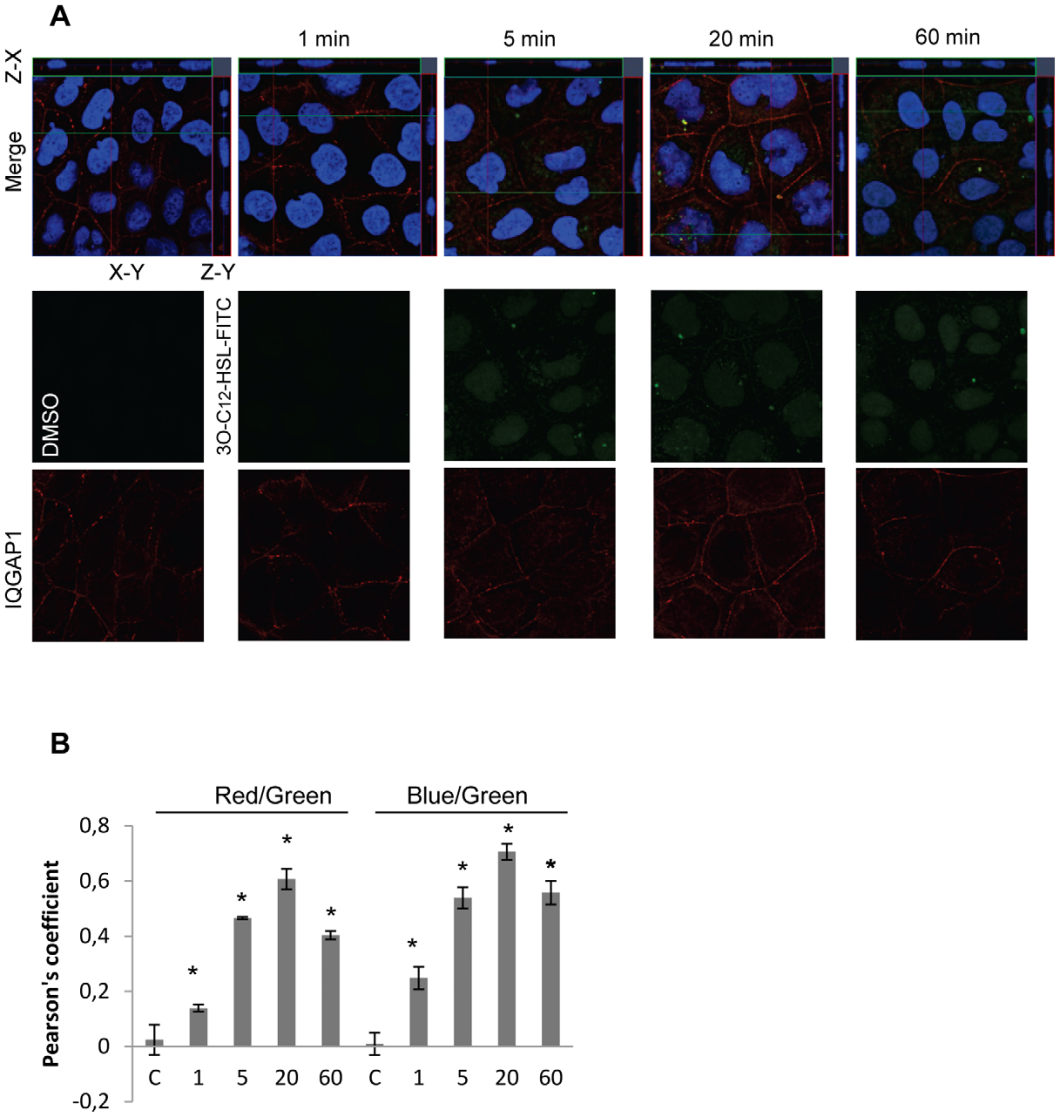
Visualization of 3O-C_12_-HSL-FITC and IQGAP1 in Caco-2 cells. (A) Caco-2 cell monolayers were treated with 1 µM 3O-C_12_-HSL-FITC (green) or 0.018% DMSO as diluent control for 1, 5, 20 or 60 min. Cells were fixed and stained with antibodies against IQGAP1 and Atto 647N goat anti-mouse antibodies (red) and DAPI nucleic acid stain (blue), and were analyzed by confocal laser scanning microscopy, showing an X-Y section (large insert), Z-X section (top) and Z-Y (right). The images are from one representative of at least three independent experiments. Image size is 67.6×67.6 µm and pixel size is 0.13 µm. (B) Measurement of co-localization, based on Pearson's coefficient. Columns show the mean ± standard errors (n = 10) based on three independent experiments. Significant differences (*) in mean for Pearson's coefficient compared with values for control groups as calculated by Student's *t* test.

**Figure 9:**
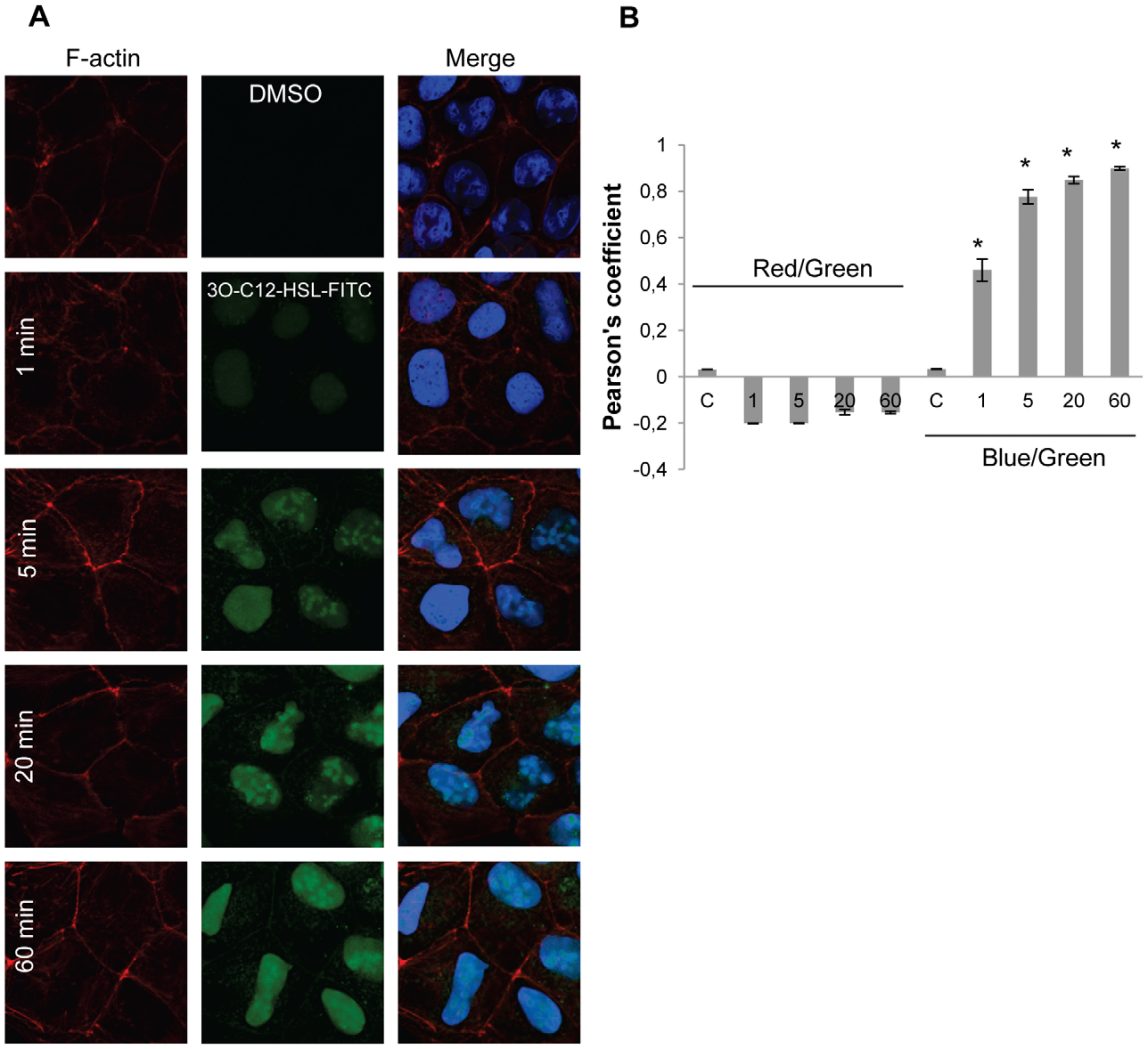
Visualization of 3O-C_12_-HSL-FITC and F-actin in Caco-2 cells. (A) Caco-2 cell monolayers were treated with 1 µM C_12_-HSL-FITC (green) or 0.018% DMSO as diluent control for 1, 5, 20, or 60 min. Cells were fixed and stained with Alexa Fluor 594-conjugated phalloidin to detect F-actin (red) and DAPI nucleic acid stain (blue), and were analyzed by confocal laser scanning microscopy. The images are from one representative of at least three independent experiments. Image size is 67.6×67.6 µm and pixel size is 0.13 µm. (B) Measurement of co-localization, based on Pearson's coefficient. Columns show the mean ± standard errors (n = 10) based on three independent experiments. Significant differences (*) in mean for Pearson's coefficient compared with values for control groups as calculated by Student's *t* test.

Because of the detected interaction of 3O-C_12_-HSL and IQGAP1, we wanted to visualize this interaction as well as the cellular distribution of 3O-C_12_-HSL. Furthermore, the impact of 3O-C_12_-HSL on cell migration also indicated an interaction with the cytoskeletal actin network. Therefore, we treated Caco-2 monolayers with 1 µM 3O-C_12_-HSL-FITC and immunostained for IQGAP1 (Figure 8A) and for F-actin with palloidin-Alexa594 (Figure 9A), followed by confocal imaging and subcellular co-localization analysis (Figures 8B and 9B). In the control cells, which were treated with diluents as a control, IQGAP1 was localized near the membrane as a dashed line and in cytoplasm both at the apical and lateral sides of membrane (Figure 8A). After a 1-, 5-, 20- and 60- min treatment with 1 µM 3O-C_12_-HSL-FITC, Caco-2 cells displayed gradually increased staining of 3O-C_12_-HSL located near the plasma membrane, in the cytoplasm and in the nucleus. In parallel, in 3O-C_12_-HSL-FITC-treated cells the IQGAP1 was seen as a more unbroken line alone the cell membrane and relocalized to the apical side of the plasma membrane compared with control cells (Figure 8A, Z-X and Z-Y images). Visible co-localization between 3O-C_12_-HSL and IQGAP1 was detected in the cells already after 5-, 20- and 60-min treatment with 1 µM 3O-C_12_-HSL-FITC. Its significance was confirmed by co-localization analysis using the Image J plug-in JACoP [27]. To quantify co-localization, correlation analysis based on van Steensel's approach (not shown by figure) and Pearson's coefficient (PC) was used. PC value usually ranges from 1 to −1, with 1 standing for complete positive correlation, and between zero and −1 for negative or no correlation. Figure 8B shows that PC for green (3O-C_12_-HSL) and red images (IQGAP1) in control cells approached 0, and in the case of 20-min treatment with 1 µM 3O-C_12_-HSL-FITC, a PC value of 0.6. Thus, we can state that a significant positive, partial and time-dependent co-localization between 3O-C_12_-HSL and IQGAP1 was observed. Between F-actin and 3O-C_12_-HSL, co-localization was not confirmed by correlation analysis based on van Steensel's approach (not shown by figure) and the PC (Figure 9B). Here, PC for green (3O-C_12_-HSL) and red images (F-actin) in control cells approached 0, and −0.2 in the case of treatment with 1 µM 3O-C_12_-HSL-FITC. PC for blue (nuclei) and green (3O-C_12_-HSL) images reached a value between 0 and 0.8, standing for partial co-localization between 3O-C_12_-HSL and nuclei (Figures 8B and 9B). Thus, 3O-C_12_-HSL primarily co-localizes with IQGAP1, also in the nucleus, but not directly with actin.

## Discussion

*Pseudomonas aeruginosa* is an environmentally highly adaptable human pathogen and a common cause of lung, ocular, skin, urinary tract and gastrointestinal tract infections. These bacteria can survive in almost every part of the intestine and often even replace the normal resident flora in critically ill and immuno-compromised patients [28]. Using the human epithelial colorectal adenocarcinoma Caco-2 cell line, it has been demonstrated that clinical isolates of *P.aeruginosa* have the ability to adhere to, penetrate and disrupt the barrier function and to form biofilms [29], [30]. From the intestine, *P.aeruginosa* can translocate to other organs and tissues by lymphatic and hematogenous dissemination and cause sepsis or lung infections. Moreover, it can promote sepsis by creating a leaky barrier to different toxins [31]. *P.aeruginosa* biofilms are known to exist in wounds and disrupted epithelial barriers, and it is expected that their presence may delay wound healing, especially in chronic wounds [32], [33]. QS signaling among the bacteria, once the biofilm reaches a critical density, additionally promotes virulence and may also affect wound healing.

In this study we focused on whether, and how, the migration of epithelial cells was affected by *P.aeruginosa* 3O-C_12_-HSL. Using human epithelial Caco-2 cells in Ibidi migration assays, we clearly showed that 3O-C_12_-HSL-treatment modulates cell motility in a dose- and time-dependent manner. When compared with controls, 100 and 200 µM 3O-C_12_-HSL induced significant suppression of migration in Caco-2 cells. On the contrary, cells treated with lower concentrations, i.e. 1.5, 3, and 12 µM 3O-C_12_-HSL, displayed significantly promoted penetration into the wound. Our findings corroborate the earlier observations that 10 µM 3O-C_12_-HSL increases the wound contraction. The study [34] used full-thickness cutaneous wound-healing in rats and applied low doses of 3O-C_12_-HSL to the granulation tissue. In this animal model, epithelial cells, inflammatory cells and fibroblasts might have played a combined role in the wound- healing process.

The inhibitory effect of 100 and 200 µM 3O-C_12_-HSL on Caco-2 cell migration raised the question of whether 3O-C_12_-HSL might suppress cell proliferation and viability. It appears that 3O-C_12_-HSL can inhibit cell proliferation and induce apoptosis in certain cell types, including cystic fibrosis airway epithelial cells [35], T-cells [36], [37], breast carcinoma cells [38], murine neutrophils and monocytic cells [39] and fibroblasts [11]. Apoptosis in these cells was confirmed by changes in cell morphology and activation of caspases. Indeed, this effect appears to be cell-type specific as in epithelial Caco-2, cell proliferation and viability were not changed by 3O-C_12_-HSL in the range of 6–200 µM, as shown in this study. Our findings are in line with observations by other groups, which compared the effect of 3O-C_12_-HSL on non-tumorigenic breast epithelial cells [38] and CCL-185 and HEp-2 epithelial cells [39]. The studies clearly demonstrated that these epithelial cells were tolerant to 3O-C_12_-HSL-induced apoptosis.

Taken together, our results provide new evidence that *P.aeruginosa* 3O-C_12_-HSL plays at least two distinct roles. Besides regulating the expression of virulence factors and biofilm formation in bacteria, it also plays a crucial role in the regulation of human epithelial cell migration. This probably allows *P.aeruginosa* to maintain long-term systemic infections at later stages in the host.

*P.aeruginosa* QS molecules are known to exist in bacterial biofilms. Thus, 3O-C_12_-HSL and C_4_-HSL have been detected in the sputum from bacteria-colonized cystic fibrosis patients at 1–22 nM and 1–5 nM, respectively [40]. These levels of signal molecules were surprisingly low, but could be explained by dilution in, the efficiency of extraction from samples, heterogeneity of biofilms growing in the lung and the choice of methods for quantifying of QS molecules [40]. However, notably higher 3O-C_12_-HSL concentrations, 0.5–1.41 µM, were recently detected using LC-MS/MS when *P.aeruginosa* was growing in media *in vitro* [41]. Charlton et al. have also shown that QS molecules can accumulate at very high levels in biofilms grown *in vitro*, yielding up to 300–600 µM 3O-C_12_-HSL concentrations [42].

In addition, increasing evidence suggests that AHLs can induce phenotype changes in neighbors, including other bacteria and eukaryotic host cells. Therefore, identifying targets for AHL may allow better understanding of this communication. Recently, Meijler et al. [43] designed and validated a diazirine-based 3O-C_12_-HSL probe, which they used in their attempts to isolate and identify putative receptor(s) in eukaryotic cells that target 3O-C_12_-HSL. In related work, Janda et al. [44] synthesized alkynyl- and azido-tagged probes, which could also be utilized to detect the mammalian protein target of 3O-C_12_-HSL. Inspired by these recent reports, Blackwell et al. [45] have designed and synthesized a new affinity matrix and demonstrated that it can bind to QscR, the native bacterial receptor for 3O-C_12_-HSL.

We also focused our investigations on the mechanisms whereby 3O-C_12_-HSL induces changes in epithelial Caco-2 cell migration. To address this issue we used and validated two new affinity probes, *N*-dodecanoyl-L-homoserine lactone-3-hydrazone-biotin (3O-C_12_-HSL-3H-biotin) and *N*-dodecanoyl-L-homoserine lactone-biotin (3O-C_12_-HSL-biotin). We believed that this would allow us to capture 3O-C_12_-HSL-protein complexes through their interaction with streptavidin-agarose resin and to identify proteins by LC-MS/MS. Through this approach, the biochemical experiments provided strong evidence that 3O-C_12_-HSL interacts with IQGAP1. The affinity of 3O-C_12_-HSL for IQGAP1 in cytoplasmic fraction of Caco-2 cells was successfully observed with the 3O-C_12_-HSL-3H-biotin probe. The other probe, 3O-C_12_-HSL-biotin, failed to bind to IQGAP1 and the protein was not identified by LC-MS/MS. This data could be explained by the fact that 3O-C_12_-HSL molecules with a modified AHL lactone ring yield less potent QS signals and are probably less prone to interact with its target molecules. Our data are in line with a recent report [6] showing that only 3O-C_12_-HSL molecules with an intact AHL lactone ring and a long acyl chain were capable of directly interacting with phospholipids in an artificial plasma membrane systems and Jurkat T-cell membranes. Our findings also corroborate earlier observations [23] that AHL structures lacking the L-configuration and the lactone ring were devoid of the ability to inhibit mouse and human leukocyte proliferation and TNF-α secretion, and to act as immune modulators.

IQGAP1 is a 189 kDa protein that contains multiple domains for binding other proteins and localizes in the leading edge of migrating cells, usually as an 380 kDa homodimer [25], [46], [47] The RasGAP homology domain in IQGAP1 directly interacts with the Rho-family GTPases, Rac1 and Cdc42 in their GTP-bound state [25] and stabilizes Cdc42 in its GTP-bound state [26]. IQGAP1 plays an essential role in such aspects of cell physiology as cell shape, vesicle trafficking, polarization, adhesion and directional migration [47]. It likely mediates these processes through its many other protein-interacting domains, which can directly link it to actin, myosin light chain, β-catenin, E-cadherin, calmodulin, CLIP-170, mitogen-activated protein kinase and the extracellular signal-related kinases [48], [49]. This allows IQGAP1 to function as a true scaffolding protein. Several studies have indicated that IQGAP1 is also an essential regulator of the receptor protein-tyrosine phosphatase [50], the epidermal growth factor receptor, a member of the receptor tyrosine kinase family [51], and the chemokine receptor CXCR2 [52].

**Figure 10:**
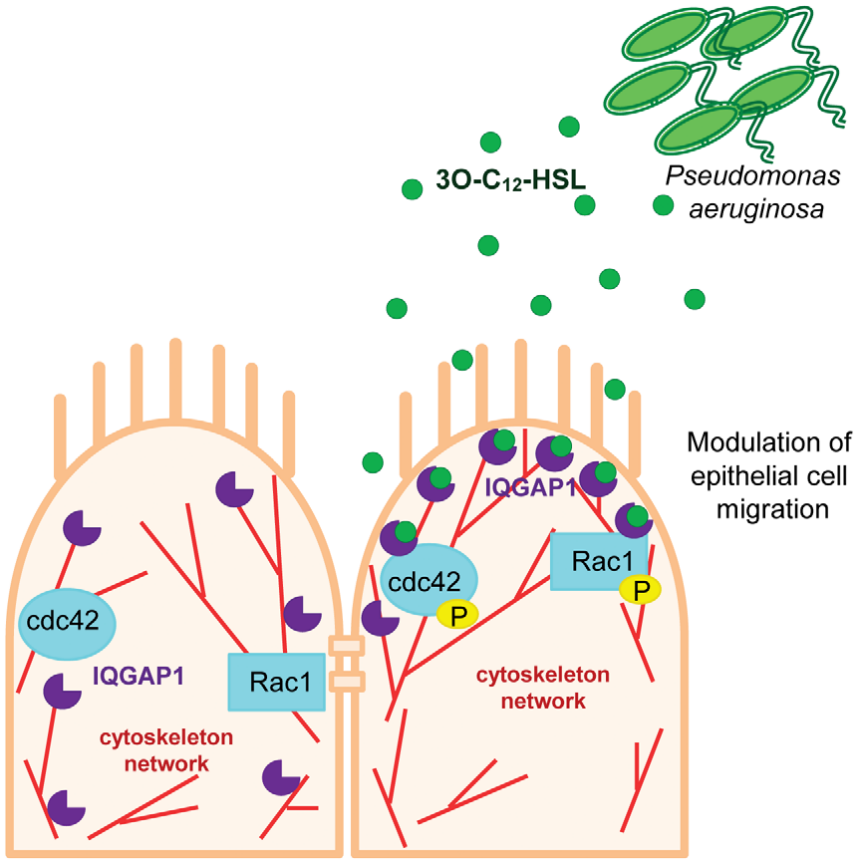
Model of the communication between *P.aeruginosa* 3O-C_12_-HSL and human epithelial Caco-2 cells. *P.aeruginosa* 3O-C_12_-HSL interacts and co-localizes with IQGAP1. The targeting of IQGAP1 by 3O-C_12_-HSL initiates early event of communication between Caco-2 cells and bacteria via 3O-C_12_-HSL and can further trigger the essential changes in the cytoskeleton network of epithelial cells. Also, 3O-C_12_-HSL modulates Caco-2 cell migration in a dose- and time-dependent manner. It also alters the phosphorylation status of Rac1 and Cdc42, and cellular distribution and localization of IQGAP1 from the basolateral to apical side of epithelial cells.

Why should 3O-C_12_-HSL associate with IQGAP1? If the actin cytoskeleton is exploited by bacteria, it can help them enter into host cells or move within cells [53]. By altering the dynamics between the plasma membrane and the actin cytoskeleton, it can locally disrupt the cytoskeleton, subvert membrane-associated signaling pathways and promote further successful invasion and infection [54]. We propose that the interaction between IQGAP1 and *P.aeruginosa* 3O-C_12_-HSL triggers such essential changes in the cytoskeleton network to initiate very early events of bacterial-mammalian cell communication. Consistent with this scenario, *P.aeruginosa* relies on alterations of membrane properties at the leading edge (PI3K, Rac1, IQGAP1 and actin) for the insertion and function of type III secretion translocon and to establish an infection [55] (Figure 10).

To confirm LS-MS/MS results on the 3O-C_12_-HSL affinity for IQGAP1, we employed a GST-tagged full-length IQGAP1 [24] pull-down assay and probed to catch an interacting target, 3O-C_12_-HSL, with subsequent Western blot detection of IQGAP1 and identification of 3O-C_12_-HSL in a sensor bioassay. Indeed, 3O-C_12_-HSL appeared to bind to IQGAP1 but not to actinin 4 (Figure 4).

To further examine the association of 3O-C_12_-HSL with IQGAP1 we used a new fluorescently-tagged probe, *N*-dodecanoyl-L-homoserine lactone-3-hydrazone-fluorescein (3O-C_12_-HSL-FITC), which allows the 3O-C_12_-HSL molecule to be assessed using confocal imaging and subsequent subcellular co-localization analysis. We found that 3O-C_12_-HSL entered into Caco-2 cells and co-localized with IQGAP1, but not directly with actin (Figures 8 and 9). Besides recognizing IQGAP1, 3O-C_12_-HSL was found in the nucleus, which can be explained by recent findings that 3O-C_12_-HSL may utilize nuclear peroxisome proliferator-activated receptors (PPAR) to regulate the transcriptional activity and NF-κB signaling [9], [10]. We think that the interactions with the membrane [6], diffusion and entering into the cytoplasm [7] as well as targeting of IQGAP1 (this study) and binding to nuclear receptors [9], [10] do not exclude each other. It has, for example, been shown that different types of lipids, such as leukotriene B4, can bind to both cell-surface LTB4 receptor and nuclear PPAR ligands [56] to mediate pro-inflammatory events, such as chemotaxis and chemokinesis.

The interaction of IQGAP1 with various pathogen-derived partners has recently been reviewed [57]. For instance, *Salmonella typhimurium* engaged IQGAP1 to enter host cells through interaction with actin, Rac1 and Cdc42 [58]. Indeed, the effector protein, Ssel, has been shown to translocate into host cells using the type III secretion system (TTSS) and inhibit migration of macrophages and dendritic cells *in vitro* to maintain a long-term systemic infection in mice [59]. Another pathogen, *Escherichia coli*, uses a TTSS-dependent effector protein, termed Ibe, which binds to, and co-localizes with IQGAP1 in bacteria-induced pedestals and actin-rich membrane ruffles [60]. An IQGAP1 interaction may also be utilized by retroviruses. Accordingly, murine IQGAP1 interacted with matrix protein of Moloney murine leukemia virus, which correlated with virus replication [61].

As Caco-2 cell migration was changed by the 3O-C_12_-HSL challenge, putatively using IQGAP1 as a target, we hypothesized that the phosphorylation status of Rho GTPases Rac1 and Cdc42 could also be affected. Our data clearly show that 3O-C_12_-HSL modulated the cellular localization of IQGAP1 and phosphorylation status of Rac1 and Cdc42, the upstream effectors of filamentous actin remodeling, cell shape change and motility (Figures 6 and 7). It has been shown that by interacting with a cognate receptor for fibroblast growth factor, IQGAP1 can regulate the polarized distribution of receptors at the cell surface and modulate cell motility by actin assembly [47]. These results are in agreement with the 3O-C_12_-HSL-mediated changes in Caco-2 cell migration (Figures 2 and S2), reduced expression of key tight- and adherens-junction proteins [62], [63], [64], and with the general concept that the actin cytoskeleton is essential for the structure and function of epithelial cells. Various bacterial effectors and toxins target GTPases and the actin cytoskeleton either directly by modifying actin or by inhibiting actin polymerization; new findings about these bacterial products have been discussed in detail in recent reviews [65], [66].

To conclude, in intestinal epithelial Caco-2 cells, the *P.aeruginosa* QS molecule 3O-C_12_-HSL: (1) modulated cell migration in a dose- and time-dependent manner without affecting proliferation and viability; (2) interacted and co-localized with IQGAP1; (3) altered the phosphorylation status of Rac1 and Cdc42, and cellular localization of IQGAP. Collectively, our study suggests that IQGAP1 is a novel target for 3O-C_12_-HSL and thereby likely interferes with Rac1 and Cdc42- dependent reorganization of actin cytoskeleton and altered cell migration (Figure 10). We propose that the targeting of IQGAP1 by *P.aeruginosa* 3O-C_12_-HSL triggers essential changes in the cytoskeleton network to initiate very early events of communication between bacteria and human epithelial cells.

## Materials and Methods

### Cell culture

Human epithelial colorectal adenocarcinoma Caco-2 cells (passages 84–95) were grown in Dulbecco's modified Eagle's medium (DMEM) supplemented with 10% heat-inactivated fetal calf serum, 100 U/ml penicillin, 100 µg/ml streptomycin, 1% non-essential amino acids and 2 mM L-glutamine (Gibco Invitrogen Corporation, UK) at 37°C in 5% CO_2_. Cells were passaged weekly upon reaching 80% confluence.

### AHL synthesis and validation

*N*-3-oxo-dodecanoyl-L-homoserine lactone C_16_H_27_NO_4_, MW 297.4 (3O-C_12_-HSL) was synthesized by Prof. Peter Konradsson and Lan Bui (Dept. of Organic Chemistry, University of Linköping, Sweden) as previously described [23] (Figure 1A). These molecules are structurally and functionally identical to those obtained from *P. aeruginosa* cultures. The resulting 3O-C_12_-HSL was checked for identity and purity by HPLC, and its activity as a quorum-sensing molecule was confirmed by the bioassays described earlier [67], [68]. 3O-C_12_-HSL was dissolved in 100% dimethylsulfoxide (DMSO) as a stock solution for the experiments and then diluted with the aqueous buffer of choice. The fluorescently-tagged probe, *N*-dodecanoyl-L-homoserine lactone-3-hydrazone-fluorescein, C_37_H_40_N_4_O_8_S, MW 700.8 (3O-C_12_-HSL-FITC) and two biotin-conjugated probes, *N*-dodecanoyl-L-homoserine lactone-3-hydrazone-biotin C_26_H_43_N_5_O_5_S, MW 537.7 (3O-C_12_-HSL-3H-biotin) and *N*-dodecanoyl-L-homoserine lactone-biotin C_38_H_41_N_3_O_9_S, MW 552.7 (3O-C_12_-HSL-biotin) were obtained by request from Cayman Chemical (Ann Arbor, MI) (Figure 1B–D). The biological activity of these conjugates was validated using the lux-based *E.coli* JM109 pSB1075 sensor assay described earlier [67] (Figure S1A) and by their ability to disrupt tight junction protein ZO-3 in Caco-2 cells [62] (Figure S1B). Based on AHL sensor assay, the biological activity of 3O-C_12_-HSL-3H-biotin remained at approximately 100%, of 3O-C_12_-HSL-biotin at 65% and of 3O-C_12_-HSL-FITC at 85%.

### Migration assays

Caco-2 cells were seeded in μ-dishes with inserts (Ibidi GmbM, Martinsried, Germany) and cultured until monolayers were 70–80% confluent. Then, cells were serum-starved overnight and the insert was removed to get two cell patches with a 500±50 µm cell-free gap in between. Cells were treated with 0.018% DMSO (diluent control), or 1.5, 6, 12, 25, 50, 100 and 200 µM 3O-C_12_-HSL. For each dish, four images of cells migrating into the gap area were taken at 0, 24, 48 and 72 h using a ProgRes C10 Plus camera (Jenoptik, Jena, Germany) coupled to an inverted microscope (Olympus, Tokyo, Japan) equipped with a 10× long working distance objective; between imaging, cells were returned to the incubator. Migration rates were calculated by measuring the distance between cell monolayer patches, using six measurements per image and four images per dish for the each time point with the Image J software (NIH). At least six independent experiments were performed on separate days on different cell passages. In addition, a modified migration assay was used, in which the cells were cultured to form monolayers on tissue culture-treated, rat tail collagen-, or human fibronectin-coated 96-well plates with a cylinder-like plug in each well (Oris Platypus Technologies, Madson, WI). Thereby, a circular, 2 mm diameter wound was created by removing the insert. Here, cells were treated with 0.018% DMSO (diluent control), or 12 and 200 µM 3O-C_12_-HSL. For each well, one image was taken with the same camera coupled to an inverted microscope as above with a 1.25× long working distance objective at 0, 24, 48 and 72 h. Independent experiments were performed three times in eight identical wells on separate days on different cell passages. Migration was assessed by measuring the diameter of the wounds, using three measurements per image for the each well and each time point with the Image J software (NIH).

### Preparation of cell lysates and protein fractions

Cells were cultured until monolayers were 70–80% confluent. To prepare total-cell lysate, cells were rinsed with PBS, pH 7.6. and lysed with ice-cold RIPA buffer (1% NP-40, 1% deoxycholic acid sodium salt, 0.1% SDS, 150 mM NaCl, 10 mM Tris pH 7.4, 10 mM EDTA pH 8.0 dissolved in PBS) containing benzonase (Novagene, Denmark), 1 mM phenyl-methyl-sulfonyl-fluoride, 1 mM Na_3_VaO_4_, 25 mM NaF (Sigma), protein inhibitors Complete (Roche Diagnostics, Mannheim, Germany). Cell suspensions were homogenized through a 21-gauge needle and centrifuged at 18,000 g for 30 min at 4°C, and the supernatants were collected. To obtain separate fractions of cytoplasmic and membrane proteins, the ProteoJET membrane protein extraction kit (Fermentas Thermo Scientific, Vilnius, Lithuania) was used. The protein concentration in cell lysates was measured with the Bio-Rad D_C_ protein assay (Bio-Rad Laboratories).

### Precipitation with biotinylated AHLs

In a tube, 1 mL of whole-cell lysate, cytoplasmic or membrane fraction was incubated with no additions, 0.05 mg 3O-C_12_-HSL, 4 µg biotin (as controls), 0.05 mg 3O-C_12_-HSL-3H-biotin or 3O-C_12_-HSL-biotin overnight at 4°C. Complexes were captured at 4°C for 1 h using Streptavidin agarose resin (Pierce Biotechnology, Rockford, IL), according to the manufacturer's instructions. The resin-bound complexes were collected by centrifugation for 1–2 min at 2,500 g and washed four times with cold PBS, pH 7.6. The supernatant which contains resin-unbound components was saved and further used as a control. The resin-bound complexes and resin-unbound components were resuspended in Laemmli sample buffer at equal protein concentrations, boiled for 5 min at 95°C loaded on 4–12% SDS-polyacrylamide gels (Lonza, Rockland, ME). After electrophoresis, the gels were stained with PageBlue protein staining solution (Fermentas Thermo Scientific) and followed with in-gel digestion and LC-MC/MC analysis.

### Protein identification and peptide analyses by LC-MS/MS

For in-gel digestion, the protein bands were excised, destained with acetonitrile, reduced by DTT (10 mM in 100 mM ammonium bicarbonate, 45 min, 56°C) and alkylated by iodoacetamide (55 mM in 100 mM ammonium bicarbonate, 30 min, 25°C, in the dark). The proteins were digested for 24 h at 37°C in 25 mM ammonium bicarbonate with sequencing grade modified trypsin (Promega, Madison, VI) essentially according to the procedure described by Shevchenko *et al.* [69], [70]. The peptide mixtures obtained were analyzed by LC-MS/MS, using nano-flow HPLC system (EASY-nLC from Bruker Daltonics, Bremen, Germany) on a 20 mm×100 µm (particle size 5 µm) C18 pre-column followed by a 100 mm×75 µm C18 column (particle size 5 µm) at a flow rate 300 nL/min, using a linear gradient constructed from 0.1% formic acid (solvent A) to 0.1% formic acid in 100% acetonitrile (solvent B): 0–100% B for 45 min. Data were acquired using an on-line electrospray ionization ion trap “HCTultra PTM Discovery System” (Bruker Daltonics, Bremen, Germany). The automated online tandem MS analyses were performed using collision-induced dissociation of peptide ions. Peak lists were created from the raw data using Bruker Daltonics DataAnalysis 3.4 (Bruker Daltonics, Bremen, Germany) and the resulting MGF files were used to search for *Homo sapiens* proteins in Swiss-Prot on the Mascot server (www.matrixscience.com). The search parameters allowed mass errors up to 0.6 Da for MS data, and up to 0.6 Da for MS/MS data. The charge states of the peptides were varied; three missed cleavage sites were permitted. Cysteine carbamidomethylation was selected as a fixed modification. N-terminal protein acetylation and methionine oxidation were selected as variable modifications. For identification of peptides we used the following criteria: the MASCOT score was above 24, the significance threshold was set at 0.05 and redundant identifications were excluded using the bold red function.

### Pull-down assay

The plasmid 2-GEX-2T-IQGAP1 was a gift from Dr. David Sacks' laboratory (Addgene plasmid #30107). The plasmid was transformed into BL21 Star One Shot *E.coli* (Invitrogen) and the protein expression was induced by the addition of 0.1 mM IPTG to the culturing medium when the bacteria had reached an optical density (OD_600_) of 0.4. Expression of full length GST-IQGAP1 was allowed for 3 h, after which the bacteria were pelleted and frozen at −20°C. The pellet was thawed on ice and the bacteria were lysed in PBS, containing 1 mM EDTA, 1% (v/v) Triton X-100, 1 mg/ml lyzosyme (all obtained from Sigma) with Protease inhibitor cocktail (Pierce Thermo Scientific). The lysates were allowed to rotate for 30 min at room temperature and undissolved debris was spun down at 15000 g for 25 min at 4°C.

1 ml of Glutathione HiCap Matrix (Qiagen) was added to disposable Talon columns (Clontech) that were subsequently equilibrated with 5 ml of PBS-based equilibration and wash buffer (PBS-EW) containing 1 mM DTT and 1 mM EDTA. They were then plugged, put on ice, and the cleared lysates were added. The samples were incubated for 20 min, and the plug was removed allowing unbound lysate to flow through. After this, the columns were washed twice in 2.5 ml equilibration buffer and subsequently plugged again. Then, 0.5 ml of the elution buffer (TNGT) containing 50 mM Tris pH 8.0, 0.4 M NaCl, 50 mM reduced Glutathione, 0.1% Triton X-100 and 1 mM DTT (all obtained from Sigma) was added to the columns and the samples were incubated for 15 min at RT after which the flow-through was collected. The elution step was repeated four times to obtain four different elution fractions. The protein concentration and purity was determined by gel-electrophoresis and Comassie staining together with known BSA standards. Detection of IQGAP1 and GST was further confirmed with Western blot. For the pull-down assay, 25 µl Glutathione HiCap Matrix (50% slurry) was equilibrated in five separate reaction tubes with 1 ml PBS-EW and mixed according to scheme: GST-IQGAP1 full-length and 3O-C_12_-HSL (final concentration 25 nM for both); GST-IQGAP1 full-length; 3O-C_12_-HSL; GST-actinin-4 full-length (Abnova, Taioei, Taiwan, #H00000081-P01) and 3O-C_12_-HSL (final concentration 25 nM for both); and without adding (Matrix alone). GST-tagged proteins and Matrix were incubated first alone overnight at 4°C with end-over-end mixing. After this, the 3O-C_12_-HSL was added as indicated in the above scheme and incubated for a further 1 h at 4°C with end-over-end mixing. Beads were washed in PBS-EW and centrifuged at 1000×g for 2 min at 4°C at least three times. The GST-fusion proteins, eventually with 3O-C_12_-HSL bound to them, were eluted from beads by adding 50 µl TNGT and incubated for 10 min. After centrifugation, the eluates were collected to analyze IQGAP1 and actinin 4 by SDS-PAGE and Western blot and to detect 3O-C_12_-HSL using *E.coli* JM109 pSB1075 lux reporter bioassay as described previously [67].

### Western blot analysis

Cells were cultured in a 6-well plate until monolayers were 70–80% confluent. Subsequently 0.018% DMSO, or 12 and 200 µM 3O-C_12_-HSL, were added for stimulation. After 5-, 20-, 60-min and 2, 5, 6, 24, 48 h incubation at 37°C, the cells were washed with PBS and lysed with ice-cold RIPA buffer as described above to obtain a whole-cell lysate. The protein concentration in the cell lysates was measured with the Bio-Rad D_C_ protein assay (Bio-Rad Laboratories). They were further diluted in Laemmli sample buffer at equal protein concentrations, heated for 5 min at 95°C and then subjected to SDS- polyacrylamide gel electrophoresis. The samples were loaded on 4–12% SDS-polyacrylamide gels (Lonza, Rockland, ME), and after separation, proteins were electrophoretically transferred to a PVDF membrane (Millipore, Bedford, MA); the quality of the transfer was monitored by Ponceau S staining. Non-specific binding was blocked by 1-h incubation at room temperature in 5% non-fat milk in PBS pH 7.6, containing 0.18% Tween 20. The membranes were then incubated with anti-phospho-Rac1/Cdc42 (Ser71) antibodies (Cell Signaling Technology Denvers, Boston, MA), anti-IQGAP1 antibodies, anti-GAPDH antibodies (Millipore, Temecula, CA), or anti-actinin4-antibodies (Sigma, #WH0000081M1) diluted 1∶1000 overnight at 4°C. After washing, they were further incubated with horseradish peroxidase (HRP)-conjugated secondary antibody (DAKO, Glostrup, Denmark) for 1 h at room temperature, washed and immunoreactive bands were visualized with Super Signal West Pico chemiluminescent substrate (Pierce), according to the manufacturer's instructions. The density ratio of the specific bands from different blots (X-ray film images) was quantified using the Image J software (NIH).

### Microscopy

Caco-2 monolayers, aged 7–10 days and grown on glass coverslips of thickness 0.17±0.01 and 13 mm-diameter (Karl Hecht Assistent, Sondheim, Germany), were exposed to 1, 12 and 200 µM 3O-C_12_-HSL or 0.018% DMSO as dilution control for 20 min at 37°C. In the experiments where we detected FITC-conjugated 3O-C_12_-HSL, Caco-2 monolayers were treated with 1 µM 3O-C_12_-HSL-FITC for 1, 5, 20 and 60 min at 37°C in the dark. The cells were washed with PBS, pH 7.3, treated with 0.05% Triton X-100 (Sigma) in PBS for 1 min, washed and fixed in 3% paraformaldehyde (Sigma) in PBS for 20 min at room temperature. The pre-treatment with 0.05% Triton X-100 was important for a clear labeling of proteins. After washing with PBS, cells were permeabilized in 0.2% Triton X-100 in PBS for 5 min and washed again. Non-specific background staining was blocked for 60 min with PBS containing 1% BSA and 10 mM glycine. The washing procedures were repeated and anti-phospho-Rac1/Cdc42 antibodies (Cell Signaling Technology) and anti-IQGAP1 antibodies (Millipore, Temecula, CA), diluted in blocking buffer according to the manufacturer's recommendations, were then applied overnight at 4°C in a moist chamber. After washing, Alexa Fluor 488-conjugated goat anti-rabbit antibodies (Molecular Probes Invitrogen) and Atto 647N goat anti-mouse antibodies (Active Motif, Carlsbad, CA) were added and incubated for 1 h at 24°C in the moist dark chamber. To detect F-actin, fixed and permeabilized cells were stained with Alexa Fluor 594-conjugated phalloidin (Molecular Probes Invitrogen), diluted 1∶40 in PBS from 200 units/ml methanol stock solution, for 45 min at 37°C in the moist dark chamber. In some experiments, nuclei were further stained with DAPI (Molecular Probes Invitrogen), according to the manufacturer's instructions. Finally, coverslips were washed in PBS and mounted on glass microscope slides in ProLong Gold antifade reagent (Molecular Probes Invitrogen). The specimens were examined through 63× oil immersion objectives with NA 1.40 in a fluorescence microscope Zeiss Axio Observer Z1 with confocal system Zeiss LSM700 and Zeiss ZEN software (Carl Zeiss, Jena, Germany). For high resolution microscopy (<70 nm), the specimens were examined in a Leica TCS STED Stimulated Emission Depletion confocal microscope with pulsed IR-laser and 100× oil immersion objective (Leica Microsystems, Mannheim, Germany). Fluorescence staining intensity was measured quantitatively using the Image J software (NIH). Subcellular co-localization was analyzed under Image J plug in JACoP [27].

### Statistical analysis

Where indicated, statistical analysis was performed by calculating means, standard deviations and standard errors; differences between groups were evaluated with the Student's *t*-test; *P*-values≤0.05 (*), ≤0.01 (**), ≤0.001 (***) were considered statistically significant.

## Supporting Information

Dataset S1 **Peptide identification views from MASCOT MS data analyses of IQGAP1 peptides sequenced by collision-induced dissociation of their ions.** The spectra and corresponding lists of fragment ions identified in the MASCOT search are shown.
(DOCX)

Dataset S2 **Peptide identification views from MASCOT MS data analyses of IQGAP2 peptides sequenced by collision-induced dissociation of their ions.** The spectra and corresponding lists of fragment ions identified in the MASCOT search are shown.
(DOCX)

Figure S1 **Biological activity of synthetic AHL molecules used in this study.** (A) Induction of luminescence in lux-based AHL biosensor reporter bacteria (*E.coli* JM109 pSB1075) by 10 ng of *N*-3-oxo-dodecanoyl-L-homoserine lactone C_16_H_27_NO_4_, (3O-C_12_-HSL), biotin-conjugated probe *N*-dodecanoyl-L-homoserine lactone-3-hydrazone-biotin C_26_H_43_N_5_O_5_S, (3O-C_12_-HSL-3H-biotin), *N*-dodecanoyl-L-homoserine lactone-biotin C_38_H_41_N_3_O_9_S, (3O-C_12_-HSL-biotin) and fluorescently-tagged probe *N*-dodecanoyl-L-homoserine lactone-3-hydrazone-fluorescein, C_37_H_40_N_4_O_8_S, (3O-C_12_-HSL-FITC). As additional controls for the bioassay, bacteria reporter in LB medium (not shown) or in LB medium containing diluents was used (*E.coli* reporter alone). Luminescence was measured after 4-h growth. Displayed are the mean ± standard errors of at least six independent experiments performed on separate days. Significant differences (*) in mean for luminescence compared with values for luminescence of *E.coli* reporter alone as a control as calculated by Student's *t* test. (B) Effect of synthetic AHL molecules on ZO-3 junction protein distribution in human epithelial Caco-2 cells. Caco-2 cell monolayers were treated with 1 µM 3O-C_12_-HSL, 3O-C_12_-HSL-3H-biotin, 3O-C_12_-HSL-biotin, 3O-C_12_-HSL-FITC (green) or diluents as a control, for 5 h. Cells were fixed and stained with antibodies against ZO-3 and Alexa Fluor 594 secondary antibodies (red) and analyzed by confocal laser scanning microscopy. The images are from one representative of at least three independent experiments. Image size is 67.6×67.6 µm and pixel size is 0.13 µm.
(TIF)

Figure S2 **Effect of 3O-C_12_-HSL on migration of epithelial Caco-2 cells.** Caco-2 cells were cultured to form monolayers on tissue culture-, rat tail collagen-, or human fibronectin-coated 96-well plates with a cylinder-like plug in each well. The circular, 2 mm diameter wound was created by removing the plug. Cells were incubated with 12 and 200 µM 3O-C_12_-HSL. Control cells were either untreated (data not shown) or treated with 0.018% DMSO as a diluent control. For each well, one image was taken at 0, 24, 48 and 72 h. The migration rate was calculated by measuring the diameter of the wounds (three measurements per image for each well and each time point) using Image J software. Shown is the mean ± standard errors of at least three independent experiments in eight identical wells performed on separate days from different cell passages. Significant differences (* - *P*≤0.05; ** - *P*≤0.01) in mean for migration rate compared with values for cells in the control group as calculated by Student's *t* test.
(TIF)

Figure S3 **Proliferation of epithelial Caco-2 cells treated with 3O-C_12_-HSL.** Cell monolayers cultured in 96-well plates were treated with 6, 12 and 200 µM 3O-C_12_-HSL for 4.5 or 24 h. Control cells were untreated or treated with 0.018% DMSO. This figure shows the mean ± standard error based on at least six independent experiments in eight identical wells performed on different days.
(TIF)

Figure S4 **SDS-PAGE of 3O-C_12_-HSL-3H-biotin complexes from Caco-2 cells.** Total-cell lysate, cytoplasmic or membrane fraction was incubated with 0.05 mg 3O-C_12_-HSL-3H-biotin, 0.05 mg 3O-C_12_-HSL, and 4 µg biotin or without any additions (as controls). Streptavidin agarose resin-captured complexes were analyzed by SDS-PAGE and subsequently stained with PageBlue protein staining solution. Shown are representative gels from one of three independent experiments performed on separate days from different reactions, fraction isolation and cell passages. Bands C1 and C12 represent proteins IQGAP1 and 2 respectively identified by in-gel digestion and LC-MS/MS analysis as shown in Table 1. Background protein contaminants are shown in Table S1. Peptide identification views from MASCOT MS data analyses of IQGAP1 and 2 are shown in supporting information (Dataset S1 and S2).
(TIF)

Figure S5 **SDS-PAGE of 3O-C_12_-HSL-biotin complexes from Caco-2 cells.** Total-cell lysate, cytoplasmic or membrane fraction was incubated with 0.05 mg 3O-C_12_-HSL-biotin, 0.05 mg 3O-C_12_-HSL, and 4 µg biotin or without any additions (as controls). Streptavidin agarose resin-captured complexes were analyzed by SDS-PAGE and subsequently stained with PageBlue protein staining solution. Displayed are representative gels from one of three independent experiments performed on separate days from different reactions, fraction isolation and cell passages. Indicated bands represent proteins identified by in-gel digestion and LC-MS/MS analysis as shown in Table S2.
(TIF)

Protocol S1 (DOCX)

Table S1 **Proteins identified in 3O-C_12_-HSL-3H-biotin affinity complexes from Caco-2 cells using in-gel digestion and LC-MS/MS analysis.**
(DOCX)

Table S2 **Background proteins from 3O-C_12_-HSL-biotin affinity procedure in Caco-2 cells.**
(DOCX)
