# Supplementary material for: The Pseudomonas aeruginosa N-Acylhomoserine Lactone Quorum Sensing Molecules Target IQGAP1 and Modulate Epithelial Cell Migration
Source: PLoS Pathog. 2012 Oct 11;8(10):e1002953. doi: 10.1371/journal.ppat.1002953 (PMC3469656; doi:10.1371/journal.ppat.1002953)
Supplement: Dataset S1 — Peptide identification views from MASCOT MS data analyses of IQGAP1 peptides sequenced by collision-induced dissociation of their ions. The spectra and corresponding lists of fragment ions identified in the MASCOT search are shown. (DOCX) [file ppat.1002953.s001.docx]

MS/MS Fragmentation of **NLGSIAK**
Found in **IQGA1_HUMAN**, Ras GTPase-activating-like protein IQGAP1 OS=Homo sapiens GN=IQGAP1 PE=1 SV=1

Match to Query 513: 701.512724 from(702.520000,1+) intensity(67634.0000) index(261)


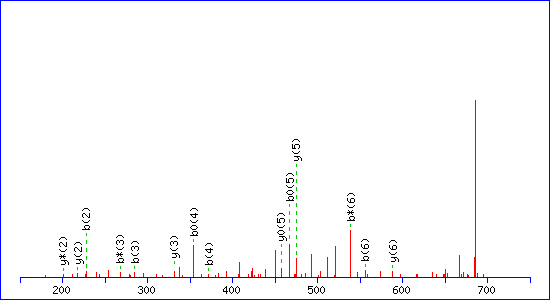


**Monoisotopic mass of neutral peptide Mr(calc):** 701.4072

**Matches :** 14/30 fragment ions using 34 most intense peaks

| **#** | **b** | **b*** | **b^0^** | **Seq.** | **y** | **y*** | **y^0^** | **#** |
| --- | --- | --- | --- | --- | --- | --- | --- | --- |
| **1** | 115.0502 | 98.0237 |  | **N** |  |  |  | **7** |
| **2** | ***228.1343*** | 211.1077 |  | **L** | ***588.3715*** | 571.3450 | 570.3610 | **6** |
| **3** | ***285.1557*** | 268.1292 |  | **G** | ***475.2875*** | 458.2609 | 457.2769 | **5** |
| **4** | ***372.1878*** | 355.1612 | 354.1772 | **S** | 418.2660 | 401.2395 | 400.2554 | **4** |
| **5** | 485.2718 | 468.2453 | 467.2613 | **I** | ***331.2340*** | 314.2074 |  | **3** |
| **6** | ***556.3089*** | 539.2824 | 538.2984 | **A** | ***218.1499*** | 201.1234 |  | **2** |
| **7** |  |  |  | **K** | 147.1128 | 130.0863 |  | **1** |

MS/MS Fragmentation of **LIVDVIR**
Found in **IQGA1_HUMAN**, Ras GTPase-activating-like protein IQGAP1 OS=Homo sapiens GN=IQGAP1 PE=1 SV=1

Match to Query 585: 826.665448 from(414.340000,2+) intensity(173580.0000) index(352)


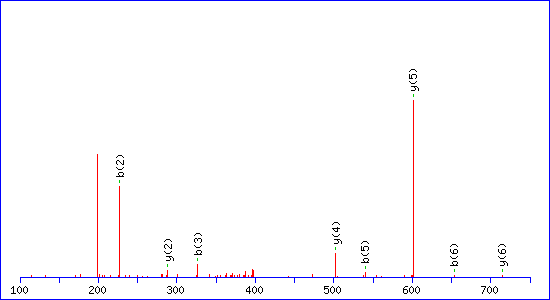


**Monoisotopic mass of neutral peptide Mr(calc):** 826.5276

**Matches :** 8/48 fragment ions using 12 most intense peaks

| **#** | **b** | **b^++^** | **b^0^** | **b^0++^** | **Seq.** | **y** | **y^++^** | **y*** | **y*^++^** | **y^0^** | **y^0++^** | **#** |
| --- | --- | --- | --- | --- | --- | --- | --- | --- | --- | --- | --- | --- |
| **1** | 114.0913 | 57.5493 |  |  | **L** |  |  |  |  |  |  | **7** |
| **2** | ***227.1754*** | 114.0913 |  |  | **I** | ***714.4509*** | 357.7291 | 697.4243 | 349.2158 | 696.4403 | 348.7238 | **6** |
| **3** | ***326.2438*** | 163.6255 |  |  | **V** | ***601.3668*** | 301.1870 | 584.3402 | 292.6738 | 583.3562 | 292.1817 | **5** |
| **4** | 441.2708 | 221.1390 | 423.2602 | 212.1337 | **D** | ***502.2984*** | 251.6528 | 485.2718 | 243.1396 | 484.2878 | 242.6475 | **4** |
| **5** | ***540.3392*** | 270.6732 | 522.3286 | 261.6679 | **V** | 387.2714 | 194.1394 | 370.2449 | 185.6261 |  |  | **3** |
| **6** | ***653.4232*** | 327.2153 | 635.4127 | 318.2100 | **I** | ***288.2030*** | 144.6051 | 271.1765 | 136.0919 |  |  | **2** |
| **7** |  |  |  |  | **R** | 175.1190 | 88.0631 | 158.0924 | 79.5498 |  |  | **1** |

MS/MS Fragmentation of **MVVSFNR**
Found in **IQGA1_HUMAN**, Ras GTPase-activating-like protein IQGAP1 OS=Homo sapiens GN=IQGAP1 PE=1 SV=1

Match to Query 596: 867.565448 from(434.790000,2+) intensity(198486.0000) index(287)


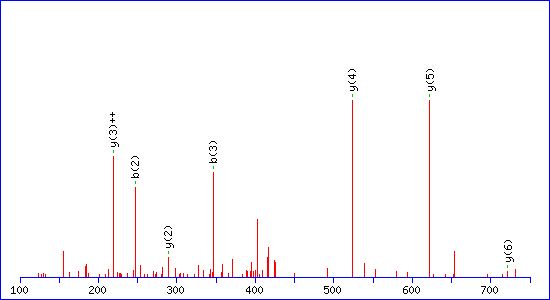


**Monoisotopic mass of neutral peptide Mr(calc):** 867.4273

**Variable modifications:**

**M1 :** Oxidation (M), with neutral losses 0.0000(shown in table), 63.9983

**Matches :** 7/70 fragment ions using 13 most intense peaks

| **#** | **b** | **b^++^** | **b*** | **b*^++^** | **b^0^** | **b^0++^** | **Seq.** | **y** | **y^++^** | **y*** | **y*^++^** | **y^0^** | **y^0++^** | **#** |
| --- | --- | --- | --- | --- | --- | --- | --- | --- | --- | --- | --- | --- | --- | --- |
| **1** | 148.0427 | 74.5250 |  |  |  |  | **M** |  |  |  |  |  |  | **7** |
| **2** | **247.1111** | 124.0592 |  |  |  |  | **V** | ***721.3991*** | 361.2032 | 704.3726 | 352.6899 | 703.3886 | 352.1979 | **6** |
| **3** | **346.1795** | 173.5934 |  |  |  |  | **V** | ***622.3307*** | 311.6690 | 605.3042 | 303.1557 | 604.3202 | 302.6637 | **5** |
| **4** | 433.2115 | 217.1094 |  |  | 415.2010 | 208.1041 | **S** | ***523.2623*** | 262.1348 | 506.2358 | 253.6215 | 505.2518 | 253.1295 | **4** |
| **5** | 580.2799 | 290.6436 |  |  | 562.2694 | 281.6383 | **F** | 436.2303 | 218.6188 | 419.2037 | 210.1055 |  |  | **3** |
| **6** | 694.3229 | 347.6651 | 677.2963 | 339.1518 | 676.3123 | 338.6598 | **N** | ***289.1619*** | 145.0846 | 272.1353 | 136.5713 |  |  | **2** |
| **7** |  |  |  |  |  |  | **R** | 175.1190 | 88.0631 | 158.0924 | 79.5498 |  |  | **1** |

MS/MS Fragmentation of **LGNFFSPK**
Found in **IQGA1_HUMAN**, Ras GTPase-activating-like protein IQGAP1 OS=Homo sapiens GN=IQGAP1 PE=1 SV=1

Match to Query 612: 908.745448 from(455.380000,2+) intensity(403448.0000) index(349)


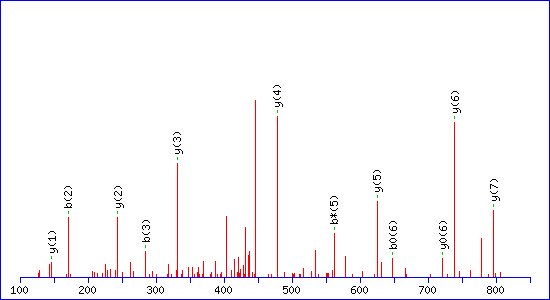


**Monoisotopic mass of neutral peptide Mr(calc):** 908.4756

**Matches :** 12/66 fragment ions using 14 most intense peaks

| **#** | **b** | **b^++^** | **b*** | **b*^++^** | **b^0^** | **b^0++^** | **Seq.** | **y** | **y^++^** | **y*** | **y*^++^** | **y^0^** | **y^0++^** | **#** |
| --- | --- | --- | --- | --- | --- | --- | --- | --- | --- | --- | --- | --- | --- | --- |
| **1** | 114.0913 | 57.5493 |  |  |  |  | **L** |  |  |  |  |  |  | **8** |
| **2** | **171.1128** | 86.0600 |  |  |  |  | **G** | ***796.3988*** | 398.7030 | 779.3723 | 390.1898 | 778.3882 | 389.6978 | **7** |
| **3** | **285.1557** | 143.0815 | 268.1292 | 134.5682 |  |  | **N** | ***739.3774*** | 370.1923 | 722.3508 | 361.6790 | 721.3668 | 361.1870 | **6** |
| **4** | 432.2241 | 216.6157 | 415.1976 | 208.1024 |  |  | **F** | ***625.3344*** | 313.1709 | 608.3079 | 304.6576 | 607.3239 | 304.1656 | **5** |
| **5** | 579.2926 | 290.1499 | 562.2660 | 281.6366 |  |  | **F** | ***478.2660*** | 239.6366 | 461.2395 | 231.1234 | 460.2554 | 230.6314 | **4** |
| **6** | 666.3246 | 333.6659 | 649.2980 | 325.1527 | 648.3140 | 324.6606 | **S** | ***331.1976*** | 166.1024 | 314.1710 | 157.5892 | 313.1870 | 157.0972 | **3** |
| **7** | 763.3774 | 382.1923 | 746.3508 | 373.6790 | 745.3668 | 373.1870 | **P** | ***244.1656*** | 122.5864 | 227.1390 | 114.0731 |  |  | **2** |
| **8** |  |  |  |  |  |  | **K** | ***147.1128*** | 74.0600 | 130.0863 | 65.5468 |  |  | **1** |

MS/MS Fragmentation of **TALQEEIK**
Found in **IQGA1_HUMAN**, Ras GTPase-activating-like protein IQGAP1 OS=Homo sapiens GN=IQGAP1 PE=1 SV=1

Match to Query 623: 930.645448 from(466.330000,2+) intensity(170587.0000) index(285)


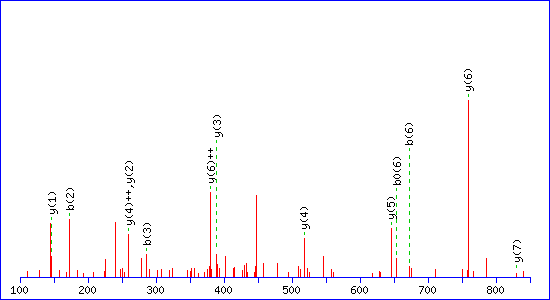


**Monoisotopic mass of neutral peptide Mr(calc):** 930.5022

**Matches :** 13/74 fragment ions using 23 most intense peaks

| **#** | **b** | **b^++^** | **b*** | **b*^++^** | **b^0^** | **b^0++^** | **Seq.** | **y** | **y^++^** | **y*** | **y*^++^** | **y^0^** | **y^0++^** | **#** |
| --- | --- | --- | --- | --- | --- | --- | --- | --- | --- | --- | --- | --- | --- | --- |
| **1** | 102.0550 | 51.5311 |  |  | 84.0444 | 42.5258 | **T** |  |  |  |  |  |  | **8** |
| **2** | **173.0921** | 87.0497 |  |  | 155.0815 | 78.0444 | **A** | ***830.4618*** | 415.7345 | 813.4353 | 407.2213 | 812.4512 | 406.7293 | **7** |
| **3** | **286.1761** | 143.5917 |  |  | 268.1656 | 134.5864 | **L** | ***759.4247*** | 380.2160 | 742.3981 | 371.7027 | 741.4141 | 371.2107 | **6** |
| **4** | 414.2347 | 207.6210 | 397.2082 | 199.1077 | 396.2241 | 198.6157 | **Q** | ***646.3406*** | 323.6740 | 629.3141 | 315.1607 | 628.3301 | 314.6687 | **5** |
| **5** | 543.2773 | 272.1423 | 526.2508 | 263.6290 | 525.2667 | 263.1370 | **E** | ***518.2821*** | 259.6447 | 501.2555 | 251.1314 | 500.2715 | 250.6394 | **4** |
| **6** | **672.3199** | 336.6636 | 655.2933 | 328.1503 | 654.3093 | 327.6583 | **E** | ***389.2395*** | 195.1234 | 372.2129 | 186.6101 | 371.2289 | 186.1181 | **3** |
| **7** | 785.4040 | 393.2056 | 768.3774 | 384.6923 | 767.3934 | 384.2003 | **I** | ***260.1969*** | 130.6021 | 243.1703 | 122.0888 |  |  | **2** |
| **8** |  |  |  |  |  |  | **K** | ***147.1128*** | 74.0600 | 130.0863 | 65.5468 |  |  | **1** |

MS/MS Fragmentation of **TILLNTKR**
Found in **IQGA1_HUMAN**, Ras GTPase-activating-like protein IQGAP1 OS=Homo sapiens GN=IQGAP1 PE=1 SV=1

Match to Query 635: 957.785448 from(479.900000,2+) intensity(154880.0000) index(270)


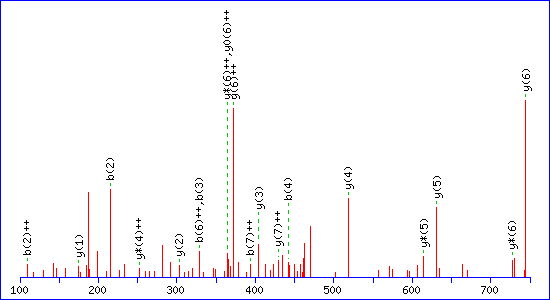


**Monoisotopic mass of neutral peptide Mr(calc):** 957.5971

**Matches :** 19/72 fragment ions using 44 most intense peaks

| **#** | **b** | **b^++^** | **b*** | **b*^++^** | **b^0^** | **b^0++^** | **Seq.** | **y** | **y^++^** | **y*** | **y*^++^** | **y^0^** | **y^0++^** | **#** |
| --- | --- | --- | --- | --- | --- | --- | --- | --- | --- | --- | --- | --- | --- | --- |
| **1** | 102.0550 | 51.5311 |  |  | 84.0444 | 42.5258 | **T** |  |  |  |  |  |  | **8** |
| **2** | **215.1390** | **108.0731** |  |  | 197.1285 | 99.0679 | **I** | 857.5567 | 429.2820 | 840.5302 | 420.7687 | 839.5461 | 420.2767 | **7** |
| **3** | **328.2231** | 164.6152 |  |  | 310.2125 | 155.6099 | **L** | ***744.4726*** | 372.7400 | 727.4461 | 364.2267 | 726.4621 | 363.7347 | **6** |
| **4** | **441.3071** | 221.1572 |  |  | 423.2966 | 212.1519 | **L** | ***631.3886*** | 316.1979 | 614.3620 | 307.6847 | 613.3780 | 307.1926 | **5** |
| **5** | 555.3501 | 278.1787 | 538.3235 | 269.6654 | 537.3395 | 269.1734 | **N** | ***518.3045*** | 259.6559 | 501.2780 | 251.1426 | 500.2940 | 250.6506 | **4** |
| **6** | 656.3978 | **328.7025** | 639.3712 | 320.1892 | 638.3872 | 319.6972 | **T** | ***404.2616*** | 202.6344 | 387.2350 | 194.1212 | 386.2510 | 193.6292 | **3** |
| **7** | 784.4927 | **392.7500** | 767.4662 | 384.2367 | 766.4822 | 383.7447 | **K** | ***303.2139*** | 152.1106 | 286.1874 | 143.5973 |  |  | **2** |
| **8** |  |  |  |  |  |  | **R** | ***175.1190*** | 88.0631 | 158.0924 | 79.5498 |  |  | **1** |

MS/MS Fragmentation of **LTELGTVDPK**
Found in **IQGA1_HUMAN**, Ras GTPase-activating-like protein IQGAP1 OS=Homo sapiens GN=IQGAP1 PE=1 SV=1

Match to Query 677: 1071.805448 from(536.910000,2+) intensity(242942.0000) index(299)


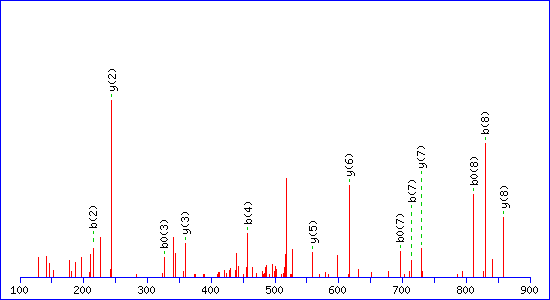


**Monoisotopic mass of neutral peptide Mr(calc):** 1071.5812

**Matches :** 13/84 fragment ions using 16 most intense peaks

| **#** | **b** | **b^++^** | **b^0^** | **b^0++^** | **Seq.** | **y** | **y^++^** | **y*** | **y*^++^** | **y^0^** | **y^0++^** | **#** |
| --- | --- | --- | --- | --- | --- | --- | --- | --- | --- | --- | --- | --- |
| **1** | 114.0913 | 57.5493 |  |  | **L** |  |  |  |  |  |  | **10** |
| **2** | **215.1390** | 108.0731 | 197.1285 | 99.0679 | **T** | 959.5044 | 480.2558 | 942.4779 | 471.7426 | 941.4938 | 471.2506 | **9** |
| **3** | 344.1816 | 172.5944 | 326.1710 | 163.5892 | **E** | ***858.4567*** | 429.7320 | 841.4302 | 421.2187 | 840.4462 | 420.7267 | **8** |
| **4** | **457.2657** | 229.1365 | 439.2551 | 220.1312 | **L** | ***729.4141*** | 365.2107 | 712.3876 | 356.6974 | 711.4036 | 356.2054 | **7** |
| **5** | 514.2871 | 257.6472 | 496.2766 | 248.6419 | **G** | ***616.3301*** | 308.6687 | 599.3035 | 300.1554 | 598.3195 | 299.6634 | **6** |
| **6** | 615.3348 | 308.1710 | 597.3243 | 299.1658 | **T** | ***559.3086*** | 280.1579 | 542.2821 | 271.6447 | 541.2980 | 271.1527 | **5** |
| **7** | **714.4032** | 357.7053 | 696.3927 | 348.7000 | **V** | 458.2609 | 229.6341 | 441.2344 | 221.1208 | 440.2504 | 220.6288 | **4** |
| **8** | **829.4302** | 415.2187 | 811.4196 | 406.2134 | **D** | ***359.1925*** | 180.0999 | 342.1660 | 171.5866 | 341.1819 | 171.0946 | **3** |
| **9** | 926.4829 | 463.7451 | 908.4724 | 454.7398 | **P** | ***244.1656*** | 122.5864 | 227.1390 | 114.0731 |  |  | **2** |
| **10** |  |  |  |  | **K** | 147.1128 | 74.0600 | 130.0863 | 65.5468 |  |  | **1** |

MS/MS Fragmentation of **LIFQMPQNK**
Found in **IQGA1_HUMAN**, Ras GTPase-activating-like protein IQGAP1 OS=Homo sapiens GN=IQGAP1 PE=1 SV=1

Match to Query 706: 1133.785448 from(567.900000,2+) intensity(365004.0000) index(313)


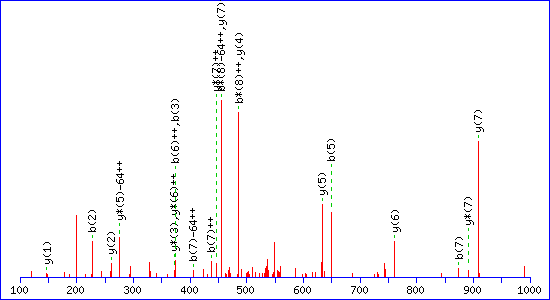


**Monoisotopic mass of neutral peptide Mr(calc):** 1133.5903

**Variable modifications:**

**M5 :** Oxidation (M), with neutral losses 0.0000(shown in table), 63.9983

**Matches :** 21/90 fragment ions using 33 most intense peaks

| **#** | **b** | **b^++^** | **b*** | **b*^++^** | **Seq.** | **y** | **y^++^** | **y*** | **y*^++^** | **#** |
| --- | --- | --- | --- | --- | --- | --- | --- | --- | --- | --- |
| **1** | 114.0913 | 57.5493 |  |  | **L** |  |  |  |  | **9** |
| **2** | **227.1754** | 114.0913 |  |  | **I** | 1021.5135 | 511.2604 | 1004.4870 | 502.7471 | **8** |
| **3** | **374.2438** | 187.6255 |  |  | **F** | ***908.4295*** | 454.7184 | 891.4029 | 446.2051 | **7** |
| **4** | 502.3024 | 251.6548 | 485.2758 | 243.1416 | **Q** | ***761.3611*** | 381.1842 | 744.3345 | 372.6709 | **6** |
| **5** | **649.3378** | 325.1725 | 632.3112 | 316.6593 | **M** | ***633.3025*** | 317.1549 | 616.2759 | 308.6416 | **5** |
| **6** | 746.3906 | 373.6989 | 729.3640 | 365.1856 | **P** | ***486.2671*** | 243.6372 | 469.2405 | 235.1239 | **4** |
| **7** | **874.4491** | 437.7282 | 857.4226 | 429.2149 | **Q** | 389.2143 | 195.1108 | 372.1878 | 186.5975 | **3** |
| **8** | 988.4921 | 494.7497 | 971.4655 | 486.2364 | **N** | ***261.1557*** | 131.0815 | 244.1292 | 122.5682 | **2** |
| **9** |  |  |  |  | **K** | ***147.1128*** | 74.0600 | 130.0863 | 65.5468 | **1** |

MS/MS Fragmentation of **YGIQMPAFSK**
Found in **IQGA1_HUMAN**, Ras GTPase-activating-like protein IQGAP1 OS=Homo sapiens GN=IQGAP1 PE=1 SV=1

Match to Query 712: 1156.685448 from(579.350000,2+) intensity(362726.0000) index(316)


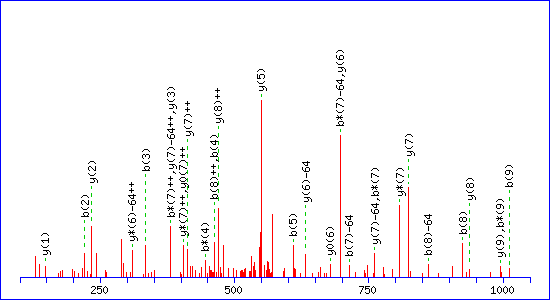


**Monoisotopic mass of neutral peptide Mr(calc):** 1156.5587

**Variable modifications:**

**M5 :** Oxidation (M), with neutral losses 0.0000(shown in table), 63.9983

**Matches :** 32/130 fragment ions using 36 most intense peaks

| **#** | **b** | **b^++^** | **b*** | **b*^++^** | **b^0^** | **b^0++^** | **Seq.** | **y** | **y^++^** | **y*** | **y*^++^** | **y^0^** | **y^0++^** | **#** |
| --- | --- | --- | --- | --- | --- | --- | --- | --- | --- | --- | --- | --- | --- | --- |
| **1** | 164.0706 | 82.5389 |  |  |  |  | **Y** |  |  |  |  |  |  | **10** |
| **2** | **221.0921** | 111.0497 |  |  |  |  | **G** | ***994.5026*** | 497.7550 | 977.4761 | 489.2417 | 976.4921 | 488.7497 | **9** |
| **3** | **334.1761** | 167.5917 |  |  |  |  | **I** | ***937.4812*** | 469.2442 | 920.4546 | 460.7309 | 919.4706 | 460.2389 | **8** |
| **4** | **462.2347** | 231.6210 | 445.2082 | 223.1077 |  |  | **Q** | ***824.3971*** | 412.7022 | 807.3706 | 404.1889 | 806.3865 | 403.6969 | **7** |
| **5** | **609.2701** | 305.1387 | 592.2436 | 296.6254 |  |  | **M** | ***696.3385*** | 348.6729 | 679.3120 | 340.1596 | 678.3280 | 339.6676 | **6** |
| **6** | 706.3229 | 353.6651 | 689.2963 | 345.1518 |  |  | **P** | ***549.3031*** | 275.1552 | 532.2766 | 266.6419 | 531.2926 | 266.1499 | **5** |
| **7** | 777.3600 | 389.1836 | 760.3334 | 380.6704 |  |  | **A** | 452.2504 | 226.6288 | 435.2238 | 218.1155 | 434.2398 | 217.6235 | **4** |
| **8** | **924.4284** | 462.7178 | 907.4019 | 454.2046 |  |  | **F** | ***381.2132*** | 191.1103 | 364.1867 | 182.5970 | 363.2027 | 182.1050 | **3** |
| **9** | **1011.4604** | 506.2339 | 994.4339 | 497.7206 | 993.4499 | 497.2286 | **S** | ***234.1448*** | 117.5761 | 217.1183 | 109.0628 | 216.1343 | 108.5708 | **2** |
| **10** |  |  |  |  |  |  | **K** | ***147.1128*** | 74.0600 | 130.0863 | 65.5468 |  |  | **1** |

MS/MS Fragmentation of **LQQTYAALNSK**
Found in **IQGA1_HUMAN**, Ras GTPase-activating-like protein IQGAP1 OS=Homo sapiens GN=IQGAP1 PE=1 SV=1

Match to Query 727: 1235.825448 from(618.920000,2+) intensity(272920.0000) index(286)


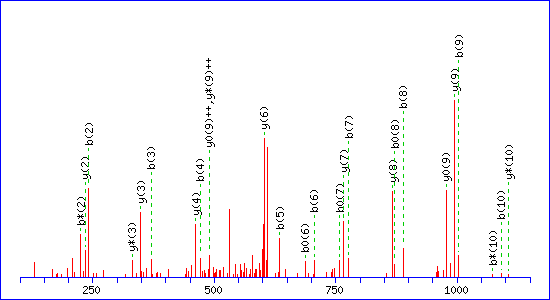


**Monoisotopic mass of neutral peptide Mr(calc):** 1235.6510

**Matches :** 26/110 fragment ions using 30 most intense peaks

| **#** | **b** | **b^++^** | **b*** | **b*^++^** | **b^0^** | **b^0++^** | **Seq.** | **y** | **y^++^** | **y*** | **y*^++^** | **y^0^** | **y^0++^** | **#** |
| --- | --- | --- | --- | --- | --- | --- | --- | --- | --- | --- | --- | --- | --- | --- |
| **1** | 114.0913 | 57.5493 |  |  |  |  | **L** |  |  |  |  |  |  | **11** |
| **2** | ***242.1499*** | 121.5786 | 225.1234 | 113.0653 |  |  | **Q** | 1123.5742 | 562.2907 | 1106.5477 | 553.7775 | 1105.5637 | 553.2855 | **10** |
| **3** | ***370.2085*** | 185.6079 | 353.1819 | 177.0946 |  |  | **Q** | **995.5156** | 498.2615 | 978.4891 | 489.7482 | 977.5051 | 489.2562 | **9** |
| **4** | ***471.2562*** | 236.1317 | 454.2296 | 227.6185 | 453.2456 | 227.1264 | **T** | **867.4571** | 434.2322 | 850.4305 | 425.7189 | 849.4465 | 425.2269 | **8** |
| **5** | ***634.3195*** | 317.6634 | 617.2930 | 309.1501 | 616.3089 | 308.6581 | **Y** | **766.4094** | 383.7083 | 749.3828 | 375.1951 | 748.3988 | 374.7030 | **7** |
| **6** | ***705.3566*** | 353.1819 | 688.3301 | 344.6687 | 687.3461 | 344.1767 | **A** | **603.3461** | 302.1767 | 586.3195 | 293.6634 | 585.3355 | 293.1714 | **6** |
| **7** | ***776.3937*** | 388.7005 | 759.3672 | 380.1872 | 758.3832 | 379.6952 | **A** | 532.3089 | 266.6581 | 515.2824 | 258.1448 | 514.2984 | 257.6528 | **5** |
| **8** | ***889.4778*** | 445.2425 | 872.4512 | 436.7293 | 871.4672 | 436.2373 | **L** | **461.2718** | 231.1395 | 444.2453 | 222.6263 | 443.2613 | 222.1343 | **4** |
| **9** | ***1003.5207*** | 502.2640 | 986.4942 | 493.7507 | 985.5102 | 493.2587 | **N** | **348.1878** | 174.5975 | 331.1612 | 166.0842 | 330.1772 | 165.5922 | **3** |
| **10** | ***1090.5528*** | 545.7800 | 1073.5262 | 537.2667 | 1072.5422 | 536.7747 | **S** | **234.1448** | 117.5761 | 217.1183 | 109.0628 | 216.1343 | 108.5708 | **2** |
| **11** |  |  |  |  |  |  | **K** | 147.1128 | 74.0600 | 130.0863 | 65.5468 |  |  | **1** |

MS/MS Fragmentation of **LAAVALINAAIQK**
Found in **IQGA1_HUMAN**, Ras GTPase-activating-like protein IQGAP1 OS=Homo sapiens GN=IQGAP1 PE=1 SV=1

Match to Query 738: 1294.985448 from(648.500000,2+) intensity(399955.0000) index(402)


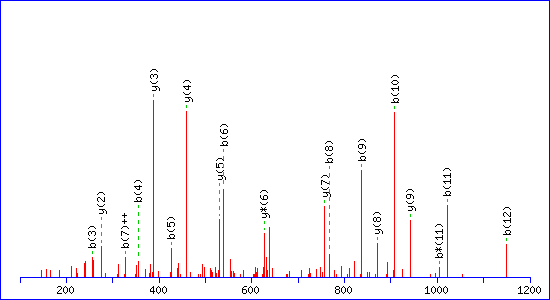


**Monoisotopic mass of neutral peptide Mr(calc):** 1294.7972

**Matches :** 19/82 fragment ions using 29 most intense peaks

| **#** | **b** | **b^++^** | **b*** | **b*^++^** | **Seq.** | **y** | **y^++^** | **y*** | **y*^++^** | **#** |
| --- | --- | --- | --- | --- | --- | --- | --- | --- | --- | --- |
| **1** | 114.0913 | 57.5493 |  |  | **L** |  |  |  |  | **13** |
| **2** | 185.1285 | 93.0679 |  |  | **A** | 1182.7205 | 591.8639 | 1165.6939 | 583.3506 | **12** |
| **3** | ***256.1656*** | 128.5864 |  |  | **A** | 1111.6834 | 556.3453 | 1094.6568 | 547.8320 | **11** |
| **4** | ***355.2340*** | 178.1206 |  |  | **V** | 1040.6463 | 520.8268 | 1023.6197 | 512.3135 | **10** |
| **5** | ***426.2711*** | 213.6392 |  |  | **A** | **941.5778** | 471.2926 | 924.5513 | 462.7793 | **9** |
| **6** | ***539.3552*** | 270.1812 |  |  | **L** | **870.5407** | 435.7740 | 853.5142 | 427.2607 | **8** |
| **7** | 652.4392 | 326.7233 |  |  | **I** | **757.4567** | 379.2320 | 740.4301 | 370.7187 | **7** |
| **8** | ***766.4822*** | 383.7447 | 749.4556 | 375.2314 | **N** | 644.3726 | 322.6899 | 627.3461 | 314.1767 | **6** |
| **9** | ***837.5193*** | 419.2633 | 820.4927 | 410.7500 | **A** | **530.3297** | 265.6685 | 513.3031 | 257.1552 | **5** |
| **10** | ***908.5564*** | 454.7818 | 891.5298 | 446.2686 | **A** | **459.2926** | 230.1499 | 442.2660 | 221.6366 | **4** |
| **11** | ***1021.6404*** | 511.3239 | 1004.6139 | 502.8106 | **I** | **388.2554** | 194.6314 | 371.2289 | 186.1181 | **3** |
| **12** | ***1149.6990*** | 575.3531 | 1132.6725 | 566.8399 | **Q** | **275.1714** | 138.0893 | 258.1448 | 129.5761 | **2** |
| **13** |  |  |  |  | **K** | 147.1128 | 74.0600 | 130.0863 | 65.5468 | **1** |

MS/MS Fragmentation of **ALESGDVNTVWK**
Found in **IQGA1_HUMAN**, Ras GTPase-activating-like protein IQGAP1 OS=Homo sapiens GN=IQGAP1 PE=1 SV=1

Match to Query 740: 1317.945448 from(659.980000,2+) intensity(266859.0000) index(335)


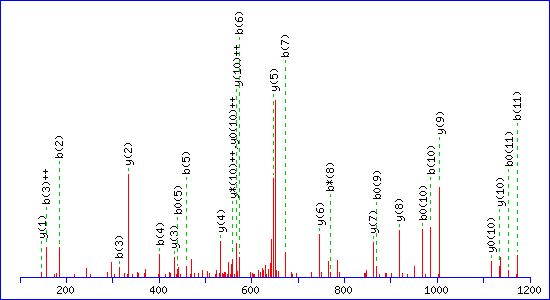


**Monoisotopic mass of neutral peptide Mr(calc):** 1317.6565

**Matches :** 28/108 fragment ions using 42 most intense peaks

| **#** | **b** | **b^++^** | **b*** | **b*^++^** | **b^0^** | **b^0++^** | **Seq.** | **y** | **y^++^** | **y*** | **y*^++^** | **y^0^** | **y^0++^** | **#** |
| --- | --- | --- | --- | --- | --- | --- | --- | --- | --- | --- | --- | --- | --- | --- |
| **1** | 72.0444 | 36.5258 |  |  |  |  | **A** |  |  |  |  |  |  | **12** |
| **2** | **185.1285** | 93.0679 |  |  |  |  | **L** | 1247.6266 | 624.3170 | 1230.6001 | 615.8037 | 1229.6161 | 615.3117 | **11** |
| **3** | **314.1710** | 157.5892 |  |  | 296.1605 | 148.5839 | **E** | ***1134.5426*** | 567.7749 | 1117.5160 | 559.2617 | 1116.5320 | 558.7696 | **10** |
| **4** | **401.2031** | 201.1052 |  |  | 383.1925 | 192.0999 | **S** | ***1005.5000*** | 503.2536 | 988.4734 | 494.7404 | 987.4894 | 494.2483 | **9** |
| **5** | **458.2245** | 229.6159 |  |  | 440.2140 | 220.6106 | **G** | ***918.4680*** | 459.7376 | 901.4414 | 451.2243 | 900.4574 | 450.7323 | **8** |
| **6** | **573.2515** | 287.1294 |  |  | 555.2409 | 278.1241 | **D** | ***861.4465*** | 431.2269 | 844.4199 | 422.7136 | 843.4359 | 422.2216 | **7** |
| **7** | **672.3199** | 336.6636 |  |  | 654.3093 | 327.6583 | **V** | ***746.4196*** | 373.7134 | 729.3930 | 365.2001 | 728.4090 | 364.7081 | **6** |
| **8** | 786.3628 | 393.6850 | 769.3363 | 385.1718 | 768.3523 | 384.6798 | **N** | ***647.3511*** | 324.1792 | 630.3246 | 315.6659 | 629.3406 | 315.1739 | **5** |
| **9** | 887.4105 | 444.2089 | 870.3840 | 435.6956 | 869.3999 | 435.2036 | **T** | ***533.3082*** | 267.1577 | 516.2817 | 258.6445 | 515.2976 | 258.1525 | **4** |
| **10** | **986.4789** | 493.7431 | 969.4524 | 485.2298 | 968.4684 | 484.7378 | **V** | ***432.2605*** | 216.6339 | 415.2340 | 208.1206 |  |  | **3** |
| **11** | **1172.5582** | 586.7828 | 1155.5317 | 578.2695 | 1154.5477 | 577.7775 | **W** | ***333.1921*** | 167.0997 | 316.1656 | 158.5864 |  |  | **2** |
| **12** |  |  |  |  |  |  | **K** | ***147.1128*** | 74.0600 | 130.0863 | 65.5468 |  |  | **1** |

MS/MS Fragmentation of **SVKEDSNLTLQEK**
Found in **IQGA1_HUMAN**, Ras GTPase-activating-like protein IQGAP1 OS=Homo sapiens GN=IQGAP1 PE=1 SV=1

Match to Query 762: 1490.018172 from(497.680000,3+) intensity(69343.0000) index(268)


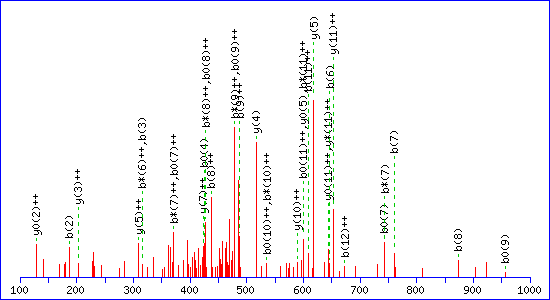


**Monoisotopic mass of neutral peptide Mr(calc):** 1489.7624

**Matches :** 35/138 fragment ions using 46 most intense peaks

| **#** | **b** | **b^++^** | **b*** | **b*^++^** | **b^0^** | **b^0++^** | **Seq.** | **y** | **y^++^** | **y*** | **y*^++^** | **y^0^** | **y^0++^** | **#** |
| --- | --- | --- | --- | --- | --- | --- | --- | --- | --- | --- | --- | --- | --- | --- |
| **1** | 88.0393 | 44.5233 |  |  | 70.0287 | 35.5180 | **S** |  |  |  |  |  |  | **13** |
| **2** | ***187.1077*** | 94.0575 |  |  | 169.0972 | 85.0522 | **V** | 1403.7377 | 702.3725 | 1386.7111 | 693.8592 | 1385.7271 | 693.3672 | **12** |
| **3** | ***315.2027*** | 158.1050 | 298.1761 | 149.5917 | 297.1921 | 149.0997 | **K** | 1304.6692 | 652.8383 | 1287.6427 | 644.3250 | 1286.6587 | 643.8330 | **11** |
| **4** | 444.2453 | 222.6263 | 427.2187 | 214.1130 | 426.2347 | 213.6210 | **E** | 1176.5743 | 588.7908 | 1159.5477 | 580.2775 | 1158.5637 | 579.7855 | **10** |
| **5** | 559.2722 | 280.1397 | 542.2457 | 271.6265 | 541.2617 | 271.1345 | **D** | 1047.5317 | 524.2695 | 1030.5051 | 515.7562 | 1029.5211 | 515.2642 | **9** |
| **6** | ***646.3042*** | 323.6558 | 629.2777 | 315.1425 | 628.2937 | 314.6505 | **S** | 932.5047 | 466.7560 | 915.4782 | 458.2427 | 914.4942 | 457.7507 | **8** |
| **7** | ***760.3472*** | 380.6772 | 743.3206 | 372.1639 | 742.3366 | 371.6719 | **N** | 845.4727 | 423.2400 | 828.4462 | 414.7267 | 827.4621 | 414.2347 | **7** |
| **8** | ***873.4312*** | ***437.2193*** | 856.4047 | 428.7060 | 855.4207 | 428.2140 | **L** | 731.4298 | 366.2185 | 714.4032 | 357.7053 | 713.4192 | 357.2132 | **6** |
| **9** | 974.4789 | ***487.7431*** | 957.4524 | 479.2298 | 956.4683 | 478.7378 | **T** | 618.3457 | 309.6765 | 601.3192 | 301.1632 | 600.3352 | 300.6712 | **5** |
| **10** | 1087.5630 | 544.2851 | 1070.5364 | 535.7719 | 1069.5524 | 535.2798 | **L** | 517.2980 | 259.1527 | 500.2715 | 250.6394 | 499.2875 | 250.1474 | **4** |
| **11** | 1215.6216 | ***608.3144*** | 1198.5950 | 599.8011 | 1197.6110 | 599.3091 | **Q** | 404.2140 | 202.6106 | 387.1874 | 194.0974 | 386.2034 | 193.6053 | **3** |
| **12** | 1344.6642 | ***672.8357*** | 1327.6376 | 664.3224 | 1326.6536 | 663.8304 | **E** | 276.1554 | 138.5813 | 259.1288 | 130.0681 | 258.1448 | 129.5761 | **2** |
| **13** |  |  |  |  |  |  | **K** | 147.1128 | 74.0600 | 130.0863 | 65.5468 |  |  | **1** |

MS/MS Fragmentation of **SVKEDSNLTLQEK**
Found in **IQGA1_HUMAN**, Ras GTPase-activating-like protein IQGAP1 OS=Homo sapiens GN=IQGAP1 PE=1 SV=1

Match to Query 763: 1490.025448 from(746.020000,2+) intensity(71283.0000) index(269)


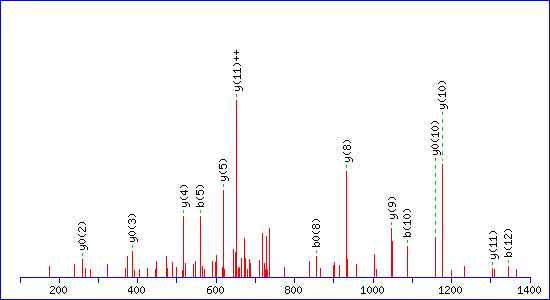


**Monoisotopic mass of neutral peptide Mr(calc):** 1489.7624

**Matches :** 14/138 fragment ions using 24 most intense peaks

| **#** | **b** | **b^++^** | **b*** | **b*^++^** | **b^0^** | **b^0++^** | **Seq.** | **y** | **y^++^** | **y*** | **y*^++^** | **y^0^** | **y^0++^** | **#** |
| --- | --- | --- | --- | --- | --- | --- | --- | --- | --- | --- | --- | --- | --- | --- |
| **1** | 88.0393 | 44.5233 |  |  | 70.0287 | 35.5180 | **S** |  |  |  |  |  |  | **13** |
| **2** | 187.1077 | 94.0575 |  |  | 169.0972 | 85.0522 | **V** | 1403.7377 | 702.3725 | 1386.7111 | 693.8592 | 1385.7271 | 693.3672 | **12** |
| **3** | 315.2027 | 158.1050 | 298.1761 | 149.5917 | 297.1921 | 149.0997 | **K** | ***1304.6692*** | 652.8383 | 1287.6427 | 644.3250 | 1286.6587 | 643.8330 | **11** |
| **4** | 444.2453 | 222.6263 | 427.2187 | 214.1130 | 426.2347 | 213.6210 | **E** | ***1176.5743*** | 588.7908 | 1159.5477 | 580.2775 | 1158.5637 | 579.7855 | **10** |
| **5** | ***559.2722*** | 280.1397 | 542.2457 | 271.6265 | 541.2617 | 271.1345 | **D** | ***1047.5317*** | 524.2695 | 1030.5051 | 515.7562 | 1029.5211 | 515.2642 | **9** |
| **6** | 646.3042 | 323.6558 | 629.2777 | 315.1425 | 628.2937 | 314.6505 | **S** | ***932.5047*** | 466.7560 | 915.4782 | 458.2427 | 914.4942 | 457.7507 | **8** |
| **7** | 760.3472 | 380.6772 | 743.3206 | 372.1639 | 742.3366 | 371.6719 | **N** | 845.4727 | 423.2400 | 828.4462 | 414.7267 | 827.4621 | 414.2347 | **7** |
| **8** | 873.4312 | 437.2193 | 856.4047 | 428.7060 | 855.4207 | 428.2140 | **L** | 731.4298 | 366.2185 | 714.4032 | 357.7053 | 713.4192 | 357.2132 | **6** |
| **9** | 974.4789 | 487.7431 | 957.4524 | 479.2298 | 956.4683 | 478.7378 | **T** | ***618.3457*** | 309.6765 | 601.3192 | 301.1632 | 600.3352 | 300.6712 | **5** |
| **10** | ***1087.5630*** | 544.2851 | 1070.5364 | 535.7719 | 1069.5524 | 535.2798 | **L** | ***517.2980*** | 259.1527 | 500.2715 | 250.6394 | 499.2875 | 250.1474 | **4** |
| **11** | 1215.6216 | 608.3144 | 1198.5950 | 599.8011 | 1197.6110 | 599.3091 | **Q** | 404.2140 | 202.6106 | 387.1874 | 194.0974 | 386.2034 | 193.6053 | **3** |
| **12** | ***1344.6642*** | 672.8357 | 1327.6376 | 664.3224 | 1326.6536 | 663.8304 | **E** | 276.1554 | 138.5813 | 259.1288 | 130.0681 | 258.1448 | 129.5761 | **2** |
| **13** |  |  |  |  |  |  | **K** | 147.1128 | 74.0600 | 130.0863 | 65.5468 |  |  | **1** |

MS/MS Fragmentation of **EQLWLANEGLITR**
Found in **IQGA1_HUMAN**, Ras GTPase-activating-like protein IQGAP1 OS=Homo sapiens GN=IQGAP1 PE=1 SV=1

Match to Query 769: 1542.065448 from(772.040000,2+) intensity(277340.0000) index(396)


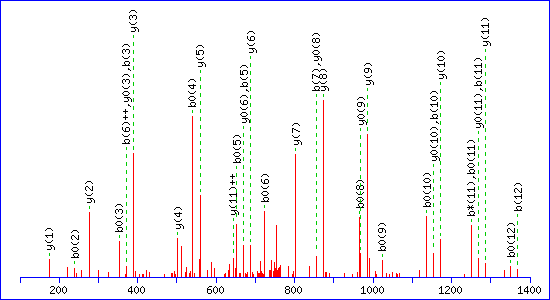


**Monoisotopic mass of neutral peptide Mr(calc):** 1541.8202

**Matches :** 36/140 fragment ions using 36 most intense peaks

| **#** | **b** | **b^++^** | **b*** | **b*^++^** | **b^0^** | **b^0++^** | **Seq.** | **y** | **y^++^** | **y*** | **y*^++^** | **y^0^** | **y^0++^** | **#** |
| --- | --- | --- | --- | --- | --- | --- | --- | --- | --- | --- | --- | --- | --- | --- |
| **1** | 130.0499 | 65.5286 |  |  | 112.0393 | 56.5233 | **E** |  |  |  |  |  |  | **13** |
| **2** | 258.1084 | 129.5579 | 241.0819 | 121.0446 | 240.0979 | 120.5526 | **Q** | 1413.7849 | 707.3961 | 1396.7583 | 698.8828 | 1395.7743 | 698.3908 | **12** |
| **3** | **371.1925** | 186.0999 | 354.1660 | 177.5866 | 353.1819 | 177.0946 | **L** | ***1285.7263*** | 643.3668 | 1268.6997 | 634.8535 | 1267.7157 | 634.3615 | **11** |
| **4** | 557.2718 | 279.1396 | 540.2453 | 270.6263 | 539.2613 | 270.1343 | **W** | ***1172.6422*** | 586.8248 | 1155.6157 | 578.3115 | 1154.6317 | 577.8195 | **10** |
| **5** | **670.3559** | 335.6816 | 653.3293 | 327.1683 | 652.3453 | 326.6763 | **L** | ***986.5629*** | 493.7851 | 969.5364 | 485.2718 | 968.5524 | 484.7798 | **9** |
| **6** | 741.3930 | 371.2001 | 724.3665 | 362.6869 | 723.3824 | 362.1949 | **A** | ***873.4789*** | 437.2431 | 856.4523 | 428.7298 | 855.4683 | 428.2378 | **8** |
| **7** | **855.4359** | 428.2216 | 838.4094 | 419.7083 | 837.4254 | 419.2163 | **N** | ***802.4417*** | 401.7245 | 785.4152 | 393.2112 | 784.4312 | 392.7192 | **7** |
| **8** | 984.4785 | 492.7429 | 967.4520 | 484.2296 | 966.4680 | 483.7376 | **E** | ***688.3988*** | 344.7030 | 671.3723 | 336.1898 | 670.3883 | 335.6978 | **6** |
| **9** | 1041.5000 | 521.2536 | 1024.4734 | 512.7404 | 1023.4894 | 512.2483 | **G** | ***559.3562*** | 280.1817 | 542.3297 | 271.6685 | 541.3457 | 271.1765 | **5** |
| **10** | **1154.5840** | 577.7957 | 1137.5575 | 569.2824 | 1136.5735 | 568.7904 | **L** | ***502.3348*** | 251.6710 | 485.3082 | 243.1577 | 484.3242 | 242.6657 | **4** |
| **11** | **1267.6681** | 634.3377 | 1250.6416 | 625.8244 | 1249.6575 | 625.3324 | **I** | ***389.2507*** | 195.1290 | 372.2241 | 186.6157 | 371.2401 | 186.1237 | **3** |
| **12** | **1368.7158** | 684.8615 | 1351.6892 | 676.3483 | 1350.7052 | 675.8563 | **T** | ***276.1666*** | 138.5870 | 259.1401 | 130.0737 | 258.1561 | 129.5817 | **2** |
| **13** |  |  |  |  |  |  | **R** | ***175.1190*** | 88.0631 | 158.0924 | 79.5498 |  |  | **1** |

MS/MS Fragmentation of **TLINAEDPPMVVVR**
Found in **IQGA1_HUMAN**, Ras GTPase-activating-like protein IQGAP1 OS=Homo sapiens GN=IQGAP1 PE=1 SV=1

Match to Query 770: 1569.045448 from(785.530000,2+) intensity(236846.0000) index(342)


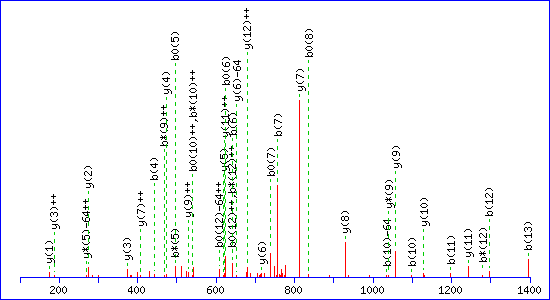


**Monoisotopic mass of neutral peptide Mr(calc):** 1568.8232

**Variable modifications:**

**M10 :** Oxidation (M), with neutral losses 0.0000(shown in table), 63.9983

**Matches :** 39/208 fragment ions using 63 most intense peaks

| **#** | **b** | **b^++^** | **b*** | **b*^++^** | **b^0^** | **b^0++^** | **Seq.** | **y** | **y^++^** | **y*** | **y*^++^** | **y^0^** | **y^0++^** | **#** |
| --- | --- | --- | --- | --- | --- | --- | --- | --- | --- | --- | --- | --- | --- | --- |
| **1** | 102.0550 | 51.5311 |  |  | 84.0444 | 42.5258 | **T** |  |  |  |  |  |  | **14** |
| **2** | 215.1390 | 108.0731 |  |  | 197.1285 | 99.0679 | **L** | 1468.7828 | 734.8951 | 1451.7563 | 726.3818 | 1450.7723 | 725.8898 | **13** |
| **3** | 328.2231 | 164.6152 |  |  | 310.2125 | 155.6099 | **I** | 1355.6988 | 678.3530 | 1338.6722 | 669.8397 | 1337.6882 | 669.3477 | **12** |
| **4** | **442.2660** | 221.6366 | 425.2395 | 213.1234 | 424.2554 | 212.6314 | **N** | ***1242.6147*** | 621.8110 | 1225.5882 | 613.2977 | 1224.6041 | 612.8057 | **11** |
| **5** | 513.3031 | 257.1552 | 496.2766 | 248.6419 | 495.2926 | 248.1499 | **A** | ***1128.5718*** | 564.7895 | 1111.5452 | 556.2762 | 1110.5612 | 555.7842 | **10** |
| **6** | **642.3457** | 321.6765 | 625.3192 | 313.1632 | 624.3352 | 312.6712 | **E** | ***1057.5347*** | 529.2710 | 1040.5081 | 520.7577 | 1039.5241 | 520.2657 | **9** |
| **7** | **757.3727** | 379.1900 | 740.3461 | 370.6767 | 739.3621 | 370.1847 | **D** | ***928.4921*** | 464.7497 | 911.4655 | 456.2364 | 910.4815 | 455.7444 | **8** |
| **8** | 854.4254 | 427.7164 | 837.3989 | 419.2031 | 836.4149 | 418.7111 | **P** | ***813.4651*** | 407.2362 | 796.4386 | 398.7229 |  |  | **7** |
| **9** | 951.4782 | 476.2427 | 934.4516 | 467.7295 | 933.4676 | 467.2374 | **P** | ***716.4124*** | 358.7098 | 699.3858 | 350.1965 |  |  | **6** |
| **10** | **1098.5136** | 549.7604 | 1081.4870 | 541.2472 | 1080.5030 | 540.7551 | **M** | ***619.3596*** | 310.1834 | 602.3330 | 301.6702 |  |  | **5** |
| **11** | **1197.5820** | 599.2946 | 1180.5555 | 590.7814 | 1179.5714 | 590.2894 | **V** | ***472.3242*** | 236.6657 | 455.2976 | 228.1525 |  |  | **4** |
| **12** | **1296.6504** | 648.8288 | 1279.6239 | 640.3156 | 1278.6399 | 639.8236 | **V** | ***373.2558*** | 187.1315 | 356.2292 | 178.6183 |  |  | **3** |
| **13** | **1395.7188** | 698.3631 | 1378.6923 | 689.8498 | 1377.7083 | 689.3578 | **V** | ***274.1874*** | 137.5973 | 257.1608 | 129.0840 |  |  | **2** |
| **14** |  |  |  |  |  |  | **R** | ***175.1190*** | 88.0631 | 158.0924 | 79.5498 |  |  | **1** |

MS/MS Fragmentation of **QIPAITCIQSQWR**
Found in **IQGA1_HUMAN**, Ras GTPase-activating-like protein IQGAP1 OS=Homo sapiens GN=IQGAP1 PE=1 SV=1

Match to Query 771: 1599.985448 from(801.000000,2+) intensity(147233.0000) index(384)


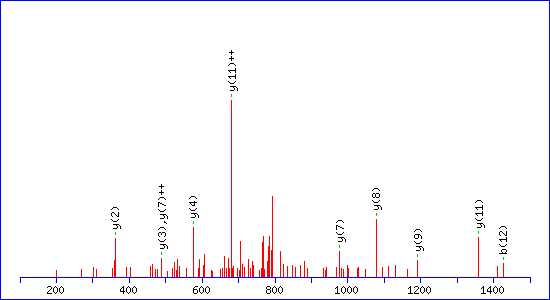


**Monoisotopic mass of neutral peptide Mr(calc):** 1599.8191

**Matches :** 10/128 fragment ions using 12 most intense peaks

| **#** | **b** | **b^++^** | **b*** | **b*^++^** | **b^0^** | **b^0++^** | **Seq.** | **y** | **y^++^** | **y*** | **y*^++^** | **y^0^** | **y^0++^** | **#** |
| --- | --- | --- | --- | --- | --- | --- | --- | --- | --- | --- | --- | --- | --- | --- |
| **1** | 129.0659 | 65.0366 | 112.0393 | 56.5233 |  |  | **Q** |  |  |  |  |  |  | **13** |
| **2** | 242.1499 | 121.5786 | 225.1234 | 113.0653 |  |  | **I** | 1472.7678 | 736.8876 | 1455.7413 | 728.3743 | 1454.7573 | 727.8823 | **12** |
| **3** | 339.2027 | 170.1050 | 322.1761 | 161.5917 |  |  | **P** | ***1359.6838*** | 680.3455 | 1342.6572 | 671.8323 | 1341.6732 | 671.3402 | **11** |
| **4** | 410.2398 | 205.6235 | 393.2132 | 197.1103 |  |  | **A** | 1262.6310 | 631.8191 | 1245.6045 | 623.3059 | 1244.6205 | 622.8139 | **10** |
| **5** | 523.3239 | 262.1656 | 506.2973 | 253.6523 |  |  | **I** | ***1191.5939*** | 596.3006 | 1174.5674 | 587.7873 | 1173.5833 | 587.2953 | **9** |
| **6** | 624.3715 | 312.6894 | 607.3450 | 304.1761 | 606.3610 | 303.6841 | **T** | ***1078.5098*** | 539.7586 | 1061.4833 | 531.2453 | 1060.4993 | 530.7533 | **8** |
| **7** | 784.4022 | 392.7047 | 767.3756 | 384.1915 | 766.3916 | 383.6994 | **C** | ***977.4622*** | 489.2347 | 960.4356 | 480.7214 | 959.4516 | 480.2294 | **7** |
| **8** | 897.4863 | 449.2468 | 880.4597 | 440.7335 | 879.4757 | 440.2415 | **I** | 817.4315 | 409.2194 | 800.4050 | 400.7061 | 799.4209 | 400.2141 | **6** |
| **9** | 1025.5448 | 513.2761 | 1008.5183 | 504.7628 | 1007.5343 | 504.2708 | **Q** | 704.3474 | 352.6774 | 687.3209 | 344.1641 | 686.3369 | 343.6721 | **5** |
| **10** | 1112.5769 | 556.7921 | 1095.5503 | 548.2788 | 1094.5663 | 547.7868 | **S** | ***576.2889*** | 288.6481 | 559.2623 | 280.1348 | 558.2783 | 279.6428 | **4** |
| **11** | 1240.6354 | 620.8214 | 1223.6089 | 612.3081 | 1222.6249 | 611.8161 | **Q** | ***489.2568*** | 245.1321 | 472.2303 | 236.6188 |  |  | **3** |
| **12** | 1426.7147 | 713.8610 | 1409.6882 | 705.3477 | 1408.7042 | 704.8557 | **W** | ***361.1983*** | 181.1028 | 344.1717 | 172.5895 |  |  | **2** |
| **13** |  |  |  |  |  |  | **R** | 175.1190 | 88.0631 | 158.0924 | 79.5498 |  |  | **1** |

MS/MS Fragmentation of **VDQIQEIVTGNPTVIK**
Found in **IQGA1_HUMAN**, Ras GTPase-activating-like protein IQGAP1 OS=Homo sapiens GN=IQGAP1 PE=1 SV=1

Match to Query 780: 1753.265448 from(877.640000,2+) intensity(340608.0000) index(366)


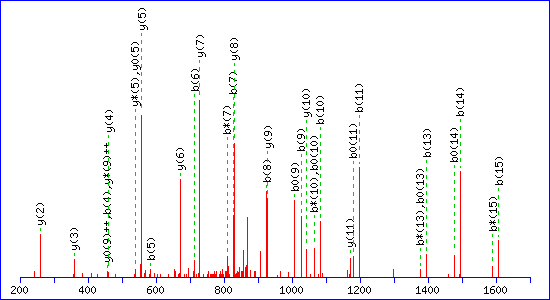


**Monoisotopic mass of neutral peptide Mr(calc):** 1752.9622

**Matches :** 34/168 fragment ions using 37 most intense peaks

| **#** | **b** | **b^++^** | **b*** | **b*^++^** | **b^0^** | **b^0++^** | **Seq.** | **y** | **y^++^** | **y*** | **y*^++^** | **y^0^** | **y^0++^** | **#** |
| --- | --- | --- | --- | --- | --- | --- | --- | --- | --- | --- | --- | --- | --- | --- |
| **1** | 100.0757 | 50.5415 |  |  |  |  | **V** |  |  |  |  |  |  | **16** |
| **2** | 215.1026 | 108.0550 |  |  | 197.0921 | 99.0497 | **D** | 1654.9010 | 827.9542 | 1637.8745 | 819.4409 | 1636.8905 | 818.9489 | **15** |
| **3** | 343.1612 | 172.0842 | 326.1347 | 163.5710 | 325.1506 | 163.0790 | **Q** | 1539.8741 | 770.4407 | 1522.8475 | 761.9274 | 1521.8635 | 761.4354 | **14** |
| **4** | ***456.2453*** | 228.6263 | 439.2187 | 220.1130 | 438.2347 | 219.6210 | **I** | 1411.8155 | 706.4114 | 1394.7890 | 697.8981 | 1393.8049 | 697.4061 | **13** |
| **5** | ***584.3039*** | 292.6556 | 567.2773 | 284.1423 | 566.2933 | 283.6503 | **Q** | 1298.7314 | 649.8694 | 1281.7049 | 641.3561 | 1280.7209 | 640.8641 | **12** |
| **6** | ***713.3464*** | 357.1769 | 696.3199 | 348.6636 | 695.3359 | 348.1716 | **E** | ***1170.6729*** | 585.8401 | 1153.6463 | 577.3268 | 1152.6623 | 576.8348 | **11** |
| **7** | ***826.4305*** | 413.7189 | 809.4040 | 405.2056 | 808.4199 | 404.7136 | **I** | ***1041.6303*** | 521.3188 | 1024.6037 | 512.8055 | 1023.6197 | 512.3135 | **10** |
| **8** | ***925.4989*** | 463.2531 | 908.4724 | 454.7398 | 907.4884 | 454.2478 | **V** | ***928.5462*** | 464.7767 | 911.5197 | 456.2635 | 910.5356 | 455.7715 | **9** |
| **9** | ***1026.5466*** | 513.7769 | 1009.5201 | 505.2637 | 1008.5360 | 504.7717 | **T** | ***829.4778*** | 415.2425 | 812.4512 | 406.7293 | 811.4672 | 406.2373 | **8** |
| **10** | ***1083.5681*** | 542.2877 | 1066.5415 | 533.7744 | 1065.5575 | 533.2824 | **G** | ***728.4301*** | 364.7187 | 711.4036 | 356.2054 | 710.4196 | 355.7134 | **7** |
| **11** | ***1197.6110*** | 599.3091 | 1180.5844 | 590.7959 | 1179.6004 | 590.3039 | **N** | ***671.4087*** | 336.2080 | 654.3821 | 327.6947 | 653.3981 | 327.2027 | **6** |
| **12** | 1294.6638 | 647.8355 | 1277.6372 | 639.3222 | 1276.6532 | 638.8302 | **P** | ***557.3657*** | 279.1865 | 540.3392 | 270.6732 | 539.3552 | 270.1812 | **5** |
| **13** | ***1395.7114*** | 698.3594 | 1378.6849 | 689.8461 | 1377.7009 | 689.3541 | **T** | ***460.3130*** | 230.6601 | 443.2864 | 222.1468 | 442.3024 | 221.6548 | **4** |
| **14** | ***1494.7799*** | 747.8936 | 1477.7533 | 739.3803 | 1476.7693 | 738.8883 | **V** | ***359.2653*** | 180.1363 | 342.2387 | 171.6230 |  |  | **3** |
| **15** | ***1607.8639*** | 804.4356 | 1590.8374 | 795.9223 | 1589.8533 | 795.4303 | **I** | ***260.1969*** | 130.6021 | 243.1703 | 122.0888 |  |  | **2** |
| **16** |  |  |  |  |  |  | **K** | 147.1128 | 74.0600 | 130.0863 | 65.5468 |  |  | **1** |

MS/MS Fragmentation of **ILAIGLINEALDEGDAQK**
Found in **IQGA1_HUMAN**, Ras GTPase-activating-like protein IQGAP1 OS=Homo sapiens GN=IQGAP1 PE=1 SV=1Match to Query 785: 1882.165448 from(942.090000,2+) intensity(265263.0000) index(440)


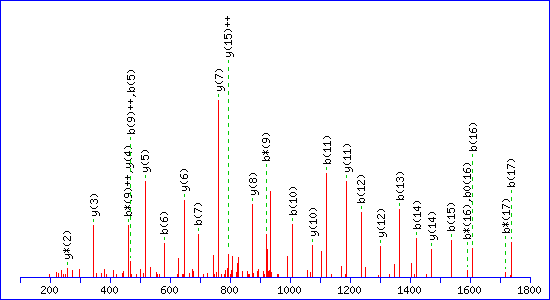


**Monoisotopic mass of neutral peptide Mr(calc):** 1882.0047

**Matches :** 29/168 fragment ions using 31 most intense peaks

| **#** | **b** | **b^++^** | **b*** | **b*^++^** | **b^0^** | **b^0++^** | **Seq.** | **y** | **y^++^** | **y*** | **y*^++^** | **y^0^** | **y^0++^** | **#** |
| --- | --- | --- | --- | --- | --- | --- | --- | --- | --- | --- | --- | --- | --- | --- |
| **1** | 114.0913 | 57.5493 |  |  |  |  | **I** |  |  |  |  |  |  | **18** |
| **2** | 227.1754 | 114.0913 |  |  |  |  | **L** | 1769.9280 | 885.4676 | 1752.9014 | 876.9543 | 1751.9174 | 876.4623 | **17** |
| **3** | 298.2125 | 149.6099 |  |  |  |  | **A** | 1656.8439 | 828.9256 | 1639.8174 | 820.4123 | 1638.8333 | 819.9203 | **16** |
| **4** | 411.2966 | 206.1519 |  |  |  |  | **I** | 1585.8068 | 793.4070 | 1568.7802 | 784.8938 | 1567.7962 | 784.4018 | **15** |
| **5** | ***468.3180*** | 234.6627 |  |  |  |  | **G** | ***1472.7227*** | 736.8650 | 1455.6962 | 728.3517 | 1454.7122 | 727.8597 | **14** |
| **6** | ***581.4021*** | 291.2047 |  |  |  |  | **L** | 1415.7013 | 708.3543 | 1398.6747 | 699.8410 | 1397.6907 | 699.3490 | **13** |
| **7** | ***694.4862*** | 347.7467 |  |  |  |  | **I** | ***1302.6172*** | 651.8122 | 1285.5907 | 643.2990 | 1284.6066 | 642.8070 | **12** |
| **8** | 808.5291 | 404.7682 | 791.5026 | 396.2549 |  |  | **N** | ***1189.5331*** | 595.2702 | 1172.5066 | 586.7569 | 1171.5226 | 586.2649 | **11** |
| **9** | 937.5717 | 469.2895 | 920.5451 | 460.7762 | 919.5611 | 460.2842 | **E** | ***1075.4902*** | 538.2487 | 1058.4637 | 529.7355 | 1057.4796 | 529.2435 | **10** |
| **10** | ***1008.6088*** | 504.8080 | 991.5823 | 496.2948 | 990.5982 | 495.8028 | **A** | 946.4476 | 473.7274 | 929.4211 | 465.2142 | 928.4371 | 464.7222 | **9** |
| **11** | ***1121.6929*** | 561.3501 | 1104.6663 | 552.8368 | 1103.6823 | 552.3448 | **L** | ***875.4105*** | 438.2089 | 858.3840 | 429.6956 | 857.3999 | 429.2036 | **8** |
| **12** | ***1236.7198*** | 618.8635 | 1219.6933 | 610.3503 | 1218.7092 | 609.8583 | **D** | ***762.3264*** | 381.6669 | 745.2999 | 373.1536 | 744.3159 | 372.6616 | **7** |
| **13** | ***1365.7624*** | 683.3848 | 1348.7359 | 674.8716 | 1347.7518 | 674.3796 | **E** | ***647.2995*** | 324.1534 | 630.2729 | 315.6401 | 629.2889 | 315.1481 | **6** |
| **14** | ***1422.7839*** | 711.8956 | 1405.7573 | 703.3823 | 1404.7733 | 702.8903 | **G** | ***518.2569*** | 259.6321 | 501.2304 | 251.1188 | 500.2463 | 250.6268 | **5** |
| **15** | ***1537.8108*** | 769.4090 | 1520.7843 | 760.8958 | 1519.8002 | 760.4038 | **D** | ***461.2354*** | 231.1214 | 444.2089 | 222.6081 | 443.2249 | 222.1161 | **4** |
| **16** | ***1608.8479*** | 804.9276 | 1591.8214 | 796.4143 | 1590.8374 | 795.9223 | **A** | ***346.2085*** | 173.6079 | 329.1819 | 165.0946 |  |  | **3** |
| **17** | ***1736.9065*** | 868.9569 | 1719.8800 | 860.4436 | 1718.8959 | 859.9516 | **Q** | 275.1714 | 138.0893 | 258.1448 | 129.5761 |  |  | **2** |
| **18** |  |  |  |  |  |  | **K** | 147.1128 | 74.0600 | 130.0863 | 65.5468 |  |  | **1** |

MS/MS Fragmentation of **FALGIFAINEAVESGDVGK**
Found in **IQGA1_HUMAN**, Ras GTPase-activating-like protein IQGAP1 OS=Homo sapiens GN=IQGAP1 PE=1 SV=1

Match to Query 790: 1936.185448 from(969.100000,2+) intensity(94876.0000) index(445)


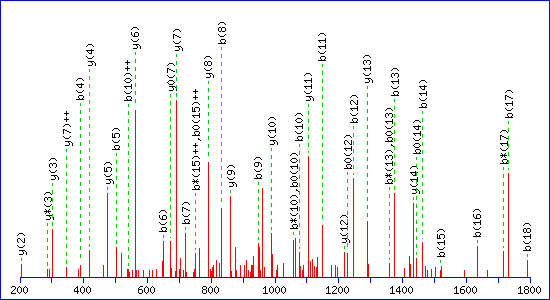


**Monoisotopic mass of neutral peptide Mr(calc):** 1935.9942

**Matches :** 41/176 fragment ions using 57 most intense peaks

| **#** | **b** | **b^++^** | **b*** | **b*^++^** | **b^0^** | **b^0++^** | **Seq.** | **y** | **y^++^** | **y*** | **y*^++^** | **y^0^** | **y^0++^** | **#** |
| --- | --- | --- | --- | --- | --- | --- | --- | --- | --- | --- | --- | --- | --- | --- |
| **1** | 148.0757 | 74.5415 |  |  |  |  | **F** |  |  |  |  |  |  | **19** |
| **2** | 219.1128 | 110.0600 |  |  |  |  | **A** | 1789.9331 | 895.4702 | 1772.9065 | 886.9569 | 1771.9225 | 886.4649 | **18** |
| **3** | 332.1969 | 166.6021 |  |  |  |  | **L** | 1718.8959 | 859.9516 | 1701.8694 | 851.4383 | 1700.8854 | 850.9463 | **17** |
| **4** | ***389.2183*** | 195.1128 |  |  |  |  | **G** | 1605.8119 | 803.4096 | 1588.7853 | 794.8963 | 1587.8013 | 794.4043 | **16** |
| **5** | ***502.3024*** | 251.6548 |  |  |  |  | **I** | 1548.7904 | 774.8988 | 1531.7639 | 766.3856 | 1530.7798 | 765.8936 | **15** |
| **6** | ***649.3708*** | 325.1890 |  |  |  |  | **F** | **1435.7064** | 718.3568 | 1418.6798 | 709.8435 | 1417.6958 | 709.3515 | **14** |
| **7** | ***720.4079*** | 360.7076 |  |  |  |  | **A** | **1288.6379** | 644.8226 | 1271.6114 | 636.3093 | 1270.6274 | 635.8173 | **13** |
| **8** | ***833.4920*** | 417.2496 |  |  |  |  | **I** | **1217.6008** | 609.3040 | 1200.5743 | 600.7908 | 1199.5903 | 600.2988 | **12** |
| **9** | ***947.5349*** | 474.2711 | 930.5084 | 465.7578 |  |  | **N** | **1104.5168** | 552.7620 | 1087.4902 | 544.2487 | 1086.5062 | 543.7567 | **11** |
| **10** | ***1076.5775*** | 538.7924 | 1059.5510 | 530.2791 | 1058.5669 | 529.7871 | **E** | **990.4738** | 495.7406 | 973.4473 | 487.2273 | 972.4633 | 486.7353 | **10** |
| **11** | ***1147.6146*** | 574.3109 | 1130.5881 | 565.7977 | 1129.6041 | 565.3057 | **A** | **861.4312** | 431.2193 | 844.4047 | 422.7060 | 843.4207 | 422.2140 | **9** |
| **12** | ***1246.6830*** | 623.8452 | 1229.6565 | 615.3319 | 1228.6725 | 614.8399 | **V** | **790.3941** | 395.7007 | 773.3676 | 387.1874 | 772.3836 | 386.6954 | **8** |
| **13** | ***1375.7256*** | 688.3665 | 1358.6991 | 679.8532 | 1357.7151 | 679.3612 | **E** | **691.3257** | 346.1665 | 674.2992 | 337.6532 | 673.3151 | 337.1612 | **7** |
| **14** | ***1462.7577*** | 731.8825 | 1445.7311 | 723.3692 | 1444.7471 | 722.8772 | **S** | **562.2831** | 281.6452 | 545.2566 | 273.1319 | 544.2726 | 272.6399 | **6** |
| **15** | ***1519.7791*** | 760.3932 | 1502.7526 | 751.8799 | 1501.7686 | 751.3879 | **G** | **475.2511** | 238.1292 | 458.2245 | 229.6159 | 457.2405 | 229.1239 | **5** |
| **16** | ***1634.8061*** | 817.9067 | 1617.7795 | 809.3934 | 1616.7955 | 808.9014 | **D** | **418.2296** | 209.6185 | 401.2031 | 201.1052 | 400.2191 | 200.6132 | **4** |
| **17** | ***1733.8745*** | 867.4409 | 1716.8479 | 858.9276 | 1715.8639 | 858.4356 | **V** | **303.2027** | 152.1050 | 286.1761 | 143.5917 |  |  | **3** |
| **18** | ***1790.8959*** | 895.9516 | 1773.8694 | 887.4383 | 1772.8854 | 886.9463 | **G** | **204.1343** | 102.5708 | 187.1077 | 94.0575 |  |  | **2** |
| **19** |  |  |  |  |  |  | **K** | 147.1128 | 74.0600 | 130.0863 | 65.5468 |  |  | **1** |

MS/MS Fragmentation of **NVIFEISPTEEVGDFEVK**
Found in **IQGA1_HUMAN**, Ras GTPase-activating-like protein IQGAP1 OS=Homo sapiens GN=IQGAP1 PE=1 SV=1

Match to Query 799: 2051.125448 from(1026.570000,2+) intensity(142043.0000) index(412)


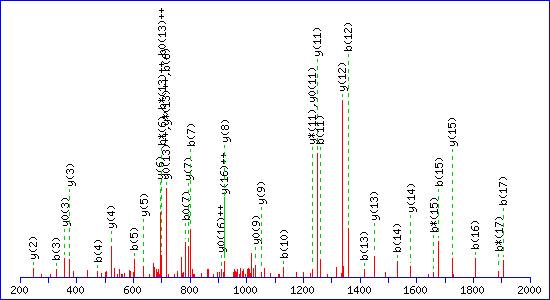


**Monoisotopic mass of neutral peptide Mr(calc):** 2051.0099

**Matches :** 40/192 fragment ions using 46 most intense peaks

| **#** | **b** | **b^++^** | **b*** | **b*^++^** | **b^0^** | **b^0++^** | **Seq.** | **y** | **y^++^** | **y*** | **y*^++^** | **y^0^** | **y^0++^** | **#** |
| --- | --- | --- | --- | --- | --- | --- | --- | --- | --- | --- | --- | --- | --- | --- |
| **1** | 115.0502 | 58.0287 | 98.0237 | 49.5155 |  |  | **N** |  |  |  |  |  |  | **18** |
| **2** | 214.1186 | 107.5629 | 197.0921 | 99.0497 |  |  | **V** | 1937.9743 | 969.4908 | 1920.9477 | 960.9775 | 1919.9637 | 960.4855 | **17** |
| **3** | ***327.2027*** | 164.1050 | 310.1761 | 155.5917 |  |  | **I** | 1838.9058 | 919.9566 | 1821.8793 | 911.4433 | 1820.8953 | 910.9513 | **16** |
| **4** | ***474.2711*** | 237.6392 | 457.2445 | 229.1259 |  |  | **F** | ***1725.8218*** | 863.4145 | 1708.7952 | 854.9013 | 1707.8112 | 854.4092 | **15** |
| **5** | ***603.3137*** | 302.1605 | 586.2871 | 293.6472 | 585.3031 | 293.1552 | **E** | ***1578.7534*** | 789.8803 | 1561.7268 | 781.3670 | 1560.7428 | 780.8750 | **14** |
| **6** | ***716.3978*** | 358.7025 | 699.3712 | 350.1892 | 698.3872 | 349.6972 | **I** | ***1449.7108*** | 725.3590 | 1432.6842 | 716.8457 | 1431.7002 | 716.3537 | **13** |
| **7** | ***803.4298*** | 402.2185 | 786.4032 | 393.7053 | 785.4192 | 393.2132 | **S** | ***1336.6267*** | 668.8170 | 1319.6002 | 660.3037 | 1318.6161 | 659.8117 | **12** |
| **8** | 900.4825 | 450.7449 | 883.4560 | 442.2316 | 882.4720 | 441.7396 | **P** | ***1249.5947*** | 625.3010 | 1232.5681 | 616.7877 | 1231.5841 | 616.2957 | **11** |
| **9** | 1001.5302 | 501.2687 | 984.5037 | 492.7555 | 983.5197 | 492.2635 | **T** | 1152.5419 | 576.7746 | 1135.5154 | 568.2613 | 1134.5313 | 567.7693 | **10** |
| **10** | ***1130.5728*** | 565.7900 | 1113.5463 | 557.2768 | 1112.5623 | 556.7848 | **E** | ***1051.4942*** | 526.2508 | 1034.4677 | 517.7375 | 1033.4837 | 517.2455 | **9** |
| **11** | ***1259.6154*** | 630.3113 | 1242.5889 | 621.7981 | 1241.6048 | 621.3061 | **E** | ***922.4516*** | 461.7295 | 905.4251 | 453.2162 | 904.4411 | 452.7242 | **8** |
| **12** | ***1358.6838*** | 679.8455 | 1341.6573 | 671.3323 | 1340.6733 | 670.8403 | **V** | ***793.4090*** | 397.2082 | 776.3825 | 388.6949 | 775.3985 | 388.2029 | **7** |
| **13** | ***1415.7053*** | 708.3563 | 1398.6787 | 699.8430 | 1397.6947 | 699.3510 | **G** | ***694.3406*** | 347.6740 | 677.3141 | 339.1607 | 676.3301 | 338.6687 | **6** |
| **14** | ***1530.7322*** | 765.8698 | 1513.7057 | 757.3565 | 1512.7217 | 756.8645 | **D** | ***637.3192*** | 319.1632 | 620.2926 | 310.6499 | 619.3086 | 310.1579 | **5** |
| **15** | ***1677.8006*** | 839.4040 | 1660.7741 | 830.8907 | 1659.7901 | 830.3987 | **F** | ***522.2922*** | 261.6498 | 505.2657 | 253.1365 | 504.2817 | 252.6445 | **4** |
| **16** | ***1806.8432*** | 903.9253 | 1789.8167 | 895.4120 | 1788.8327 | 894.9200 | **E** | ***375.2238*** | 188.1155 | 358.1973 | 179.6023 | 357.2132 | 179.1103 | **3** |
| **17** | ***1905.9117*** | 953.4595 | 1888.8851 | 944.9462 | 1887.9011 | 944.4542 | **V** | ***246.1812*** | 123.5942 | 229.1547 | 115.0810 |  |  | **2** |
| **18** |  |  |  |  |  |  | **K** | 147.1128 | 74.0600 | 130.0863 | 65.5468 |  |  | **1** |

MS/MS Fragmentation of **LEGVLAEVAQHYQDTLIR**
Found in **IQGA1_HUMAN**, Ras GTPase-activating-like protein IQGAP1 OS=Homo sapiens GN=IQGAP1 PE=1 SV=1

Match to Query 800: 2054.468172 from(685.830000,3+) intensity(129040.0000) index(414)


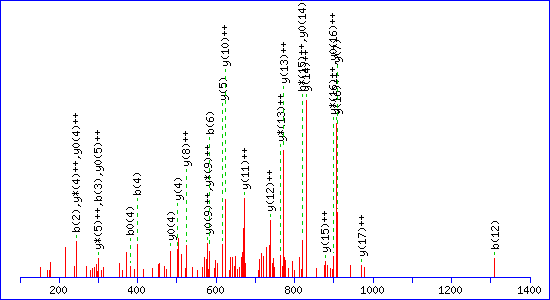


**Monoisotopic mass of neutral peptide Mr(calc):** 2054.0796

**Matches :** 31/178 fragment ions using 35 most intense peaks

| **#** | **b** | **b^++^** | **b*** | **b*^++^** | **b^0^** | **b^0++^** | **Seq.** | **y** | **y^++^** | **y*** | **y*^++^** | **y^0^** | **y^0++^** | **#** |
| --- | --- | --- | --- | --- | --- | --- | --- | --- | --- | --- | --- | --- | --- | --- |
| **1** | 114.0913 | 57.5493 |  |  |  |  | **L** |  |  |  |  |  |  | **18** |
| **2** | **243.1339** | 122.0706 |  |  | 225.1234 | 113.0653 | **E** | 1942.0029 | 971.5051 | 1924.9763 | 962.9918 | 1923.9923 | 962.4998 | **17** |
| **3** | **300.1554** | 150.5813 |  |  | 282.1448 | 141.5761 | **G** | 1812.9603 | 906.9838 | 1795.9337 | 898.4705 | 1794.9497 | 897.9785 | **16** |
| **4** | **399.2238** | 200.1155 |  |  | 381.2132 | 191.1103 | **V** | 1755.9388 | 878.4730 | 1738.9123 | 869.9598 | 1737.9282 | 869.4678 | **15** |
| **5** | 512.3079 | 256.6576 |  |  | 494.2973 | 247.6523 | **L** | 1656.8704 | 828.9388 | 1639.8438 | 820.4256 | 1638.8598 | 819.9336 | **14** |
| **6** | **583.3450** | 292.1761 |  |  | 565.3344 | 283.1709 | **A** | 1543.7863 | 772.3968 | 1526.7598 | 763.8835 | 1525.7758 | 763.3915 | **13** |
| **7** | 712.3876 | 356.6974 |  |  | 694.3770 | 347.6921 | **E** | 1472.7492 | 736.8782 | 1455.7227 | 728.3650 | 1454.7387 | 727.8730 | **12** |
| **8** | 811.4560 | 406.2316 |  |  | 793.4454 | 397.2264 | **V** | 1343.7066 | 672.3570 | 1326.6801 | 663.8437 | 1325.6961 | 663.3517 | **11** |
| **9** | 882.4931 | 441.7502 |  |  | 864.4825 | 432.7449 | **A** | 1244.6382 | 622.8227 | 1227.6117 | 614.3095 | 1226.6276 | 613.8175 | **10** |
| **10** | 1010.5517 | 505.7795 | 993.5251 | 497.2662 | 992.5411 | 496.7742 | **Q** | 1173.6011 | 587.3042 | 1156.5745 | 578.7909 | 1155.5905 | 578.2989 | **9** |
| **11** | 1147.6106 | 574.3089 | 1130.5841 | 565.7957 | 1129.6000 | 565.3037 | **H** | 1045.5425 | 523.2749 | 1028.5160 | 514.7616 | 1027.5320 | 514.2696 | **8** |
| **12** | **1310.6739** | 655.8406 | 1293.6474 | 647.3273 | 1292.6634 | 646.8353 | **Y** | **908.4836** | 454.7454 | 891.4571 | 446.2322 | 890.4730 | 445.7402 | **7** |
| **13** | 1438.7325 | 719.8699 | 1421.7060 | 711.3566 | 1420.7219 | 710.8646 | **Q** | 745.4203 | 373.2138 | 728.3937 | 364.7005 | 727.4097 | 364.2085 | **6** |
| **14** | 1553.7595 | 777.3834 | 1536.7329 | 768.8701 | 1535.7489 | 768.3781 | **D** | **617.3617** | 309.1845 | 600.3352 | 300.6712 | 599.3511 | 300.1792 | **5** |
| **15** | 1654.8071 | 827.9072 | 1637.7806 | 819.3939 | 1636.7966 | 818.9019 | **T** | **502.3348** | 251.6710 | 485.3082 | 243.1577 | 484.3242 | 242.6657 | **4** |
| **16** | 1767.8912 | 884.4492 | 1750.8646 | 875.9360 | 1749.8806 | 875.4440 | **L** | 401.2871 | 201.1472 | 384.2605 | 192.6339 |  |  | **3** |
| **17** | 1880.9753 | 940.9913 | 1863.9487 | 932.4780 | 1862.9647 | 931.9860 | **I** | 288.2030 | 144.6051 | 271.1765 | 136.0919 |  |  | **2** |
| **18** |  |  |  |  |  |  | **R** | 175.1190 | 88.0631 | 158.0924 | 79.5498 |  |  | **1** |

MS/MS Fragmentation of **VDFTEEEINNMKTELEK**
Found in **IQGA1_HUMAN**, Ras GTPase-activating-like protein IQGAP1 OS=Homo sapiens GN=IQGAP1 PE=1 SV=1

Match to Query 802: 2084.138172 from(695.720000,3+) intensity(127284.0000) index(326)


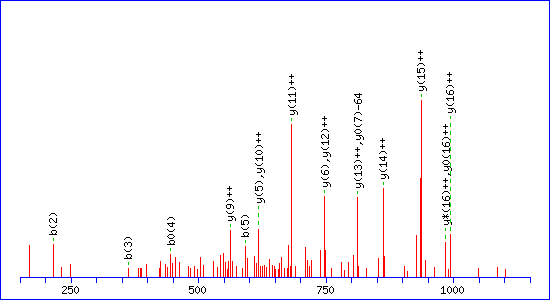


**Monoisotopic mass of neutral peptide Mr(calc):** 2083.9619

**Variable modifications:**

**M11 :** Oxidation (M), with neutral losses 0.0000(shown in table), 63.9983

**Matches :** 17/268 fragment ions using 19 most intense peaks

| **#** | **b** | **b^++^** | **b*** | **b*^++^** | **b^0^** | **b^0++^** | **Seq.** | **y** | **y^++^** | **y*** | **y*^++^** | **y^0^** | **y^0++^** | **#** |
| --- | --- | --- | --- | --- | --- | --- | --- | --- | --- | --- | --- | --- | --- | --- |
| **1** | 100.0757 | 50.5415 |  |  |  |  | **V** |  |  |  |  |  |  | **17** |
| **2** | **215.1026** | 108.0550 |  |  | 197.0921 | 99.0497 | **D** | 1985.9008 | 993.4541 | 1968.8743 | 984.9408 | 1967.8903 | 984.4488 | **16** |
| **3** | **362.1710** | 181.5892 |  |  | 344.1605 | 172.5839 | **F** | 1870.8739 | 935.9406 | 1853.8473 | 927.4273 | 1852.8633 | 926.9353 | **15** |
| **4** | 463.2187 | 232.1130 |  |  | 445.2082 | 223.1077 | **T** | 1723.8055 | 862.4064 | 1706.7789 | 853.8931 | 1705.7949 | 853.4011 | **14** |
| **5** | **592.2613** | 296.6343 |  |  | 574.2508 | 287.6290 | **E** | 1622.7578 | 811.8825 | 1605.7312 | 803.3693 | 1604.7472 | 802.8773 | **13** |
| **6** | 721.3039 | 361.1556 |  |  | 703.2933 | 352.1503 | **E** | 1493.7152 | 747.3612 | 1476.6887 | 738.8480 | 1475.7046 | 738.3560 | **12** |
| **7** | 850.3465 | 425.6769 |  |  | 832.3359 | 416.6716 | **E** | 1364.6726 | 682.8399 | 1347.6461 | 674.3267 | 1346.6620 | 673.8347 | **11** |
| **8** | 963.4306 | 482.2189 |  |  | 945.4200 | 473.2136 | **I** | 1235.6300 | 618.3186 | 1218.6035 | 609.8054 | 1217.6194 | 609.3134 | **10** |
| **9** | 1077.4735 | 539.2404 | 1060.4469 | 530.7271 | 1059.4629 | 530.2351 | **N** | 1122.5459 | 561.7766 | 1105.5194 | 553.2633 | 1104.5354 | 552.7713 | **9** |
| **10** | 1191.5164 | 596.2618 | 1174.4899 | 587.7486 | 1173.5059 | 587.2566 | **N** | 1008.5030 | 504.7551 | 991.4765 | 496.2419 | 990.4925 | 495.7499 | **8** |
| **11** | 1338.5518 | 669.7795 | 1321.5253 | 661.2663 | 1320.5413 | 660.7743 | **M** | 894.4601 | 447.7337 | 877.4335 | 439.2204 | 876.4495 | 438.7284 | **7** |
| **12** | 1466.6468 | 733.8270 | 1449.6202 | 725.3138 | 1448.6362 | 724.8217 | **K** | **747.4247** | 374.2160 | 730.3981 | 365.7027 | 729.4141 | 365.2107 | **6** |
| **13** | 1567.6945 | 784.3509 | 1550.6679 | 775.8376 | 1549.6839 | 775.3456 | **T** | **619.3297** | 310.1685 | 602.3032 | 301.6552 | 601.3192 | 301.1632 | **5** |
| **14** | 1696.7371 | 848.8722 | 1679.7105 | 840.3589 | 1678.7265 | 839.8669 | **E** | 518.2821 | 259.6447 | 501.2555 | 251.1314 | 500.2715 | 250.6394 | **4** |
| **15** | 1809.8211 | 905.4142 | 1792.7946 | 896.9009 | 1791.8106 | 896.4089 | **L** | 389.2395 | 195.1234 | 372.2129 | 186.6101 | 371.2289 | 186.1181 | **3** |
| **16** | 1938.8637 | 969.9355 | 1921.8372 | 961.4222 | 1920.8531 | 960.9302 | **E** | 276.1554 | 138.5813 | 259.1288 | 130.0681 | 258.1448 | 129.5761 | **2** |
| **17** |  |  |  |  |  |  | **K** | 147.1128 | 74.0600 | 130.0863 | 65.5468 |  |  | **1** |

MS/MS Fragmentation of **SAADEVDGLGVARPHYGSVLDNER**
Found in **IQGA1_HUMAN**, Ras GTPase-activating-like protein IQGAP1 OS=Homo sapiens GN=IQGAP1 PE=1 SV=1

Match to Query 837: 2568.428172 from(857.150000,3+) intensity(330711.0000) index(351)

**Monoisotopic mass of neutral peptide Mr(calc):** 2568.2204


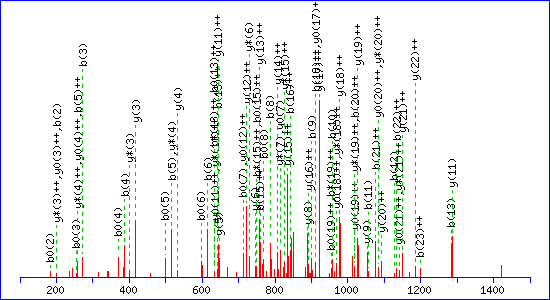


**Variable modifications:**

**N-term :** Acetyl (N-term)

**Matches :** 75/250 fragment ions using 82 most intense peaks

| **#** | **b** | **b^++^** | **b*** | **b*^++^** | **b^0^** | **b^0++^** | **Seq.** | **y** | **y^++^** | **y*** | **y*^++^** | **y^0^** | **y^0++^** | **#** |
| --- | --- | --- | --- | --- | --- | --- | --- | --- | --- | --- | --- | --- | --- | --- |
| **1** | 130.0499 | 65.5286 |  |  | 112.0393 | 56.5233 | **S** |  |  |  |  |  |  | **24** |
| **2** | **201.0870** | 101.0471 |  |  | 183.0764 | 92.0418 | **A** | 2440.1851 | 1220.5962 | 2423.1586 | 1212.0829 | 2422.1746 | 1211.5909 | **23** |
| **3** | **272.1241** | 136.5657 |  |  | 254.1135 | 127.5604 | **A** | 2369.1480 | 1185.0777 | 2352.1215 | 1176.5644 | 2351.1375 | 1176.0724 | **22** |
| **4** | **387.1510** | 194.0792 |  |  | 369.1405 | 185.0739 | **D** | 2298.1109 | 1149.5591 | 2281.0844 | 1141.0458 | 2280.1003 | 1140.5538 | **21** |
| **5** | **516.1936** | ***258.6005*** |  |  | 498.1831 | 249.5952 | **E** | 2183.0840 | 1092.0456 | 2166.0574 | 1083.5323 | 2165.0734 | 1083.0403 | **20** |
| **6** | **615.2620** | 308.1347 |  |  | 597.2515 | 299.1294 | **V** | 2054.0414 | 1027.5243 | 2037.0148 | 1019.0111 | 2036.0308 | 1018.5190 | **19** |
| **7** | 730.2890 | 365.6481 |  |  | 712.2784 | 356.6429 | **D** | 1954.9730 | 977.9901 | 1937.9464 | 969.4768 | 1936.9624 | 968.9848 | **18** |
| **8** | **787.3105** | 394.1589 |  |  | 769.2999 | 385.1536 | **G** | 1839.9460 | 920.4766 | 1822.9195 | 911.9634 | 1821.9355 | 911.4714 | **17** |
| **9** | **900.3945** | 450.7009 |  |  | 882.3840 | 441.6956 | **L** | 1782.9246 | 891.9659 | 1765.8980 | 883.4526 | 1764.9140 | 882.9606 | **16** |
| **10** | **957.4160** | 479.2116 |  |  | 939.4054 | 470.2063 | **G** | 1669.8405 | 835.4239 | 1652.8139 | 826.9106 | 1651.8299 | 826.4186 | **15** |
| **11** | **1056.4844** | 528.7458 |  |  | 1038.4738 | 519.7406 | **V** | 1612.8190 | 806.9132 | 1595.7925 | 798.3999 | 1594.8085 | 797.9079 | **14** |
| **12** | **1127.5215** | 564.2644 |  |  | 1109.5109 | 555.2591 | **A** | 1513.7506 | 757.3789 | 1496.7241 | 748.8657 | 1495.7401 | 748.3737 | **13** |
| **13** | **1283.6226** | ***642.3149*** | 1266.5961 | 633.8017 | 1265.6121 | 633.3097 | **R** | 1442.7135 | 721.8604 | 1425.6870 | 713.3471 | 1424.7029 | 712.8551 | **12** |
| **14** | 1380.6754 | 690.8413 | 1363.6488 | 682.3281 | 1362.6648 | 681.8360 | **P** | **1286.6124** | 643.8098 | 1269.5858 | 635.2966 | 1268.6018 | 634.8046 | **11** |
| **15** | 1517.7343 | ***759.3708*** | 1500.7077 | 750.8575 | 1499.7237 | 750.3655 | **H** | 1189.5596 | 595.2835 | 1172.5331 | 586.7702 | 1171.5491 | 586.2782 | **10** |
| **16** | 1680.7976 | ***840.9025*** | 1663.7711 | 832.3892 | 1662.7871 | 831.8972 | **Y** | **1052.5007** | 526.7540 | 1035.4742 | 518.2407 | 1034.4901 | 517.7487 | **9** |
| **17** | 1737.8191 | 869.4132 | 1720.7925 | 860.8999 | 1719.8085 | 860.4079 | **G** | **889.4374** | 445.2223 | 872.4108 | 436.7091 | 871.4268 | 436.2170 | **8** |
| **18** | 1824.8511 | ***912.9292*** | 1807.8246 | 904.4159 | 1806.8406 | 903.9239 | **S** | 832.4159 | 416.7116 | 815.3894 | 408.1983 | 814.4054 | 407.7063 | **7** |
| **19** | 1923.9195 | 962.4634 | 1906.8930 | 953.9501 | 1905.9090 | 953.4581 | **V** | **745.3839** | 373.1956 | 728.3573 | 364.6823 | 727.3733 | 364.1903 | **6** |
| **20** | 2037.0036 | ***1019.0054*** | 2019.9770 | 1010.4922 | 2018.9930 | 1010.0002 | **L** | **646.3155** | 323.6614 | 629.2889 | 315.1481 | 628.3049 | 314.6561 | **5** |
| **21** | 2152.0305 | ***1076.5189*** | 2135.0040 | 1068.0056 | 2134.0200 | 1067.5136 | **D** | **533.2314** | 267.1193 | 516.2049 | 258.6061 | 515.2209 | 258.1141 | **4** |
| **22** | 2266.0735 | ***1133.5404*** | 2249.0469 | 1125.0271 | 2248.0629 | 1124.5351 | **N** | **418.2045** | 209.6059 | 401.1779 | 201.0926 | 400.1939 | 200.6006 | **3** |
| **23** | 2395.1161 | ***1198.0617*** | 2378.0895 | 1189.5484 | 2377.1055 | 1189.0564 | **E** | 304.1615 | 152.5844 | 287.1350 | 144.0711 | 286.1510 | 143.5791 | **2** |
| **24** |  |  |  |  |  |  | **R** | 175.1190 | 88.0631 | 158.0924 | 79.5498 |  |  | **1** |

MS/MS Fragmentation of **FQPGETLTEILETPATSEQEAEHQR**
Found in **IQGA1_HUMAN**, Ras GTPase-activating-like protein IQGAP1 OS=Homo sapiens GN=IQGAP1 PE=1 SV=1

Match to Query 848: 2840.468172 from(947.830000,3+) intensity(129002.0000) index(405)


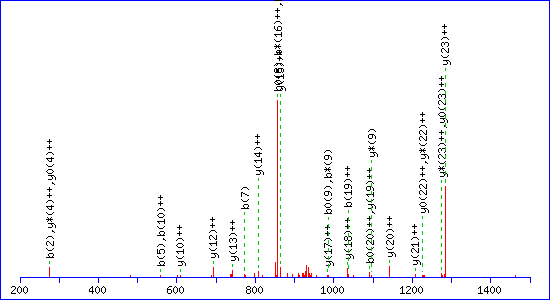


**Monoisotopic mass of neutral peptide Mr(calc):** 2840.3464

**Matches :** 29/272 fragment ions using 36 most intense peaks

| **#** | **b** | **b^++^** | **b*** | **b*^++^** | **b^0^** | **b^0++^** | **Seq.** | **y** | **y^++^** | **y*** | **y*^++^** | **y^0^** | **y^0++^** | **#** |
| --- | --- | --- | --- | --- | --- | --- | --- | --- | --- | --- | --- | --- | --- | --- |
| **1** | 148.0757 | 74.5415 |  |  |  |  | **F** |  |  |  |  |  |  | **25** |
| **2** | **276.1343** | 138.5708 | 259.1077 | 130.0575 |  |  | **Q** | 2694.2853 | 1347.6463 | 2677.2588 | 1339.1330 | 2676.2747 | 1338.6410 | **24** |
| **3** | 373.1870 | 187.0972 | 356.1605 | 178.5839 |  |  | **P** | 2566.2267 | 1283.6170 | 2549.2002 | 1275.1037 | 2548.2162 | 1274.6117 | **23** |
| **4** | 430.2085 | 215.6079 | 413.1819 | 207.0946 |  |  | **G** | 2469.1740 | 1235.0906 | 2452.1474 | 1226.5773 | 2451.1634 | 1226.0853 | **22** |
| **5** | **559.2511** | 280.1292 | 542.2245 | 271.6159 | 541.2405 | 271.1239 | **E** | 2412.1525 | 1206.5799 | 2395.1260 | 1198.0666 | 2394.1419 | 1197.5746 | **21** |
| **6** | 660.2988 | 330.6530 | 643.2722 | 322.1397 | 642.2882 | 321.6477 | **T** | 2283.1099 | 1142.0586 | 2266.0834 | 1133.5453 | 2265.0993 | 1133.0533 | **20** |
| **7** | **773.3828** | 387.1951 | 756.3563 | 378.6818 | 755.3723 | 378.1898 | **L** | 2182.0622 | 1091.5348 | 2165.0357 | 1083.0215 | 2164.0517 | 1082.5295 | **19** |
| **8** | 874.4305 | 437.7189 | 857.4040 | 429.2056 | 856.4199 | 428.7136 | **T** | 2068.9782 | 1034.9927 | 2051.9516 | 1026.4794 | 2050.9676 | 1025.9874 | **18** |
| **9** | 1003.4731 | 502.2402 | 986.4466 | 493.7269 | 985.4625 | 493.2349 | **E** | 1967.9305 | 984.4689 | 1950.9039 | 975.9556 | 1949.9199 | 975.4636 | **17** |
| **10** | 1116.5572 | 558.7822 | 1099.5306 | 550.2689 | 1098.5466 | 549.7769 | **I** | 1838.8879 | 919.9476 | 1821.8613 | 911.4343 | 1820.8773 | 910.9423 | **16** |
| **11** | 1229.6412 | 615.3243 | 1212.6147 | 606.8110 | 1211.6307 | 606.3190 | **L** | 1725.8038 | 863.4056 | 1708.7773 | 854.8923 | 1707.7933 | 854.4003 | **15** |
| **12** | 1358.6838 | 679.8456 | 1341.6573 | 671.3323 | 1340.6733 | 670.8403 | **E** | 1612.7198 | 806.8635 | 1595.6932 | 798.3502 | 1594.7092 | 797.8582 | **14** |
| **13** | 1459.7315 | 730.3694 | 1442.7050 | 721.8561 | 1441.7209 | 721.3641 | **T** | 1483.6772 | 742.3422 | 1466.6506 | 733.8290 | 1465.6666 | 733.3369 | **13** |
| **14** | 1556.7843 | 778.8958 | 1539.7577 | 770.3825 | 1538.7737 | 769.8905 | **P** | 1382.6295 | 691.8184 | 1365.6029 | 683.3051 | 1364.6189 | 682.8131 | **12** |
| **15** | 1627.8214 | 814.4143 | 1610.7948 | 805.9011 | 1609.8108 | 805.4090 | **A** | 1285.5767 | 643.2920 | 1268.5502 | 634.7787 | 1267.5662 | 634.2867 | **11** |
| **16** | 1728.8691 | 864.9382 | 1711.8425 | 856.4249 | 1710.8585 | 855.9329 | **T** | 1214.5396 | 607.7734 | 1197.5131 | 599.2602 | 1196.5291 | 598.7682 | **10** |
| **17** | 1815.9011 | 908.4542 | 1798.8745 | 899.9409 | 1797.8905 | 899.4489 | **S** | 1113.4919 | 557.2496 | 1096.4654 | 548.7363 | 1095.4814 | 548.2443 | **9** |
| **18** | 1944.9437 | 972.9755 | 1927.9171 | 964.4622 | 1926.9331 | 963.9702 | **E** | 1026.4599 | 513.7336 | 1009.4334 | 505.2203 | 1008.4493 | 504.7283 | **8** |
| **19** | 2073.0023 | 1037.0048 | 2055.9757 | 1028.4915 | 2054.9917 | 1027.9995 | **Q** | 897.4173 | 449.2123 | 880.3908 | 440.6990 | 879.4068 | 440.2070 | **7** |
| **20** | 2202.0449 | 1101.5261 | 2185.0183 | 1093.0128 | 2184.0343 | 1092.5208 | **E** | 769.3587 | 385.1830 | 752.3322 | 376.6697 | 751.3482 | 376.1777 | **6** |
| **21** | 2273.0820 | 1137.0446 | 2256.0554 | 1128.5313 | 2255.0714 | 1128.0393 | **A** | 640.3161 | 320.6617 | 623.2896 | 312.1484 | 622.3056 | 311.6564 | **5** |
| **22** | 2402.1246 | 1201.5659 | 2385.0980 | 1193.0526 | 2384.1140 | 1192.5606 | **E** | 569.2790 | 285.1432 | 552.2525 | 276.6299 | 551.2685 | 276.1379 | **4** |
| **23** | 2539.1835 | 1270.0954 | 2522.1569 | 1261.5821 | 2521.1729 | 1261.0901 | **H** | 440.2364 | 220.6219 | 423.2099 | 212.1086 |  |  | **3** |
| **24** | 2667.2421 | 1334.1247 | 2650.2155 | 1325.6114 | 2649.2315 | 1325.1194 | **Q** | 303.1775 | 152.0924 | 286.1510 | 143.5791 |  |  | **2** |
| **25** |  |  |  |  |  |  | **R** | 175.1190 | 88.0631 | 158.0924 | 79.5498 |  |  | **1** |
