# Supplementary material for: The Pseudomonas aeruginosa N-Acylhomoserine Lactone Quorum Sensing Molecules Target IQGAP1 and Modulate Epithelial Cell Migration
Source: PLoS Pathog. 2012 Oct 11;8(10):e1002953. doi: 10.1371/journal.ppat.1002953 (PMC3469656; doi:10.1371/journal.ppat.1002953)
Supplement: Dataset S2 — Peptide identification views from MASCOT MS data analyses of IQGAP2 peptides sequenced by collision-induced dissociation of their ions. The spectra and corresponding lists of fragment ions identified in the MASCOT search are shown. (DOCX) [file ppat.1002953.s002.docx]

MS/MS Fragmentation of **LGDSESVSK**
Found in **IQGA2_HUMAN**, Ras GTPase-activating-like protein IQGAP2 OS=Homo sapiens GN=IQGAP2 PE=1 SV=4


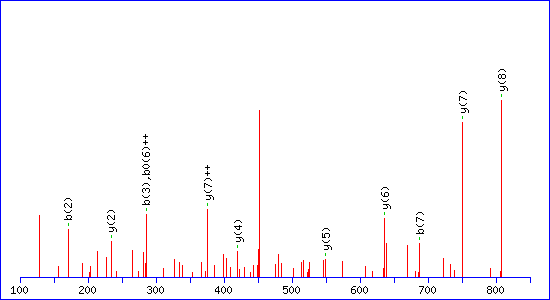
Match to Query 682: 920.685448 from(461.350000,2+) intensity(61163.0000) index(82)

**Monoisotopic mass of neutral peptide Mr(calc):** 920.4451

**Matches :** 11/74 fragment ions using 14 most intense peaks

| **#** | **b** | **b^++^** | **b^0^** | **b^0++^** | **Seq.** | **y** | **y^++^** | **y*** | **y*^++^** | **y^0^** | **y^0++^** | **#** |
| --- | --- | --- | --- | --- | --- | --- | --- | --- | --- | --- | --- | --- |
| **1** | 114.0913 | 57.5493 |  |  | **L** |  |  |  |  |  |  | **9** |
| **2** | **171.1128** | 86.0600 |  |  | **G** | ***808.3683*** | 404.6878 | 791.3418 | 396.1745 | 790.3577 | 395.6825 | **8** |
| **3** | **286.1397** | 143.5735 | 268.1292 | 134.5682 | **D** | ***751.3468*** | 376.1771 | 734.3203 | 367.6638 | 733.3363 | 367.1718 | **7** |
| **4** | 373.1718 | 187.0895 | 355.1612 | 178.0842 | **S** | ***636.3199*** | 318.6636 | 619.2933 | 310.1503 | 618.3093 | 309.6583 | **6** |
| **5** | 502.2144 | 251.6108 | 484.2038 | 242.6055 | **E** | ***549.2879*** | 275.1476 | 532.2613 | 266.6343 | 531.2773 | 266.1423 | **5** |
| **6** | 589.2464 | 295.1268 | 571.2358 | 286.1216 | **S** | ***420.2453*** | 210.6263 | 403.2187 | 202.1130 | 402.2347 | 201.6210 | **4** |
| **7** | **688.3148** | 344.6610 | 670.3042 | 335.6558 | **V** | 333.2132 | 167.1103 | 316.1867 | 158.5970 | 315.2027 | 158.1050 | **3** |
| **8** | 775.3468 | 388.1771 | 757.3363 | 379.1718 | **S** | ***234.1448*** | 117.5761 | 217.1183 | 109.0628 | 216.1343 | 108.5708 | **2** |
| **9** |  |  |  |  | **K** | 147.1128 | 74.0600 | 130.0863 | 65.5468 |  |  | **1** |

MS/MS Fragmentation of **SSDILSVLK**
Found in **IQGA2_HUMAN**, Ras GTPase-activating-like protein IQGAP2 OS=Homo sapiens GN=IQGAP2 PE=1 SV=4


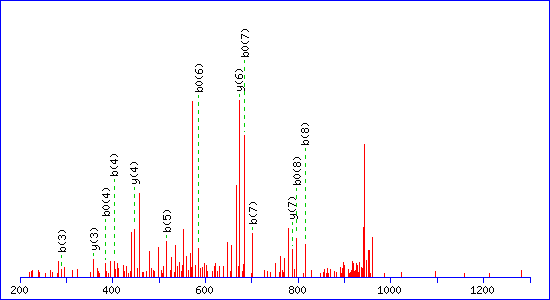
Match to Query 694: 960.622724 from(961.630000,1+) intensity(132804.0000) index(226)

**Monoisotopic mass of neutral peptide Mr(calc):** 960.5491

**Matches :** 13/37 fragment ions using 32 most intense peaks

| **#** | **b** | **b^0^** | **Seq.** | **y** | **y*** | **y^0^** | **#** |
| --- | --- | --- | --- | --- | --- | --- | --- |
| **1** | 88.0393 | 70.0287 | **S** |  |  |  | **9** |
| **2** | 175.0713 | 157.0608 | **S** | 874.5244 | 857.4979 | 856.5138 | **8** |
| **3** | ***290.0983*** | 272.0877 | **D** | ***787.4924*** | 770.4658 | 769.4818 | **7** |
| **4** | ***403.1823*** | 385.1718 | **I** | ***672.4654*** | 655.4389 | 654.4549 | **6** |
| **5** | ***516.2664*** | 498.2558 | **L** | 559.3814 | 542.3548 | 541.3708 | **5** |
| **6** | 603.2984 | 585.2879 | **S** | ***446.2973*** | 429.2708 | 428.2867 | **4** |
| **7** | ***702.3668*** | 684.3563 | **V** | ***359.2653*** | 342.2387 |  | **3** |
| **8** | ***815.4509*** | 797.4403 | **L** | 260.1969 | 243.1703 |  | **2** |
| **9** |  |  | **K** | 147.1128 | 130.0863 |  | **1** |

MS/MS Fragmentation of **LREEVVTK**
Found in **IQGA2_HUMAN**, Ras GTPase-activating-like protein IQGAP2 OS=Homo sapiens GN=IQGAP2 PE=1 SV=4


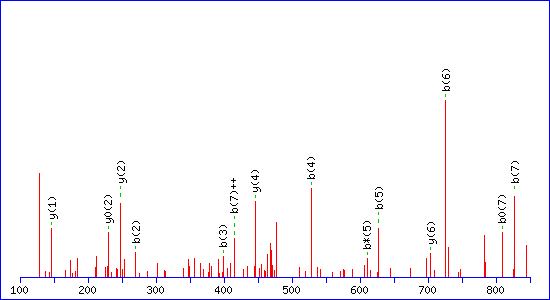
Match to Query 698: 972.765448 from(487.390000,2+) intensity(53742.0000) index(91)

**Monoisotopic mass of neutral peptide Mr(calc):** 972.5604

**Matches :** 14/76 fragment ions using 22 most intense peaks

| **#** | **b** | **b^++^** | **b*** | **b*^++^** | **b^0^** | **b^0++^** | **Seq.** | **y** | **y^++^** | **y*** | **y*^++^** | **y^0^** | **y^0++^** | **#** |
| --- | --- | --- | --- | --- | --- | --- | --- | --- | --- | --- | --- | --- | --- | --- |
| **1** | 114.0913 | 57.5493 |  |  |  |  | **L** |  |  |  |  |  |  | **8** |
| **2** | ***270.1925*** | 135.5999 | 253.1659 | 127.0866 |  |  | **R** | 860.4836 | 430.7454 | 843.4571 | 422.2322 | 842.4730 | 421.7402 | **7** |
| **3** | ***399.2350*** | 200.1212 | 382.2085 | 191.6079 | 381.2245 | 191.1159 | **E** | **704.3825** | 352.6949 | 687.3559 | 344.1816 | 686.3719 | 343.6896 | **6** |
| **4** | ***528.2776*** | 264.6425 | 511.2511 | 256.1292 | 510.2671 | 255.6372 | **E** | 575.3399 | 288.1736 | 558.3134 | 279.6603 | 557.3293 | 279.1683 | **5** |
| **5** | ***627.3461*** | 314.1767 | 610.3195 | 305.6634 | 609.3355 | 305.1714 | **V** | **446.2973** | 223.6523 | 429.2708 | 215.1390 | 428.2867 | 214.6470 | **4** |
| **6** | ***726.4145*** | 363.7109 | 709.3879 | 355.1976 | 708.4039 | 354.7056 | **V** | 347.2289 | 174.1181 | 330.2023 | 165.6048 | 329.2183 | 165.1128 | **3** |
| **7** | ***827.4621*** | 414.2347 | 810.4356 | 405.7214 | 809.4516 | 405.2294 | **T** | **248.1605** | 124.5839 | 231.1339 | 116.0706 | 230.1499 | 115.5786 | **2** |
| **8** |  |  |  |  |  |  | **K** | **147.1128** | 74.0600 | 130.0863 | 65.5468 |  |  | **1** |

MS/MS Fragmentation of **IYDVEQTR**
Found in **IQGA2_HUMAN**, Ras GTPase-activating-like protein IQGAP2 OS=Homo sapiens GN=IQGAP2 PE=1 SV=4


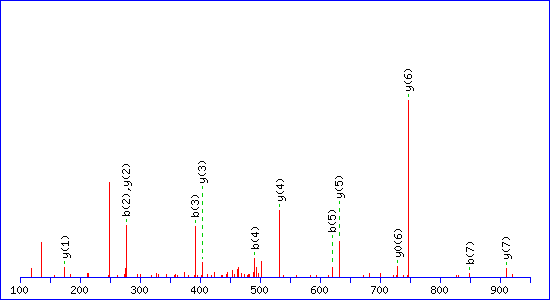
Match to Query 710: 1022.745448 from(512.380000,2+) intensity(97055.0000) index(131)

**Monoisotopic mass of neutral peptide Mr(calc):** 1022.5033

**Matches :** 13/68 fragment ions using 17 most intense peaks

| **#** | **b** | **b^++^** | **b*** | **b*^++^** | **b^0^** | **b^0++^** | **Seq.** | **y** | **y^++^** | **y*** | **y*^++^** | **y^0^** | **y^0++^** | **#** |
| --- | --- | --- | --- | --- | --- | --- | --- | --- | --- | --- | --- | --- | --- | --- |
| **1** | 114.0913 | 57.5493 |  |  |  |  | **I** |  |  |  |  |  |  | **8** |
| **2** | **277.1547** | 139.0810 |  |  |  |  | **Y** | ***910.4265*** | 455.7169 | 893.3999 | 447.2036 | 892.4159 | 446.7116 | **7** |
| **3** | **392.1816** | 196.5944 |  |  | 374.1710 | 187.5892 | **D** | ***747.3632*** | 374.1852 | 730.3366 | 365.6719 | 729.3526 | 365.1799 | **6** |
| **4** | **491.2500** | 246.1287 |  |  | 473.2395 | 237.1234 | **V** | ***632.3362*** | 316.6717 | 615.3097 | 308.1585 | 614.3257 | 307.6665 | **5** |
| **5** | **620.2926** | 310.6499 |  |  | 602.2821 | 301.6447 | **E** | ***533.2678*** | 267.1375 | 516.2413 | 258.6243 | 515.2572 | 258.1323 | **4** |
| **6** | 748.3512 | 374.6792 | 731.3246 | 366.1660 | 730.3406 | 365.6740 | **Q** | ***404.2252*** | 202.6162 | 387.1987 | 194.1030 | 386.2146 | 193.6110 | **3** |
| **7** | **849.3989** | 425.2031 | 832.3723 | 416.6898 | 831.3883 | 416.1978 | **T** | ***276.1666*** | 138.5870 | 259.1401 | 130.0737 | 258.1561 | 129.5817 | **2** |
| **8** |  |  |  |  |  |  | **R** | ***175.1190*** | 88.0631 | 158.0924 | 79.5498 |  |  | **1** |

MS/MS Fragmentation of **LIFQMPQNK**
Found in **IQGA2_HUMAN**, Ras GTPase-activating-like protein IQGAP2 OS=Homo sapiens GN=IQGAP2 PE=1 SV=4


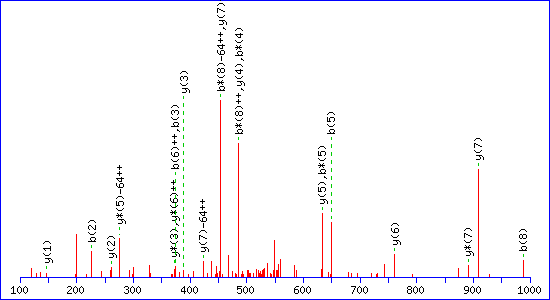
Match to Query 738: 1133.805448 from(567.910000,2+) intensity(136202.0000) index(166)

**Monoisotopic mass of neutral peptide Mr(calc):** 1133.5903

**Variable modifications:**

**M5 :** Oxidation (M), with neutral losses 0.0000(shown in table), 63.9983

**Matches :** 22/90 fragment ions using 33 most intense peaks

| **#** | **b** | **b^++^** | **b*** | **b*^++^** | **Seq.** | **y** | **y^++^** | **y*** | **y*^++^** | **#** |
| --- | --- | --- | --- | --- | --- | --- | --- | --- | --- | --- |
| **1** | 114.0913 | 57.5493 |  |  | **L** |  |  |  |  | **9** |
| **2** | **227.1754** | 114.0913 |  |  | **I** | 1021.5135 | 511.2604 | 1004.4870 | 502.7471 | **8** |
| **3** | **374.2438** | 187.6255 |  |  | **F** | ***908.4295*** | 454.7184 | 891.4029 | 446.2051 | **7** |
| **4** | 502.3024 | 251.6548 | 485.2758 | 243.1416 | **Q** | ***761.3611*** | 381.1842 | 744.3345 | 372.6709 | **6** |
| **5** | **649.3378** | 325.1725 | 632.3112 | 316.6593 | **M** | ***633.3025*** | 317.1549 | 616.2759 | 308.6416 | **5** |
| **6** | 746.3906 | 373.6989 | 729.3640 | 365.1856 | **P** | ***486.2671*** | 243.6372 | 469.2405 | 235.1239 | **4** |
| **7** | 874.4491 | 437.7282 | 857.4226 | 429.2149 | **Q** | ***389.2143*** | 195.1108 | 372.1878 | 186.5975 | **3** |
| **8** | **988.4921** | 494.7497 | 971.4655 | 486.2364 | **N** | ***261.1557*** | 131.0815 | 244.1292 | 122.5682 | **2** |
| **9** |  |  |  |  | **K** | ***147.1128*** | 74.0600 | 130.0863 | 65.5468 | **1** |

MS/MS Fragmentation of **KIYDVEQTR**
Found in **IQGA2_HUMAN**, Ras GTPase-activating-like protein IQGAP2 OS=Homo sapiens GN=IQGAP2 PE=1 SV=4


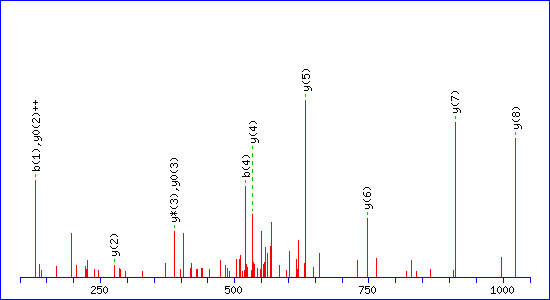
Match to Query 740: 1150.765448 from(576.390000,2+) intensity(101101.0000) index(117)

**Monoisotopic mass of neutral peptide Mr(calc):** 1150.5982

**Matches :** 11/88 fragment ions using 9 most intense peaks

| **#** | **b** | **b^++^** | **b*** | **b*^++^** | **b^0^** | **b^0++^** | **Seq.** | **y** | **y^++^** | **y*** | **y*^++^** | **y^0^** | **y^0++^** | **#** |
| --- | --- | --- | --- | --- | --- | --- | --- | --- | --- | --- | --- | --- | --- | --- |
| **1** | **129.1022** | 65.0548 | 112.0757 | 56.5415 |  |  | **K** |  |  |  |  |  |  | **9** |
| **2** | 242.1863 | 121.5968 | 225.1598 | 113.0835 |  |  | **I** | ***1023.5106*** | 512.2589 | 1006.4840 | 503.7456 | 1005.5000 | 503.2536 | **8** |
| **3** | 405.2496 | 203.1285 | 388.2231 | 194.6152 |  |  | **Y** | ***910.4265*** | 455.7169 | 893.3999 | 447.2036 | 892.4159 | 446.7116 | **7** |
| **4** | **520.2766** | 260.6419 | 503.2500 | 252.1287 | 502.2660 | 251.6366 | **D** | ***747.3632*** | 374.1852 | 730.3366 | 365.6719 | 729.3526 | 365.1799 | **6** |
| **5** | 619.3450 | 310.1761 | 602.3184 | 301.6629 | 601.3344 | 301.1709 | **V** | ***632.3362*** | 316.6717 | 615.3097 | 308.1585 | 614.3257 | 307.6665 | **5** |
| **6** | 748.3876 | 374.6974 | 731.3610 | 366.1842 | 730.3770 | 365.6921 | **E** | ***533.2678*** | 267.1375 | 516.2413 | 258.6243 | 515.2572 | 258.1323 | **4** |
| **7** | 876.4462 | 438.7267 | 859.4196 | 430.2134 | 858.4356 | 429.7214 | **Q** | 404.2252 | 202.6162 | 387.1987 | 194.1030 | 386.2146 | 193.6110 | **3** |
| **8** | 977.4938 | 489.2506 | 960.4673 | 480.7373 | 959.4833 | 480.2453 | **T** | ***276.1666*** | 138.5870 | 259.1401 | 130.0737 | 258.1561 | 129.5817 | **2** |
| **9** |  |  |  |  |  |  | **R** | 175.1190 | 88.0631 | 158.0924 | 79.5498 |  |  | **1** |

MS/MS Fragmentation of **VDQVQDIVTGNPTVIK**
Found in **IQGA2_HUMAN**, Ras GTPase-activating-like protein IQGAP2 OS=Homo sapiens GN=IQGAP2 PE=1 SV=4


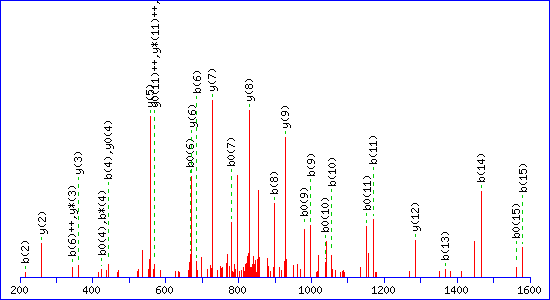
Match to Query 804: 1725.325448 from(863.670000,2+) intensity(238932.0000) index(199)

**Monoisotopic mass of neutral peptide Mr(calc):** 1724.9309

**Matches :** 33/168 fragment ions using 47 most intense peaks

| **#** | **b** | **b^++^** | **b*** | **b*^++^** | **b^0^** | **b^0++^** | **Seq.** | **y** | **y^++^** | **y*** | **y*^++^** | **y^0^** | **y^0++^** | **#** |
| --- | --- | --- | --- | --- | --- | --- | --- | --- | --- | --- | --- | --- | --- | --- |
| **1** | 100.0757 | 50.5415 |  |  |  |  | **V** |  |  |  |  |  |  | **16** |
| **2** | ***215.1026*** | 108.0550 |  |  | 197.0921 | 99.0497 | **D** | 1626.8697 | 813.9385 | 1609.8432 | 805.4252 | 1608.8592 | 804.9332 | **15** |
| **3** | ***343.1612*** | 172.0842 | 326.1347 | 163.5710 | 325.1506 | 163.0790 | **Q** | 1511.8428 | 756.4250 | 1494.8162 | 747.9118 | 1493.8322 | 747.4197 | **14** |
| **4** | ***442.2296*** | 221.6185 | 425.2031 | 213.1052 | 424.2191 | 212.6132 | **V** | 1383.7842 | 692.3957 | 1366.7577 | 683.8825 | 1365.7736 | 683.3905 | **13** |
| **5** | ***570.2882*** | 285.6477 | 553.2617 | 277.1345 | 552.2776 | 276.6425 | **Q** | **1284.7158** | 642.8615 | 1267.6892 | 634.3483 | 1266.7052 | 633.8563 | **12** |
| **6** | ***685.3151*** | 343.1612 | 668.2886 | 334.6479 | 667.3046 | 334.1559 | **D** | 1156.6572 | 578.8322 | 1139.6307 | 570.3190 | 1138.6467 | 569.8270 | **11** |
| **7** | 798.3992 | 399.7032 | 781.3727 | 391.1900 | 780.3886 | 390.6980 | **I** | 1041.6303 | 521.3188 | 1024.6037 | 512.8055 | 1023.6197 | 512.3135 | **10** |
| **8** | ***897.4676*** | 449.2375 | 880.4411 | 440.7242 | 879.4571 | 440.2322 | **V** | **928.5462** | 464.7767 | 911.5197 | 456.2635 | 910.5356 | 455.7715 | **9** |
| **9** | ***998.5153*** | 499.7613 | 981.4888 | 491.2480 | 980.5047 | 490.7560 | **T** | **829.4778** | 415.2425 | 812.4512 | 406.7293 | 811.4672 | 406.2373 | **8** |
| **10** | ***1055.5368*** | 528.2720 | 1038.5102 | 519.7587 | 1037.5262 | 519.2667 | **G** | **728.4301** | 364.7187 | 711.4036 | 356.2054 | 710.4196 | 355.7134 | **7** |
| **11** | ***1169.5797*** | 585.2935 | 1152.5531 | 576.7802 | 1151.5691 | 576.2882 | **N** | **671.4087** | 336.2080 | 654.3821 | 327.6947 | 653.3981 | 327.2027 | **6** |
| **12** | 1266.6325 | 633.8199 | 1249.6059 | 625.3066 | 1248.6219 | 624.8146 | **P** | **557.3657** | 279.1865 | 540.3392 | 270.6732 | 539.3552 | 270.1812 | **5** |
| **13** | ***1367.6801*** | 684.3437 | 1350.6536 | 675.8304 | 1349.6696 | 675.3384 | **T** | 460.3130 | 230.6601 | 443.2864 | 222.1468 | 442.3024 | 221.6548 | **4** |
| **14** | ***1466.7486*** | 733.8779 | 1449.7220 | 725.3646 | 1448.7380 | 724.8726 | **V** | **359.2653** | 180.1363 | 342.2387 | 171.6230 |  |  | **3** |
| **15** | ***1579.8326*** | 790.4199 | 1562.8061 | 781.9067 | 1561.8220 | 781.4147 | **I** | **260.1969** | 130.6021 | 243.1703 | 122.0888 |  |  | **2** |
| **16** |  |  |  |  |  |  | **K** | 147.1128 | 74.0600 | 130.0863 | 65.5468 |  |  | **1** |

MS/MS Fragmentation of **GGEMEILNNTDNQGIK**
Found in **IQGA2_HUMAN**, Ras GTPase-activating-like protein IQGAP2 OS=Homo sapiens GN=IQGAP2 PE=1 SV=4


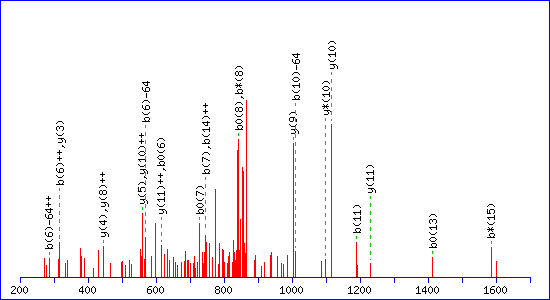
Match to Query 807: 1748.025448 from(875.020000,2+) intensity(263304.0000) index(153)

**Monoisotopic mass of neutral peptide Mr(calc):** 1747.8047

**Variable modifications:**

**M4 :** Oxidation (M), with neutral losses 0.0000(shown in table), 63.9983

**Matches :** 23/234 fragment ions using 23 most intense peaks

| **#** | **b** | **b^++^** | **b*** | **b*^++^** | **b^0^** | **b^0++^** | **Seq.** | **y** | **y^++^** | **y*** | **y*^++^** | **y^0^** | **y^0++^** | **#** |
| --- | --- | --- | --- | --- | --- | --- | --- | --- | --- | --- | --- | --- | --- | --- |
| **1** | 58.0287 | 29.5180 |  |  |  |  | **G** |  |  |  |  |  |  | **16** |
| **2** | 115.0502 | 58.0287 |  |  |  |  | **G** | 1691.7905 | 846.3989 | 1674.7639 | 837.8856 | 1673.7799 | 837.3936 | **15** |
| **3** | 244.0928 | 122.5500 |  |  | 226.0822 | 113.5448 | **E** | 1634.7690 | 817.8882 | 1617.7425 | 809.3749 | 1616.7585 | 808.8829 | **14** |
| **4** | 391.1282 | 196.0677 |  |  | 373.1176 | 187.0625 | **M** | 1505.7264 | 753.3669 | 1488.6999 | 744.8536 | 1487.7159 | 744.3616 | **13** |
| **5** | 520.1708 | 260.5890 |  |  | 502.1602 | 251.5838 | **E** | 1358.6910 | 679.8492 | 1341.6645 | 671.3359 | 1340.6805 | 670.8439 | **12** |
| **6** | 633.2549 | **317.1311** |  |  | 615.2443 | 308.1258 | **I** | ***1229.6484*** | 615.3279 | 1212.6219 | 606.8146 | 1211.6379 | 606.3226 | **11** |
| **7** | **746.3389** | 373.6731 |  |  | 728.3284 | 364.6678 | **L** | ***1116.5644*** | 558.7858 | 1099.5378 | 550.2726 | 1098.5538 | 549.7805 | **10** |
| **8** | 860.3818 | 430.6946 | 843.3553 | 422.1813 | 842.3713 | 421.6893 | **N** | ***1003.4803*** | 502.2438 | 986.4538 | 493.7305 | 985.4697 | 493.2385 | **9** |
| **9** | 974.4248 | 487.7160 | 957.3982 | 479.2027 | 956.4142 | 478.7107 | **N** | 889.4374 | 445.2223 | 872.4108 | 436.7091 | 871.4268 | 436.2170 | **8** |
| **10** | 1075.4725 | 538.2399 | 1058.4459 | 529.7266 | 1057.4619 | 529.2346 | **T** | 775.3945 | 388.2009 | 758.3679 | 379.6876 | 757.3839 | 379.1956 | **7** |
| **11** | **1190.4994** | 595.7533 | 1173.4728 | 587.2401 | 1172.4888 | 586.7481 | **D** | 674.3468 | 337.6770 | 657.3202 | 329.1638 | 656.3362 | 328.6717 | **6** |
| **12** | 1304.5423 | 652.7748 | 1287.5158 | 644.2615 | 1286.5318 | 643.7695 | **N** | ***559.3198*** | 280.1636 | 542.2933 | 271.6503 |  |  | **5** |
| **13** | 1432.6009 | 716.8041 | 1415.5744 | 708.2908 | 1414.5903 | 707.7988 | **Q** | ***445.2769*** | 223.1421 | 428.2504 | 214.6288 |  |  | **4** |
| **14** | 1489.6224 | **745.3148** | 1472.5958 | 736.8015 | 1471.6118 | 736.3095 | **G** | ***317.2183*** | 159.1128 | 300.1918 | 150.5995 |  |  | **3** |
| **15** | 1602.7064 | 801.8569 | 1585.6799 | 793.3436 | 1584.6959 | 792.8516 | **I** | 260.1969 | 130.6021 | 243.1703 | 122.0888 |  |  | **2** |
| **16** |  |  |  |  |  |  | **K** | 147.1128 | 74.0600 | 130.0863 | 65.5468 |  |  | **1** |
