# Supplementary figures and images for: The Pseudomonas aeruginosa N-Acylhomoserine Lactone Quorum Sensing Molecules Target IQGAP1 and Modulate Epithelial Cell Migration
Source: PLoS Pathog. 2012 Oct 11;8(10):e1002953. doi: 10.1371/journal.ppat.1002953 (PMC3469656; doi:10.1371/journal.ppat.1002953)

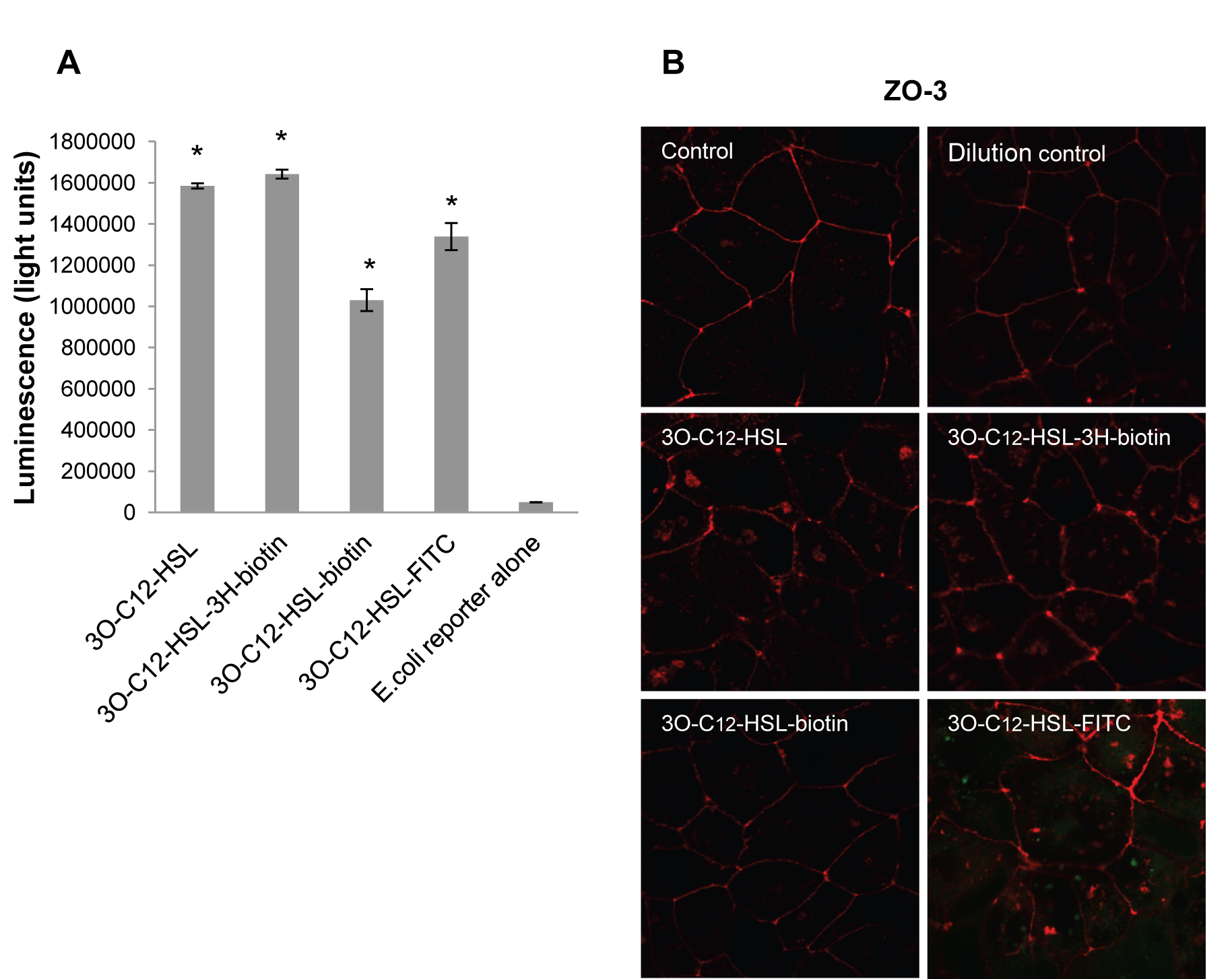

Supplement: Figure S1 — Biological activity of synthetic AHL molecules used in this study. (A) Induction of luminescence in lux-based AHL biosensor reporter bacteria (E.coli JM109 pSB1075) by 10 ng of N-3-oxo-dodecanoyl-L-homoserine lactone C16H27NO4, (3O-C12-HSL), biotin-conjugated probe N-dodecanoyl-L-homoserine lactone-3-hydrazone-biotin C26H43N5O5S, (3O-C12-HSL-3H-biotin), N-dodecanoyl-L-homoserine lactone-biotin C38H41N3O9S, (3O-C12-HSL-biotin) and fluorescently-tagged probe N-dodecanoyl-L-homoserine lactone-3-hydrazone-fluorescein, C37H40N4O8S, (3O-C12-HSL-FITC). As additional controls for the bioassay, bacteria reporter in LB medium (not shown) or in LB medium containing diluents was used (E.coli reporter alone). Luminescence was measured after 4-h growth. Displayed are the mean ± standard errors of at least six independent experiments performed on separate days. Significant differences (*) in mean for luminescence compared with values for luminescence of E.coli reporter alone as a control as calculated by Student's t test. (B) Effect of synthetic AHL molecules on ZO-3 junction protein distribution in human epithelial Caco-2 cells. Caco-2 cell monolayers were treated with 1 µM 3O-C12-HSL, 3O-C12-HSL-3H-biotin, 3O-C12-HSL-biotin, 3O-C12-HSL-FITC (green) or diluents as a control, for 5 h. Cells were fixed and stained with antibodies against ZO-3 and Alexa Fluor 594 secondary antibodies (red) and analyzed by confocal laser scanning microscopy. The images are from one representative of at least three independent experiments. Image size is 67.6×67.6 µm and pixel size is 0.13 µm. (TIF) [file ppat.1002953.s003.tif]

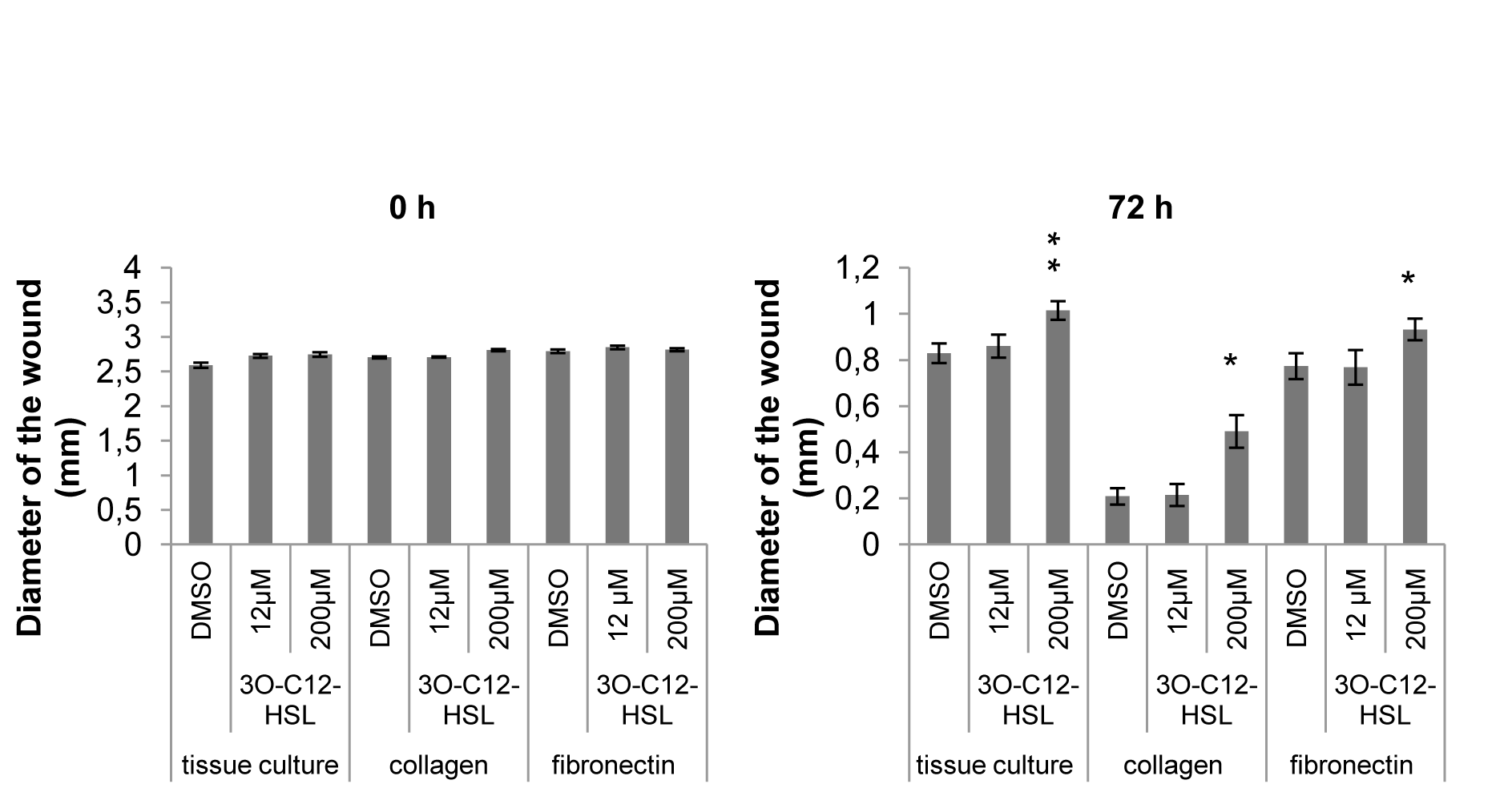

Supplement: Figure S2 — Effect of 3O-C12-HSL on migration of epithelial Caco-2 cells. Caco-2 cells were cultured to form monolayers on tissue culture-, rat tail collagen-, or human fibronectin-coated 96-well plates with a cylinder-like plug in each well. The circular, 2 mm diameter wound was created by removing the plug. Cells were incubated with 12 and 200 µM 3O-C12-HSL. Control cells were either untreated (data not shown) or treated with 0.018% DMSO as a diluent control. For each well, one image was taken at 0, 24, 48 and 72 h. The migration rate was calculated by measuring the diameter of the wounds (three measurements per image for each well and each time point) using Image J software. Shown is the mean ± standard errors of at least three independent experiments in eight identical wells performed on separate days from different cell passages. Significant differences (* - P≤0.05; ** - P≤0.01) in mean for migration rate compared with values for cells in the control group as calculated by Student's t test. (TIF) [file ppat.1002953.s004.tif]

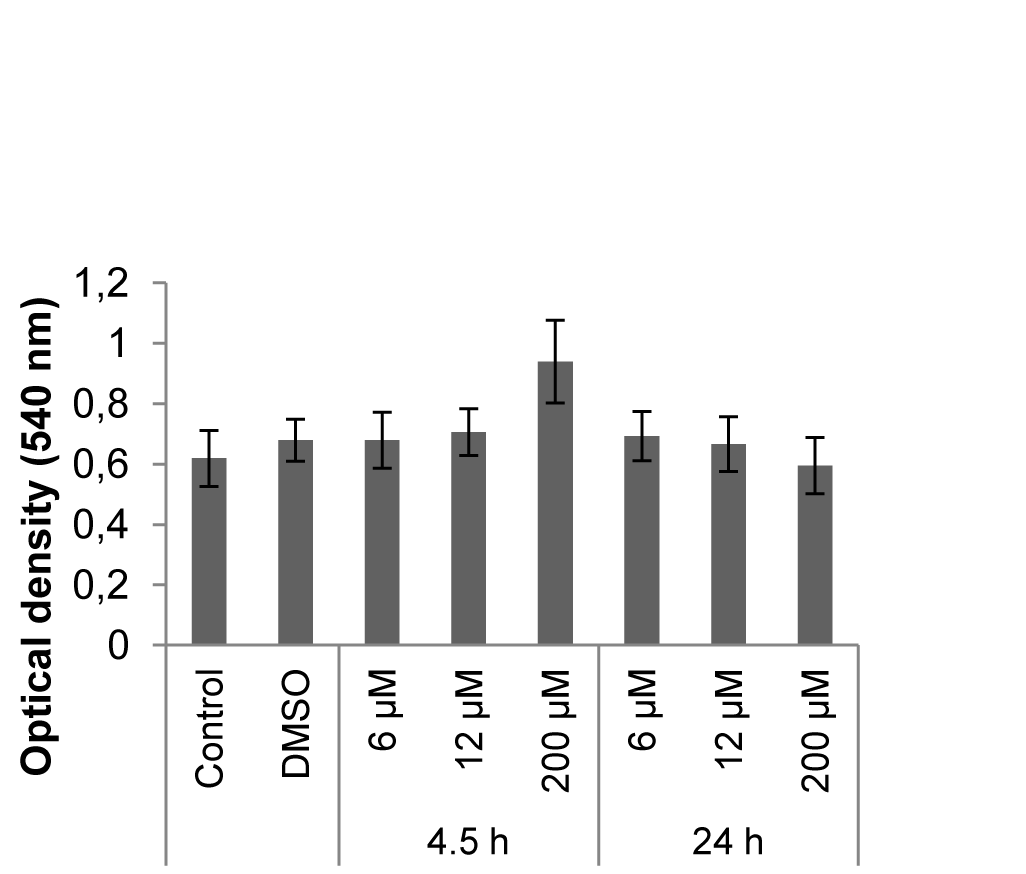

Supplement: Figure S3 — Proliferation of epithelial Caco-2 cells treated with 3O-C12-HSL. Cell monolayers cultured in 96-well plates were treated with 6, 12 and 200 µM 3O-C12-HSL for 4.5 or 24 h. Control cells were untreated or treated with 0.018% DMSO. This figure shows the mean ± standard error based on at least six independent experiments in eight identical wells performed on different days. (TIF) [file ppat.1002953.s005.tif]

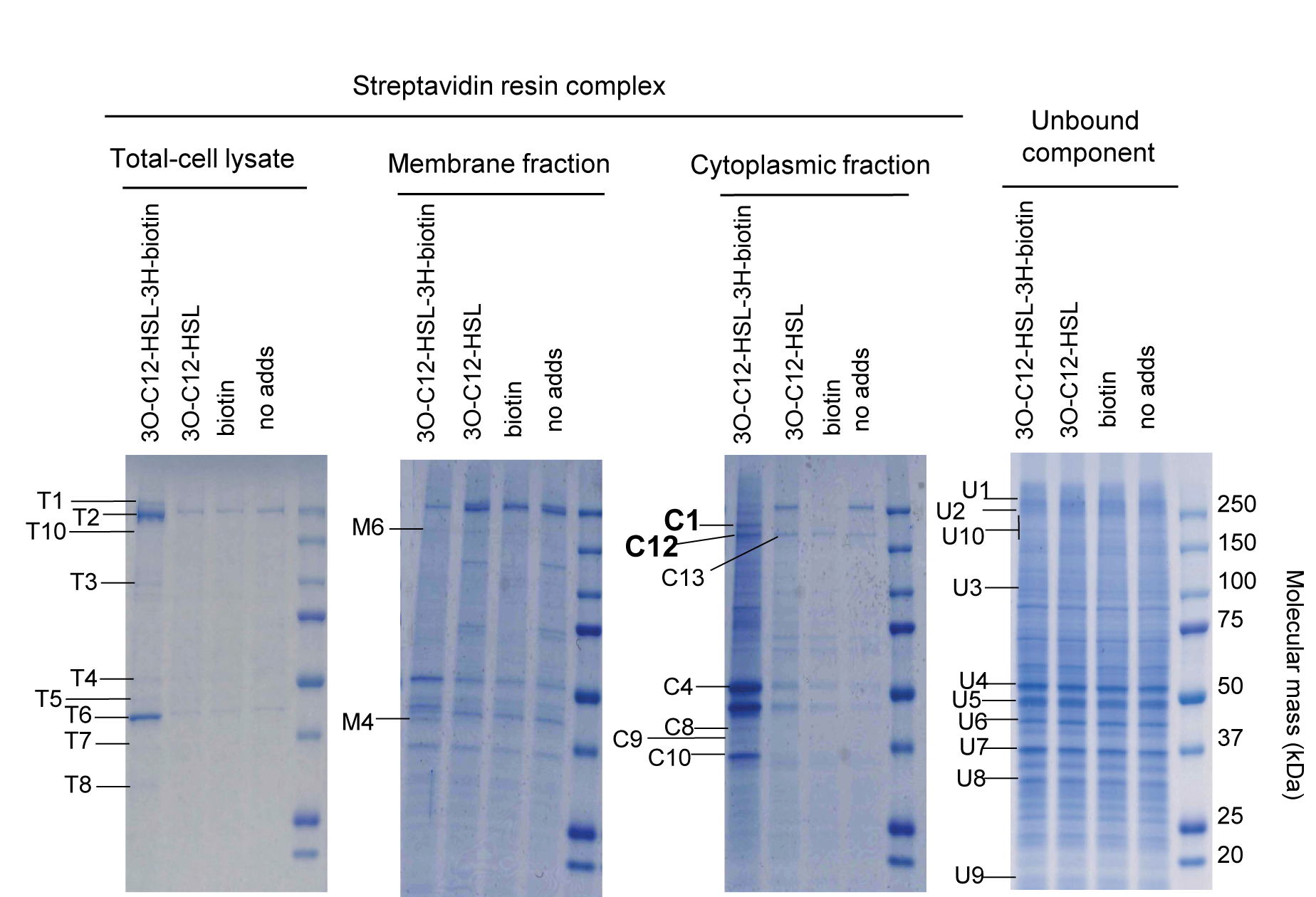

Supplement: Figure S4 — SDS-PAGE of 3O-C12-HSL-3H-biotin complexes from Caco-2 cells. Total-cell lysate, cytoplasmic or membrane fraction was incubated with 0.05 mg 3O-C12-HSL-3H-biotin, 0.05 mg 3O-C12-HSL, and 4 µg biotin or without any additions (as controls). Streptavidin agarose resin-captured complexes were analyzed by SDS-PAGE and subsequently stained with PageBlue protein staining solution. Shown are representative gels from one of three independent experiments performed on separate days from different reactions, fraction isolation and cell passages. Bands C1 and C12 represent proteins IQGAP1 and 2 respectively identified by in-gel digestion and LC-MS/MS analysis as shown in Table 1. Background protein contaminants are shown in Table S1. Peptide identification views from MASCOT MS data analyses of IQGAP1 and 2 are shown in supporting information (Dataset S1 and S2). (TIF) [file ppat.1002953.s006.tif]

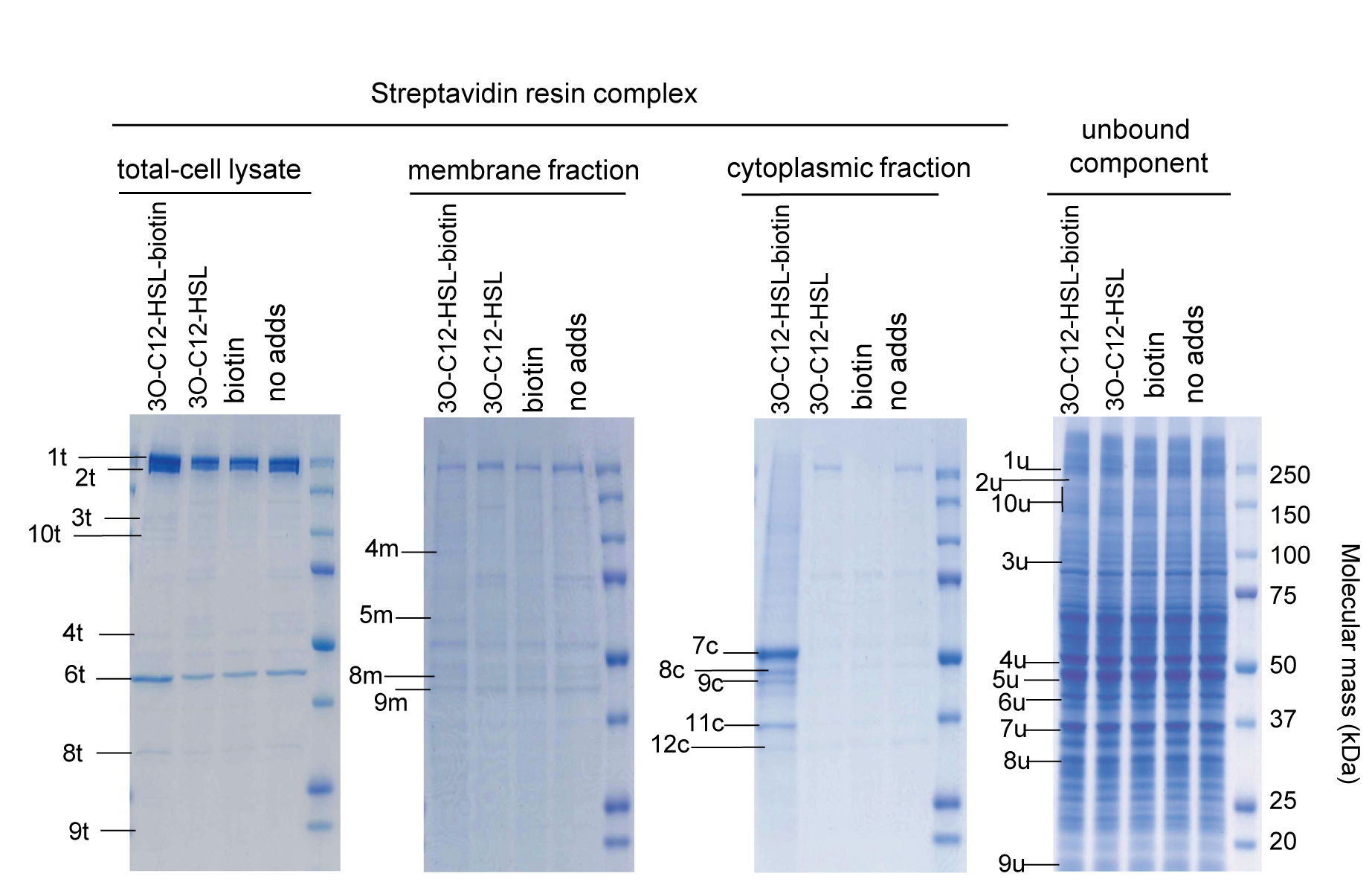

Supplement: Figure S5 — SDS-PAGE of 3O-C12-HSL-biotin complexes from Caco-2 cells. Total-cell lysate, cytoplasmic or membrane fraction was incubated with 0.05 mg 3O-C12-HSL-biotin, 0.05 mg 3O-C12-HSL, and 4 µg biotin or without any additions (as controls). Streptavidin agarose resin-captured complexes were analyzed by SDS-PAGE and subsequently stained with PageBlue protein staining solution. Displayed are representative gels from one of three independent experiments performed on separate days from different reactions, fraction isolation and cell passages. Indicated bands represent proteins identified by in-gel digestion and LC-MS/MS analysis as shown in Table S2. (TIF) [file ppat.1002953.s007.tif]
