# Supplementary material for: The Pseudomonas aeruginosa N-Acylhomoserine Lactone Quorum Sensing Molecules Target IQGAP1 and Modulate Epithelial Cell Migration
Source: PLoS Pathog. 2012 Oct 11;8(10):e1002953. doi: 10.1371/journal.ppat.1002953 (PMC3469656; doi:10.1371/journal.ppat.1002953)
Supplement: Protocol S1 — (DOCX) [file ppat.1002953.s008.docx]

Proliferation and viability assay

The proliferation and viability of Caco-2 cells treated with 3O-C_12_-HSL were determined with a mitochondrial toxicity test (MTT), based on measuring mitochondrial respiration, as assessed by the reduction of 3-(4,5 dimethylthiazol-2yl)-2,5 diphenyl-tetrazolium bromide (Sigma, St. Louis, MO) to formazan. Briefly, cells were cultured in 96-well plates until reaching 70-80 % confluent monolayers, serum-starved overnight and treated with 0.018 % DMSO, or 6, 12 and 200 µM 3O-C_12_-HSL for 4.5 or 24 h. After this, 50 µL of 5 mg/ml MTT in PBS was added to each well for 2 h, and then the medium was removed. Remaining blue formazan crystals were dissolved in 100 µL 4 mM HCl, 0.1 % NP-40 in isopropanol for 15 min, and the optical density at 540 nm was detected in a plate reader (Molecular Devices Inc, Sunnyvale, CA). Independent experiments were performed six times on separate days, each in eight identical wells.

Caco-2 cell proliferation after 3O-C_12_-HSL treatment

The inhibitory effect of 100 and 200 µM 3O-C_12_-HSL on Caco-2 cells migration raised a question of whether it might suppress cell proliferation activity or even be cytotoxic. We thus assessed proliferation and viability of Caco-2 cells by a mitochondrial toxicity test (MTT), based on measuring mitochondrial respiration, as assessed by the reduction of 3-(4,5 dimethylthiazol-2yl)-2,5 diphenyl-tetrazolium bromide (Sigma, St. Louis, MO) to formazan. We found no differences in cell proliferation between cells being untreated, or treated with 0.018 % DMSO, or 6, 12, and 200 µM 3O-C_12_-HSL (Figure S3).
