# Supplementary material for: The Pseudomonas aeruginosa N-Acylhomoserine Lactone Quorum Sensing Molecules Target IQGAP1 and Modulate Epithelial Cell Migration
Source: PLoS Pathog. 2012 Oct 11;8(10):e1002953. doi: 10.1371/journal.ppat.1002953 (PMC3469656; doi:10.1371/journal.ppat.1002953)
Supplement: Table S1 — Proteins identified in 3O-C12-HSL-3H-biotin affinity complexes from Caco-2 cells using in-gel digestion and LC-MS/MS analysis. (DOCX) [file ppat.1002953.s009.docx]

**Table S1. Proteins identified in 3O-C_12_-HSL-3H-biotin affinity complexes from Caco-2 cells using in-gel digestion and LC-MS/MS analysis.**

| Band | Protein | Theoretical MW, kDa | Number of unique peptides |
| --- | --- | --- | --- |
|  | *Positive data* |  |  |
| **C1** | **IQGAP1** | **189.7** | **24** |
| **C12** | **IQGAP2** | **181.0** | **8** |
|  | *Background contaminants* |  |  |
| T1 | Spectrin alpha | 285.1 | 10 |
|  | Myosin-9 | 227.6 | 5 |
|  | Spectrin beta | 275.2 | 10 |
| T10 | Myosin-10 | 227.6 | 3 |
| T2 | Myosin-9 | 226.3 | 41 |
|  | Myosin-10 | 228.8 | 7 |
|  | Myosin-11 | 227.1 | 5 |
| T3 | Alpha-actinin-4 | 104.7 | 8 |
|  | Villin-1 | 92.6 | 8 |
| T4 | Tubulin alpha-1B chain | 50.8 | 7 |
|  | Tubulin beta chain | 50.0 | 5 |
|  | Keratin, type 2 | 53.6 | 8 |
| T5 | Keratin, type I cytoskeletal | 62.2 | 17 |
|  | Elongation factor 1-alpha | 50.4 | 8 |
|  | Keratin, type II cytoskeletal | 60.3 | 4 |
|  | Serpin | 46.5 | 2 |
| T6 | Actin cytoplasmic 1 | 42.0 | 13 |
|  | Actin alpha | 42.3 | 12 |
|  | Beta-actin-like protein 2 | 42.3 | 5 |
| T7 | GAPDH | 36.2 | 6 |
|  | Keratin, type II cytoskeletal | 66.1 | 7 |
|  | Keratin, type I cytoskeletal | 62.2 | 2 |
| T8 | Tropomyosin alpha-4 chain | 28.6 | 4 |
|  | Tropomyosin alpha-3 chain | 32.8 | 4 |
| M6 | Clathrin heavy chain | 193.2 | 4 |
| M4 | Keratin, type I | 44.0 | 16 |
| C4 | Keratin, type II | 53.6 | 13 |
|  | Tubulin alpha-1A | 50.7 | 9 |
| C8 | Keratin, type I | 44.0 | 14 |
| C9 | DnaJ homolog subfamily B member 1 | 38.1 | 8 |
| C10 | GAPDH | 36.2 | 17 |
| C12 | Bifunctional aminoacyl-tRNA synthetase | 172 | 10 |
| C13 | Bifunctional aminoacyl-tRNA synthetase | 172 | 6 |
| U1 | Filamin-B | 280.1 | 5 |
|  | Filamin-A | 283.3 | 2 |
| U2 | Transmembrane coiled-coil domains protein | 77.6 | 1 |
| U10 | Splicing factor 3B subunit 1 | 146.4 | 1 |
| U3 | Elongation factor 2 | 92.6 | 5 |
| U4 | Tubulin alpha-1B chain | 50.8 | 10 |
|  | Tubulin beta chain | 50.0 | 5 |
|  | Tubulin beta2C chain | 50.2 | 5 |
| U5 | Alpha-enolase | 47.4 | 11 |
|  | Beta-enolase | 47.2 | 4 |
|  | Gamma-enolase | 47.5 | 3 |
|  | Elongation factor 1-alpha | 50.4 | 9 |
|  | Serpin | 46.5 | 3 |
| U6 | Actin cytoplasmic 1 | 42.0 | 9 |
|  | Beta-actin-like protein 2 | 42.3 | 2 |
| U7 | GAPDH | 36.2 | 7 |
| U8 | Annexin A5 | 35.9 | 6 |
| U9 | Pepdyl-prolyl cis-trans isomerase A | 18.2 | 5 |

T – bands of 3O-C_12_-HSL-3H-biotin complexes from total-cell lysate; M - bands of 3O-C_12_-HSL-3H-biotin complexes from membrane fraction; C - bands of 3O-C_12_-HSL-3H-biotin complexes from cytoplasmic fraction; U - bands of unbound components; IQGAP –IQ motif containing GTPase activating protein; GAPDH – glyceraldehydes-3-phosphate dehydrogenase.
