# Supplementary material for: The Pseudomonas aeruginosa N-Acylhomoserine Lactone Quorum Sensing Molecules Target IQGAP1 and Modulate Epithelial Cell Migration
Source: PLoS Pathog. 2012 Oct 11;8(10):e1002953. doi: 10.1371/journal.ppat.1002953 (PMC3469656; doi:10.1371/journal.ppat.1002953)
Supplement: Table S2 — Background proteins from 3O-C12-HSL-biotin affinity procedure in Caco-2 cells. (DOCX) [file ppat.1002953.s010.docx]

**Table S2. Background proteins from 3O-C_12_-HSL-biotin affinity procedure in Caco-2 cells.**

| Band | Protein | MW, kDa | Number of unique peptides |
| --- | --- | --- | --- |
| 1t | Myosin-9 | 227.6 | 32 |
|  | Myosin-14 | 228.7 | 6 |
|  | Myosin-10 | 229.8 | 7 |
|  | Myosin-11 | 228.0 | 8 |
| 2t | Myosin-9 | 227.6 | 38 |
|  | Myosin-10 | 229.8 | 10 |
|  | Myosin-14 | 228.7 | 4 |
|  | Myosin-11 | 228.0 | 5 |
| 3t | Ribonucleoprotein U | 91.2 | 2 |
| 10t | Villin-1 | 93.0 | 5 |
|  | Alpha-actinin-4 | 105.2 | 3 |
| 4t | Tubulin alpha-1A chain | 50.7 | 2 |
| 6t | Actin cytoplasmic 1 | 42.0 | 8 |
|  | Actin alpha | 42.3 | 7 |
| 8t | Tropomyosin alpha-3 chain | 32.8 | 2 |
|  | Tropomyosin alpha-4 chain | 28.6 | 2 |
| 9t | Myosin light polypeptide 6 | 17.0 | 2 |
| 4m | Heat shock protein 90-beta | 85.0 | 4 |
| 5m | Heat shock protein 60 | 61.1 | 6 |
| 8m | Keratin, type I cytoskeletal | 48.0 | 5 |
| 9m | Actin cytoplasmic 1 | 42.0 | 2 |
| 7c | Tubulin alpha-1B chain | 50.8 | 10 |
|  | Tubulin beta chain | 50.0 | 7 |
|  | Keratin, type II cytoskeletal | 53.6 | 10 |
|  | Tubulin beta-8 chain | 50.2 | 4 |
| 8c | Elongation factor 1-alpha | 50.4 | 4 |
| 9c | Keratin, type I cytoskeletal | 48.0 | 18 |
| 11c | GAPDH | 36.2 | 6 |
| 1u | Filamin-B | 280.0 | 14 |
|  | Spectrin | 285.1 | 2 |
|  | Filamin A | 283.3 | 5 |
| 2u | Myosin-9 | 227.6 | 7 |
|  | Talin-1 | 271.7 | 2 |
| 10u | Translation initiation factor 3 subunit A | 166.8 | 2 |
| 3u | Exportin-2 | 111.1 | 6 |
|  | Elongation factor 2 | 96.2 | 11 |
| 4u | Tubulin alpha-1B chain | 50.8 | 13 |
|  | Tubulin alpha-1C chain | 50.5 | 13 |
|  | Tubulin beta chain | 50.0 | 8 |
| 5u | Elongation factor 1-alpha | 50.4 | 12 |
|  | Elongation factor 1-gamma | 50.4 | 3 |
| 6u | Actin cytoplasmic 1 | 42.0 | 9 |
| 7u | Annexin A5 | 38.8 | 9 |
|  | GAPDH | 36.2 | 5 |
| 8u | Annexin A5 | 35.9 | 4 |
| 9u | Pepdyl-prolyl cis-trans isomerase A | 18.2 | 3 |

t – bands of 3O-C_12_-HSL-biotin complexes from total-cell lysate; m - bands of 3O-C_12_-HSL-biotin complexes from membrane fraction; c - bands of 3O-C_12_-HSL-biotin complexes from cytoplasmic fraction; u - bands of unbound components; IQGAP –IQ motif containing GTPase activating protein; GAPDH – glyceraldehydes-3-phosphate dehydrogenase.
